# Investigation of Uterine Fluid Extracellular Vesicles’ Proteomic Profiles Provides Novel Diagnostic Biomarkers of Bovine Endometritis

**DOI:** 10.3390/biom14060626

**Published:** 2024-05-25

**Authors:** Johanna Piibor, Andres Waldmann, Madhusha Prasadani, Ants Kavak, Aneta Andronowska, Claudia Klein, Suranga Kodithuwakku, Alireza Fazeli

**Affiliations:** 1 Institute of Veterinary Medicine and Animal Sciences, Estonian University of Life Sciences, Kreutzwaldi 62, 51006 Tartu, Estonia; piibor@emu.ee (J.P.); andres.valdmann@emu.ee (A.W.); madhusha.gamage@emu.ee (M.P.); ants.kavak@emu.ee (A.K.); suranga@emu.ee (S.K.); 2 Faculty of Veterinary Medicine, Latvia University of Life Sciences and Technologies, LV-3004 Jelgava, Latvia; 3 Institute of Animal Reproduction and Food Research, Polish Academy of Sciences, Juliana Tuwima 10, 10-748 Olsztyn, Poland; a.andronowska@pan.olsztyn.pl; 4 Friedrich-Loeffler-Institut, Federal Research Institute for Animal Health, Höltystr. 10, 31535 Neustadt, Germany; claudia.klein@fli.de; 5 Department of Animal Sciences, Faculty of Agriculture, University of Peradeniya, Peradeniya 20400, Sri Lanka; 6 Department of Pathophysiology, Institute of Biomedicine and Translational Medicine, University of Tartu, Ravila St. 14b, 50411 Tartu, Estonia; 7 Division of Clinical Medicine, School of Medicine & Population Health, University of Sheffield, Level 4, Jessop Wing, Tree Root Walk, Sheffield S10 2SF, UK

**Keywords:** endometritis, bovine uterine fluid, extracellular vesicles, proteomic changes, inflammation

## Abstract

Cow uterine infections pose a challenge in dairy farming, resulting in reproductive disorders. Uterine fluid extracellular vesicles (UF-EVs) play a key role in cell-to-cell communication in the uterus, potentially holding the signs of aetiology for endometritis. We used mass spectrometry-based quantitative shotgun proteomics to compare UF-EV proteomic profiles in healthy cows (H), cows with subclinical (SE) or clinical endometritis (CLE) sampled at 28–35 days postpartum. Functional analysis was performed on embryo cultures with the exposure to different EV types. A total of 248 UF-EV proteins exhibited differential enrichment between the groups. Interestingly, in SE, EV protein signature suggests a slight suppression of inflammatory response compared to CLE-UF-EVs, clustering closer with healthy cows’ profile. Furthermore, CLE-UF-EVs proteomic profile highlighted pathways associated with cell apoptosis and active inflammation aimed at pathogen elimination. In SE-UF-EVs, the regulation of normal physiological status was aberrant, showing cell damage and endometrial repair at the same time. Serine peptidase HtrA1 (HTRA1) emerged as a potential biomarker for SE. Supplementation of CLE- and SE-derived UF-EVs reduced the embryo developmental rates and quality. Therefore, further research is warranted to elucidate the precise aetiology of SE in cattle, and HTRA1 should be further explored as a potential diagnostic biomarker.

## 1. Introduction

Uterine health has a substantial impact on mammalian fertility, and an unhealthy uterine environment is a leading cause of infertility in farm animal species and humans. Uterine diseases in cattle compromise their welfare, diminish milk yields, increase veterinary expenditures, and elevate culling rates, with huge economic impacts [1,2,3]. Accurate diagnosis of uterine diseases assisted by in-depth knowledge of disease aetiology will facilitate proper management and ensures the continued success and profitability of dairy farms [4].

During the postpartum period, cows have a risk of getting uterine infections due to damage to the birth canal, failure to detach the placenta and often experience an upsurge of uterine microbial challenges. Naturally, cows are cured from uterine infection within one month post-calving. However, some can develop prolonged uterine infections, which can compromise their health and production [5]. Postpartum endometritis has been defined as an inflammation of the endometrium diagnosed three weeks or more after calving without systemic signs of illness, which is divided into clinical and subclinical categories. Clinical endometritis (CLE) is described as delayed involution, presence of mucopurulent discharge from the vagina, and a high number of polymorphonuclear cells (PMNs) in the endometrium, while in subclinical endometritis (SE), only the inflammation of the endometrium without clinical signs can be detected [6,7]. Nonetheless, endometritis is not exclusive to cattle. It occurs in various mammalian species such as humans, pigs, horses, and dogs. In these species, endometritis can be a primary cause of reproductive failure, frequently presenting with nonspecific or even absent clinical indications [8]. Strikingly, the chronic form of this ailment in humans resembles cattle’s SE, being generally asymptomatic and challenging to diagnose [9].

Efficient farm management relies heavily on good reproductive performance [10,11]. In cattle, it is of paramount importance to effectively prevent, diagnose, and manage endometritis to optimize fertility. However, the present methods to diagnose SE have strong limitations, which hinders the disease detection and treatment. For example, many factors can affect the sensitivity of the cytological diagnosis of SE such as sampling site [12], techniques of sampling [6,13], staining [6] and counting the cells [14], blood contamination [6], and parity of the cows [15], leading to false-negative real SE diagnosis. Thus, it is imperative to find a reliable liquid or any other simple biopsy sample method-based solid biomarker which is specific to SE and that will pave the way for developing even a ready-to-use kit in the future. In humans, the diagnosis of chronic endometritis relies on histopathology to detect inflammatory changes in the endometrium, but similar challenges exist regarding the sensitivity of disease detection as in cattle [16]. Thus, there is a huge avenue for new diagnostic methods and biomarker discovery to diagnose SE.

Extracellular vesicles (EVs) are lipid bilayer-containing nanoparticles that carry biomolecules, such as proteins, DNA, RNA, and metabolites, as internal or external (corona bound) cargo. The characteristics of EVs and their cargo reflect the physiological or pathological state of the originating cell [17]. The application of EVs and their content disparities in health and disease has been employed for diagnosing a range of human diseases, including cancer [18,19], neurological diseases [20], and infections [21,22]. In human studies, the exploration of EVs as potential biomarkers for reproductive diseases has been extensive. For instance, in conditions like preeclampsia, researchers have leveraged placental-derived EVs due to their distinctive cargo, suggesting the use of placenta-specific proteins (e.g., PLAP, GCF, PP13) [23,24] and miRNAs (e.g., chromosome 19 miRNA cluster) as potential biomarkers [25]. In cattle, EVs have also been researched as a potential biomarker to detect and monitor diseases in herds [26,27,28]. Some EV studies have induced uterine infection resulting in endometritis in vivo [26] or replicated the uterine conditions in cell culture [29,30]. These investigations have revealed variations in the miRNA or proteomic composition of EVs between healthy and inflamed conditions. Nevertheless, a universally accepted comprehensive understanding of the specific signatures across the studies is yet to be achieved.

Proteins that are involved in both inter- and intra-cellular signalling events are often good biomarkers since they reflect the state of cells in real time [31]. Thus, capturing these changes can be potentially developed into an assay for simple and quick detection of subclinical forms of the diseases [32]. However, up to this day, no such protein biomarker(s) for diagnosis of endometritis, specifically in SE, have been proposed. Cows and humans exhibit similarities in their chromosomal organization, amino acid sequence homology [33], and hormonal signalling mechanisms related to reproduction [34]. Therefore, possibly their proteins serve similar functions. This approach of comparative interspecies medicine could open new possibilities for advancing the comparative and translational aspects of studying EVs across different species. Therefore, the main aim of the current study was to investigate cell communications in terms of uterine fluid (UF)-EVs proteomic changes in healthy cows and cows with SE or CLE. Then, we evaluated the impact of disease UF-EVs on embryo developmental rates to determine the functional impact. Finally, we assessed the EV proteomic pathways of different endometritis forms which shed some light on the aetiological differences of two disease forms to propose potential proteomic biomarker(s) for SE detection.

## 2. Materials and Methods

The study design was evaluated and approved by the Committee for Conducting Animal Experiments at the Ministry of Rural Affairs, Estonia (Approval number 223 from 13 June 2022).

### 2.1. Selection of Cows

Multiparous Holstein cows (*Bos taurus*) were clinically evaluated at calving and three subsequent timepoints: between the ranges of 6–10, 21–26, and 35–42 days postpartum. Cows affiliated with difficult calving, stillbirths, birth of twins, metritis [5], fever [35], lameness [36], poor body condition [37] or any other clinical disease (except clinical hypocalcaemia) were excluded from the study (Supplementary File S1).

Ovarian function in the studied cows was monitored using ultrasonography (Supplementary File S1) and milk progesterone measurements by enzyme immunoassay following previously described protocols (Supplementary File S2) [38,39,40]. Cows with follicular or luteal cysts were excluded from the study.

The evaluation of uterine health was based on vaginal discharge characteristics (colour, proportion, and volume of pus) [41] and cytological evaluation [42] of cell pellet acquired from the UF. Details of the evaluation protocol and classification of the cow’s uterine health are provided in Supplementary File S2.

### 2.2. Collection of UF for EV Enrichment

The UF samples were collected from 3 healthy cows (88.0 ± 5.7 mL), 3 cows with SE (87.7 ± 5.8 mL), and 3 cows with CLE (101.3 ± 6.6 mL) between days 35 and 42 postpartum (*n* = 9). Briefly, the flushing was performed under lower sacral epidural anaesthesia using xylazine (0.05 mg/kg, Xylapan, Vetoquinol Biowet Sp z o.o, Gorzów Wielkopolski, Poland) diluted in 5 mL of saline. Both uterine horns were flushed with 50 mL of PBS (Dulbecco’s Phosphate-Buffered Saline, Sigma-Aldrich, Taufkirchen, Germany,) using a Foley embryo transfer catheter CH18 (Minitüb GmbH, Tiefenbach, Germany) and collected into a plastic tube as much as possible. Thereafter, the collected UF samples from both horns were pooled for each cow and transported on ice for processing within 2 to 3 h after collection.

### 2.3. Sample Preparation and Storage

Collected UF samples were subjected to differential centrifugation to remove cells, cell debris, apoptotic bodies, and other impurities. A total of three centrifugation steps were performed, whereby, after each step, the supernatant was transferred to another fresh tube: 250× *g* for 5 min, 2000× *g* for 10 min, and 10,000× *g* for 30 min (all at 4 °C). After completing all centrifugation steps, the sample was stored at −80 °C until EV enrichment.

### 2.4. UF-EV Enrichment

The UF-EV enrichment was performed as described previously [43]. The samples were concentrated at 4000 g in 4 °C using Amicon^®^ Ultra 15 mL centrifugal filters (10 kDa cut-off, Merck Millipore Ltd., Darmstadt, Germany) to 500 µL. Thereafter, the size exclusion chromatography (SEC) method was used to enrich UF-EVs from the concentrated UF samples. From the collected fractions of 500 µL during SEC, the fractions 6–9 contained EVs according to the nanoparticle tracking analysis (NTA) as described below. The EV sample aliquots were concentrated up to 500 µL and thereafter stored at −80 °C in protein low-binding tubes until further use.

### 2.5. Characterization of UF-EVs

The characterization of UF-EVs was performed following previously published protocol [43] in accordance with the International Society for Extracellular Vesicles guidelines [44]. Briefly, particle concentration and size were measured with an NTA ZetaView^®^ device (PMX 110 V3.0 instrument by Particle Metrix GmbH, Inning am Ammersee, Germany) coupled with ZetaView NTA software (v3.0). Transmission electron microscopy (TEM) was performed using 2% uranyl acetate (Polysciences, Warrington, PA, USA) on UF-EV-absorbed formvar/carbon-coated 200 mesh grids (Agar Scientific, Stansted, UK). Finally, the liquid chromatography–mass spectrometry (LC–MS/MS) analysed sample log-transformed label-free quantification (LFQ) abundances before and after EV purification was visualised using R (v4.1.0) using ggplot2 package (v3.5.1).

### 2.6. Analysis of UF-EV Total Proteomic Profile

All samples were processed in a randomized order. Precipitation of proteins was performed using trichloroacetic acid and sodium deoxycholate protocol as described [43]. The acquired purified peptides were reconstituted in 0.5% TFA for nano-LC/MS/MS. Final peptide injection amounts were determined by pre-analysing the twentyfold dilution of the final samples with LC–MS/MS, and an equal amount of each sample with the same protein concentration was injected for a final run.

The LC–MS/MS analysis was carried out by loading injected peptides to a 0.3 × 5 mm trap column (5 µm C18 particles, Dionex, CA, USA) using an Ultimate 3000 RSLCnano system (Dionex, CA, USA). Separated peptides were on-line electro-sprayed to a Q Exactive HF (Thermo Fisher Scientific) mass spectrometer.

The raw files of MS/MS output were processed with the MaxQuant software package (version 2.0.3.0). A search was performed against the UniProt (www.uniprot.org, accessed on 12. September 2023) *Bos taurus* proteome database (37,516 entries; downloaded: September 2022). All other parameters were used as described [43].

### 2.7. Validation with Western Blot Analysis

To verify the proteomics results, a Western blot analysis was carried out on cow UF isolated from healthy cows, cows with SE or CLE. For lysis and extraction of proteins, RIPA buffer (Thermo Fisher Scientific, Waltham, MA, USA) with protease inhibitor cocktail (cat. 535140, EMD Millipore Corp, Burlington, MA, USA) was used to process the sample as described [43]. The protein concentration was measured using a Pierce™ BCA Protein Assay Kit (cat. 23250, Thermo Fisher Scientific, Waltham, MA, USA) according to the manufacturer’s instructions. The absorbance of standards and samples was measured with a spectrophotometer (Ledetect 96 Microplate Reader, Biomed Dr. Wieser GmbH, Salzburg, Austria) at 540 nm wavelength, and protein concentrations were calculated accordingly.

The separation of proteins was performed in a 12% SDS-PAGE, blotted and blocked as described [43]. The primary antibody against HTRA1 (cat. PA5-23395, Thermo Fisher Scientific, Waltham, MA, USA) and β-actin (cat. 20536-1-AP, Proteintech Group Inc., Rosemont, IL, USA), in the concentrations of 1:1000 and 1:10,000, respectively, were used. The secondary antibody used was HRP—conjugated goat anti-rabbit IgG (1:10,000, cat. G-21234 Thermofisher Scientific, Waltham, MA, USA). The bands were detected (Supplementary File S3) by enhanced chemiluminescence (ECL; Amersham Pharmacia Biotech, Amersham, UK), imaged using an ImageQuant™ RT ECL™ machine coupled with IQuantCapture software v2.0 (GE Healthcare Bio-Sciences AB, Uppsala, Sweden) and quantified.

### 2.8. In Vitro Production of Embryos

#### 2.8.1. Embryo Culturing

The culturing of group embryo cultures was performed according to the protocol described [43]. Briefly, the cumulus–oocyte complexes (COCs) were aspirated from bovine ovaries acquired from the slaughterhouse. After aspiration, the COCs were evaluated, and only quality code 1 COCs were placed into IVM media (TCM-199 media supplemented with 0.8% fatty acid-free bovine serum albumin fraction V, 100 mM of pyruvate, 200 mM of L-glutamine, 10 mg/mL of gentamycin sulphate, 10 µg/mL of epidermal growth factor, and 1500 IU/mL of PG600). The isolated COCs were incubated at 38.8 °C in 6% CO_2_ for 22 to 24 h.

Frozen–thawed Holstein bull’s semen (Shimer 28179 DE363597637) was used to fertilize the matured COCs. The sperm concentration used was 2 million/mL in the IVF-TALP media, which was incubated with the matured COC groups at 38.8 °C in 6% CO2 for 18 h.

Cumulus cells were removed from presumptive zygotes by vortexing for 2 min, and denuded embryos were transferred to 500 µL of SOF media. The embryos were cultured at 38.8 °C in 6% CO_2_ and 6% O_2_ for 8 days. Embryos were morphologically evaluated at days 2, 5, 6, and 8 post-fertilization, and the developmental stages and blastocysts quality were assessed as previously described [45].

#### 2.8.2. Supplementation of UF-EVs to Embryo Cultures

The group embryo cultures were supplemented with the UF-EVs at day 2 post-fertilization in the concentration of 4.32 × 10^8^ particles/mL in the media [43]. The impact of UF-EVs on bovine embryo morphological development in group embryo cultures (*n* = 25) supplemented with pooled UF-EVs of cows with CLE (from 3 cows), cows with SE (from 3 cows), and healthy cows (from 3 cows) was evaluated and compared to controls (no EV supplementation). The cleavage rates were evaluated at day 2 before UF-EV supplementation and showed no difference between groups. The morula rates from cleaved embryos were evaluated at days 5 and 6 post-fertilization. The blastocyst rates from cleaved embryos and quality were evaluated at day 8 post-fertilization. The experiment was performed in 6 replicates of 25 zygotes per group.

### 2.9. Experimental Design and Data Analysis

**Figure 1:**
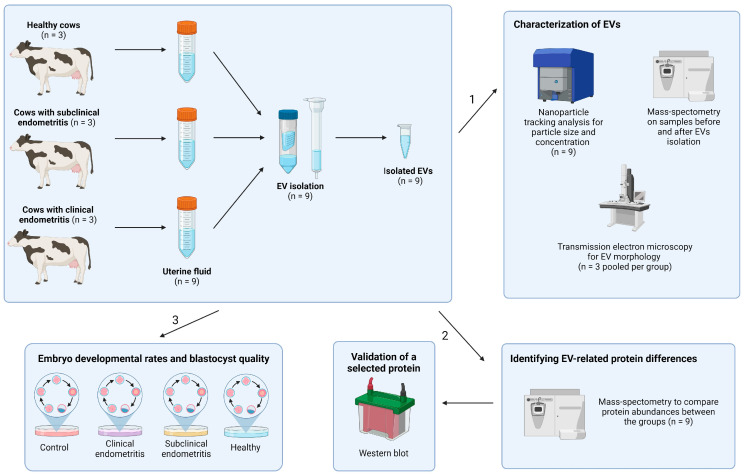
Study design. Collected uterine fluid (UF) extracellular vesicle (EV) samples were subjected to characterization with nanoparticle tracking analysis, mass-spectrometry, and transmission electron microscopy (1). UF-EV proteome was analysed using mass-spectrometry and chosen protein was validated with Western blot (2). Moreover, UF-EVs were supplemented to embryo cultures to evaluate the embryo developmental rates and blastocyst quality (3).

The overall experimental design of the experiments is depicted in Figure 1. In total, 3 healthy cows, 3 cows with SE, and 3 cows with CLE were randomly selected after the clinical and cytological evaluations described above. UF samples were collected from the cows between days 35 and 42 postpartum.

The EVs from the 9 cows’ UF samples were isolated using SEC-based methodology and characterized. GraphPad Prism v9.3.0.463 (GraphPad Software, San Diego, CA, USA) was used to plot the particle concentration profiles acquired from NTA analysis. Data are shown as mean ± SD. The differences in particle concentration and size were assessed between healthy cows, cows with SE or CLE with R (v4.1.0) using two-way ANOVA with post hoc Tukey multiple comparison test. The differences are statistically significant at *p* ≤ 0.05.

The processed LC–MS/MS data were further analysed with LFQ-analyst platform [46]. The differential enrichment analysis comparing the healthy cows, cows with SE or CLE was performed using k-nearest neighbour imputation method, and FDR was corrected according to the Benjamini–Hochberg method. The cut-off values were *p*-adjusted value at 0.05 and log2 at 1.5.

Pooled UF-EVs per group (*n* = 3) were supplemented to group embryo cultures in the concentration of 4.32 × 10^8^ particles/mL according to previously optimized protocol. The embryo developmental rates at days 5, 6, and 8 were evaluated and compared between groups—(1) control (no UF-EVs supplemented), (2) embryos supplemented with UF-EVs of cows with CLE, (3) embryos supplemented with UF-EVs of cows with SE, and (4) embryos supplemented with healthy cow UF-EVs. Additionally, blastocyst quality was evaluated on day 8 and compared between groups. The group differences were evaluated using Chi-square test in R (v4.1.0.). The differences are statistically significant at *p* < 0.05. The data are shown as mean ± standard deviation (SD).

## 3. Results

### 3.1. Characterization of UF-EVs

**Figure 2:**
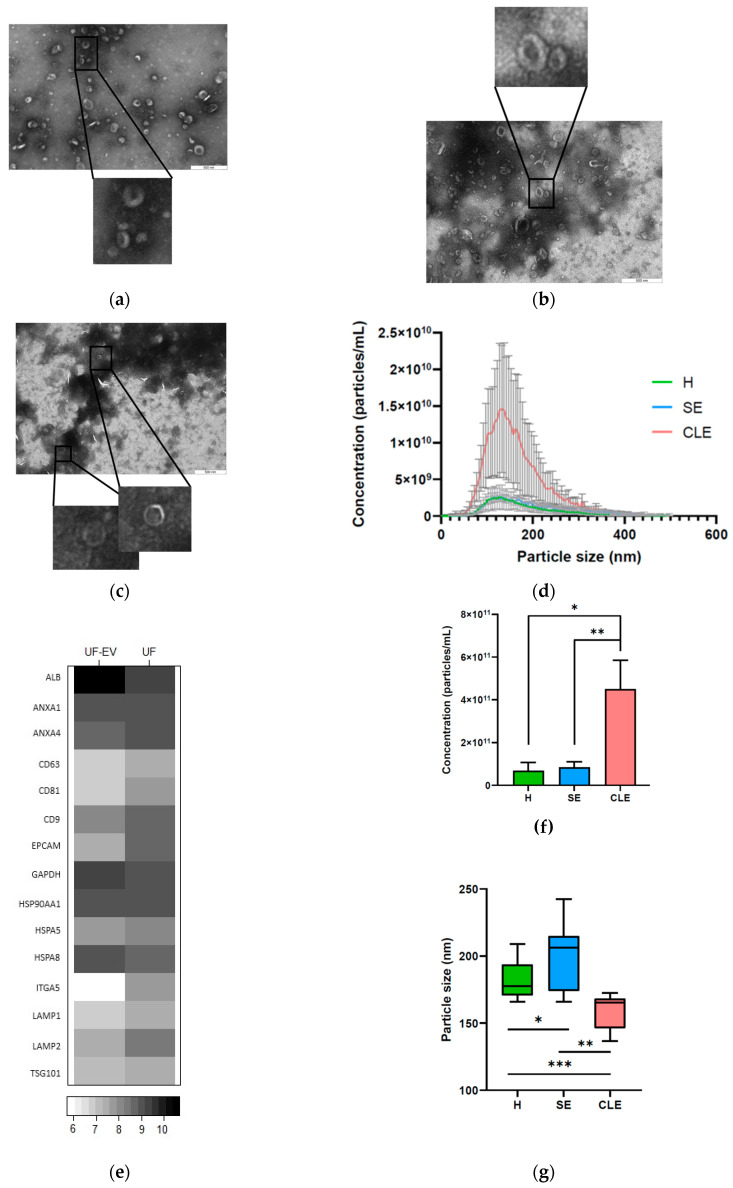
Characterization of uterine fluid (UF) extracellular vesicles (EVs). Cup-shaped structures were visualized with transmission electron microscopy from UF-EV samples of healthy (H) cows (**a**), cows with subclinical endometritis (SE) (**b**), and cows with clinical endometritis (CLE) (**c**). The particle size profile of UF-EVs showed particles in the range of 40 to 375 nm (**d**). After UF-EV purification, the protein enrichment was seen in some EV-related proteins, for example (**e**): Annexin (ANXA) 1, ANXA4, Cluster determinant (CD) 63, CD81, CD9, Epithelial cell adhesion molecule (EPCAM), Heat shock protein (HSP) 90AA1, HSPA5, Integrin subunit alpha 6 (ITAG6), Lysosomal-associated membrane protein (LAMP) 1, LAMP2 and Tumour susceptibility gene 101 (TSG101). The purification of UF-EVs depleted some known impurities in UF, such as albumin (ALB) and Glyceraldehyde 3-phosphate dehydrogenase (GAPDH). The particle concentrations were significantly different (*p* ≤ 0.05) between cows with CLE and H cows or cows with SE. * *p* = 0.01, ** *p* = 0.02 (**f**). The average particle sizes were significantly different between all the groups. * *p* < 0.05, ** *p* < 0.0001, *** *p* < 0.001 (**g**).

Cup-shaped vesicular structures were visualized in all the UF-EV samples with TEM, which is a characteristic morphology for the EVs (Figure 2a–c). The particle size profile obtained from NTA indicated that the measured particles fell within the size range of 40–375 nm, consistent with the typical size range of EVs (Figure 2d). The purification process of UF-EVs resulted in enrichment of EV-associated protein markers [47], including Annexin (ANXA) 1, ANXA4, Cluster determinant (CD) 63, CD81, CD9, Epithelial cell adhesion molecule (EPCAM), Heat shock protein (HSP) 90AA1, HSPA5, Integrin subunit alpha 6 (ITAG6), Lysosomal-associated membrane protein (LAMP) 1, LAMP2, and Tumour susceptibility gene 101 (TSG101). Conversely, the purification process led to depletion of albumin (ALB) and Glyceraldehyde 3-phosphate dehydrogenase (GAPDH), which are known impurities in UF (Figure 2e).

### 3.2. EV Particle Concentrations and Sizes Were Different in Different Endometritis Types

The concentration of particles in UF-EVs from cows with CLE (4.5 × 10^11^ ± 1.3 × 10^11^ particles/mL) was significantly higher (*p* ≤ 0.05) compared to healthy cows (6.9 × 10^10^ ± 3.8 × 10^10^ particles/mL) and cows with SE (8.4 × 10^10^ ± 2.6 × 10^10^ particles/mL) (Figure 2f). However, the average particle size of UF-EVs from cows with CLE (159.4 ± 12.3 nm) was significantly lower (*p* ≤ 0.05) compared to healthy cows (181.1 ± 13.0 nm) and cows with SE (201.0 ± 24.5 nm). Interestingly, the average particle size of SE-EVs was significantly higher (*p* ≤ 0.05) in comparison to healthy cows (Figure 2g).

### 3.3. UF-EV Proteomic Profiles Were Varied between Different Endometritis Forms

**Figure 3:**
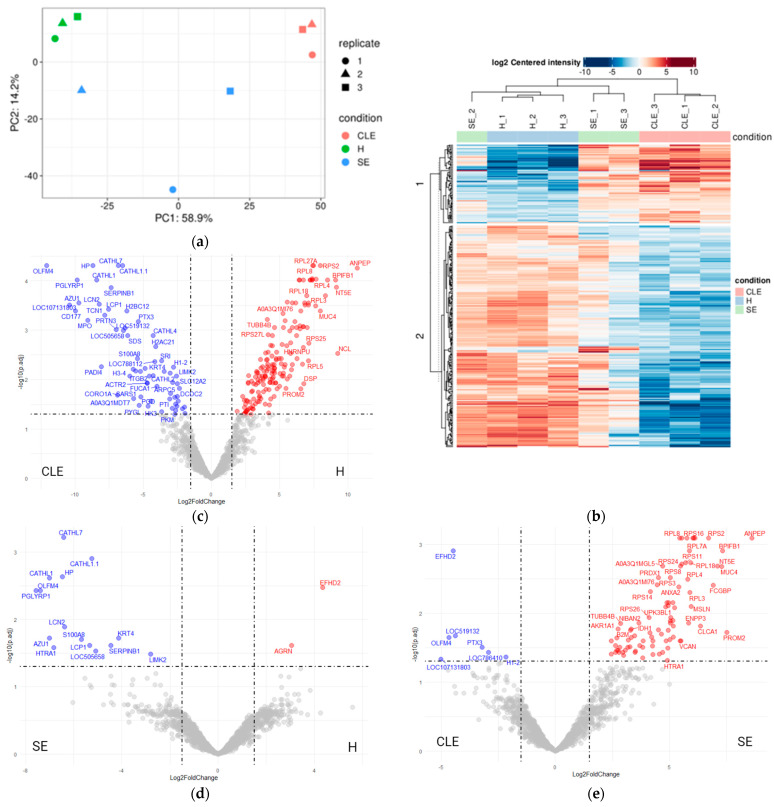
The uterine fluid (UF) extracellular vesicles (EVs) protein profile. The principal component analysis (PCA) showed separation of protein enrichment patterns between the different groups (**a**). Heatmap analysis showed the significant (*p* ≤ 0.05) protein enrichment patterns (**b**). A total of 169 UF-EV proteins were significantly enriched and 63 depleted in healthy (H; red) compared to clinical endometritis (CLE; blue) (**c**). Whereas, 2 UF-EV proteins were significantly enriched and 15 depleted in H (red) compared to subclinical endometritis (SE; blue) (**d**). When comparing SE (red) and CLE (blue), we identified 86 UF-EV proteins significantly enriched and 7 depleted in SE compared to CLE (**e**).

The principal component analysis (PCA) revealed a distinct separation of UF-EV protein enrichment patterns among healthy cows, cows with SE, and those with CLE (Figure 3a). However, there was notable variability within the SE-UF-EV samples, characterized by a median coefficient of variation of 65%.

Heatmap analysis highlighted significant differences in the differentially enriched UF-EV protein patterns obtained from cows with CLE compared to healthy cows and those with SE. Notably, the second cluster of proteins displayed depletion in cows with CLE compared to the other groups (Figure 3b). These proteins were mainly related to gene expression regulation, translation, cellular nitrogen compound metabolic processes, plasma membrane organization, and regulation of cell shape. The first cluster in the heatmap displayed increased enrichments of proteins in cases of both CLE and SE (Figure 3b), primarily associated with immune processes, cell apoptosis, and migration.

Furthermore, among a total of 1549 UF-EV proteins, a significant differentiation (*p* ≤ 0.05) was observed in 248 proteins across the identified groups within the dataset (Supplementary File S4). The analysis of differential enrichment indicated that 169 UF-EV proteins were significantly enriched (*p* ≤ 0.05), while 63 were depleted in healthy cows compared to cows with CLE (Figure 3c). Interestingly, 2 proteins were significantly enriched (*p* ≤ 0.05), while 15 were depleted in cows with SE when compared to healthy cows (Figure 3d). Moreover, in cows with SE or CLE, 86 UF-EV proteins were found to be significantly enriched (*p* ≤ 0.05) compared to healthy cows, while 7 proteins were markedly depleted in SE compared to those with CLE (Figure 3e).

### 3.4. The Varied Enrichment of Inflammatory Pathways in the Different Endometritis Forms

**Figure 4:**
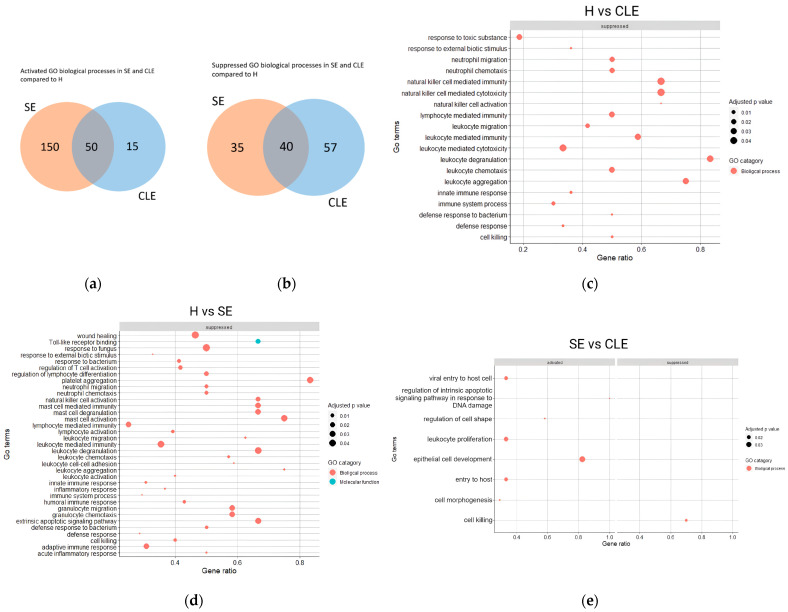
Gene ontology (GO) biological processes related to immune response in uterine fluid extracellular vesicles (UF-EVs) proteome. In total, 50 GO biological processes were similarly activated (**a**) and 40 suppressed (**b**) in both clinical endometritis (CLE) and subclinical endometritis (SE) compared to healthy cows (H). The immune response-related pathways showed that 15 pathways were similarly expressed in SE and CLE with 4 unique pathways in CLE (**c**) and 20 in SE when compared to H (**d**). In SE, the pathways showed activation of response to a pathogen, leukocyte proliferation, and repair of the endometrium, while apoptosis-related pathways were depleted compared to CLE (**e**).

The GO analysis revealed that only 23.2% of the identified biological processes were activated, and 30.3% of proteins were similarly suppressed in both SE and CLE-UF-EV when compared to healthy individuals (Figure 4a,b). From these biological processes, 15 immune-related pathways were consistently identified in both SE and CLE, with 4 unique pathways in CLE and 20 in SE when compared to healthy cows (Figure 4c–e). In the CLE-UF-EV proteomic profile, the identified pathways were linked to cell damage or apoptosis and active inflammation aimed at eliminating pathogens. Conversely, in the SE-UF-EV proteomic profile, the pathways related to the regulation of normal physiological status were aberrantly expressed, indicating cell damage, active responses to fungal and bacterial infection, and endometrial repair (Figure 4c–e).

### 3.5. HTRA1 Protein Can Be a Potential Biomarker for SE

**Figure 5:**
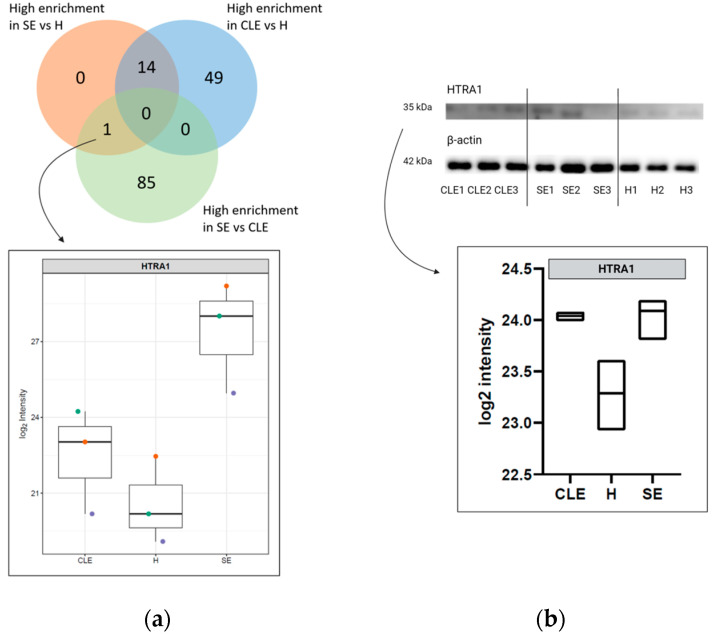
Potential biomarker for diagnosis of subclinical endometritis (SE). Only one protein, Serine peptidase HtrA1 (HTRA1), was unique with significantly enriched (*p* ≤ 0.05) in UF-EV samples of SE compared to samples acquired from healthy cows (H) and cows with clinical endometritis (CLE), which therefore could potentially be a biomarker for SE (**a**). The HTRA1 results were validated using Western blotting (WB) showing similarly higher HTRA1 protein intensities in SE compared to H (*p* = 0.03) and CLE (*p* = 0.07). The equal loading of total protein per sample was confirmed using β-actin (**b**).

The differential enrichment analysis revealed exclusively a single protein, Serine peptidase HtrA1 (HTRA1), that was significantly enriched (*p* ≤ 0.05) in cows with SE compared to healthy cows and cows with CLE (Figure 5a). Additionally, the HTRA1 protein’s validation through WB demonstrated greater enrichment in SE-UF samples compared to those from healthy cows (*p* ≤ 0.05). Nevertheless, there were no significant differences in HTRA1 expression between healthy cows and those with CLE, as well as between SE and CLE (*p* ≥ 0.05) (Figure 5b).

### 3.6. The Impact of Endometritis UF-EV on Embryo Development

**Figure 6:**
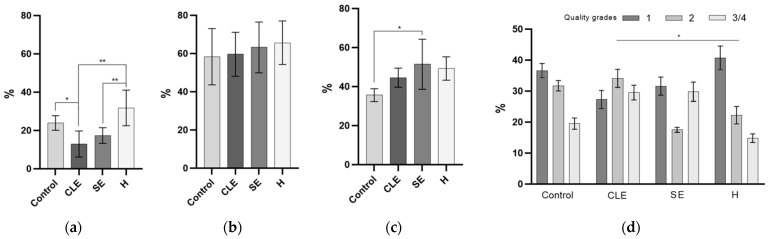
Developmental rates and quality of embryos supplemented with uterine fluid extracellular vesicles (UF-EVs). Morula rates at day 5 were significantly lower in embryos supplemented with UF-EVs from clinical endometritis (CLE) and subclinical endometritis (SE) compared to either embryos co-cultured with healthy cow (H) UF-EVs or the control group (**a**). Morula rates at day 6 showed no significant differences between the groups (**b**). Blastocyst rate at day 8 was significantly higher in embryos supplemented with UF-EVs from SE (**c**). The quality of blastocysts was significantly lower in embryos supplemented with UF-EVs from CLE compared to UF-EVs from H cows (**d**). * *p* < 0.05, ** *p* < 0.01.

The developmental rates of embryos supplemented with UF-EVs from cows with CLE, UF-EVs from cows with SE, and healthy cow UF-EVs were evaluated on days 5 and 6 (morula rates) and day 8 (blastocyst rates) of embryo culturing. On day 5, the morula rates of UF-EV groups with endometritis (CLE: 12.9 ± 6.8%; SE: 17.3 ± 4.1%) were significantly lower (*p* < 0.01) compared to the morula rates of embryos supplemented with healthy cow UF-EVs (31.7 ± 9.3%) (Figure 6a). The only group with significantly differentially lower (*p* < 0.05) morula rate compared to the control group (23.9 ± 3.8%) was the embryo group supplemented with UF-EVs of cows with CLE (Figure 6a). On day 6, the morula rates between the groups did not differ (Figure 6b). The blastocyst rates on day 8 did not differ when supplemented with UF-EVs from cows with CLE compared to control. However, the blastocyst rates between embryos supplemented with UF-EVs of cows with SE (51.4 ± 12.8%) were significantly higher (*p* < 0.05) compared to control (35.6 ± 3.3%; Figure 6c). The quality of blastocysts evaluated at day 8 was significantly lower (*p* < 0.05) in the UF-EVs-CLE group compared to when supplemented with healthy cow UF-EVs (Figure 6d).

## 4. Discussion

Postpartum uterine inflammatory conditions in cattle are linked to reproduction failures and have some shared features even with human uterine inflammatory conditions. In both species, the presence of endometritis without clear clinical manifestations poses a notable challenge, as its diagnosis is intricate. The impact of endometritis in cattle encompasses diminished conception rates, heightened embryo loss, and prolonged calving-to-conception intervals [48]. Similarly, in humans, endometritis is associated with compromised implantation and an elevated risk of miscarriage [9]. By acknowledging the similar negative effects of inflammation on fertility, employing a comparative and translational approach could potentially yield insights into the development of human endometritis as well. Therefore, in this endeavour, we used UF-EV proteomes as a mode to understand the disease aetiology and impact on the early embryonic events.

In the present study, the cow UF-EVs were successfully enriched from minimum invasively obtained uterine fluid samples meeting all ISEV 2023 guidelines [44]. The NTA measurements revealed significantly higher particle concentrations and smaller particle sizes in cows with CLE compared to healthy cows and cows with SE. However, there was no discernible difference in EV concentrations between healthy cows and cows with SE. Previous studies have observed that total EV concentration is elevated [49,50,51,52,53], while particle size is reduced in patients with various diseases compared to healthy controls [54]. This suggests that the ratio between EV concentration and size could potentially serve as an indicator of disease presence. However, some studies have found no differences in total particle concentration and size between diseased and healthy individuals [55,56]. Several variables could potentially contribute to the disparities observed among studies, including the size of the sample, methods used for UF collection, the quantity of UF collected, techniques for isolating/enriching EVs, and the methodology utilized for EV quantification. Furthermore, the number of EVs discharged depends on the state of the originating cells, where it has been observed that dying cells, prevalent during acute inflammation, release more EVs than healthy cells [57,58]. Similarly, in CLE, the microbiota in the uteri may also contribute to the significantly higher numbers of EVs. Therefore, the concentration and size of EVs could be influenced by the specific stage of the disease condition.

Potential biomarkers may be often overlooked in regular diagnostic platforms due to their extremely low concentrations in various sample types and falling below the sensitivity range of the instruments/kits/methods [59,60]. As a result, the detection of potential early disease biomarkers necessitates concentration or enrichment from the samples, making EVs a promising option for biomarker detection due to their concentrated nature. The cargo/proteins carried by EVs can serve as biomarkers, and their changes can be translated into assays for straightforward and rapid detection, particularly in farm or point-of-care settings with minimal invasiveness [32].

We observed differential enrichment of 248 UF-EV proteins, which accounts for 16% of all detected proteins between endometritis conditions and healthy cattle. Many of these proteins are implicated in immune-related processes, and their distinct expression patterns indicate varying immune responses in SE and CLE. When we examined the GO pathways associated with SE and CLE, we noticed a suppression of apoptosis response to inflammation in addition to increased leukocyte proliferation and cell development in SE compared to CLE. Overall, these responses suggest an aberrant inflammatory reaction in SE due to altered proinflammatory response. Notably, among women, the expression patterns of genes related to inflammatory responses, cell proliferation, and apoptosis processes in chronic endometritis endometrium resemble those observed in cattle [61]. For instance, chronic endometritis is characterized by a modified gene expression profile involving proinflammatory mediators like IL11, IL17, TGF-β, CCL4, IGFBP1, and CASP8, contrasting with the gene expression profile observed in normal fertile women during the implantation window. This alteration disrupts the delicate balance of maternal immune tolerance during implantation [62,63]. Nevertheless, additional research is essential to elucidate the specific factors and mechanisms underlying the varied inflammatory responses, which could pave the way for identifying preventive measures for endometritis and enhancing fertility across mammalian species.

The precise mechanisms that determine whether the normal inflammatory response following calving leads to either CLE or SE remain to be fully elucidated. Nonetheless, the notable differences in the proteomic makeup of UF-EVs between CLE and SE imply distinct origins for each condition. Certain literature works have proposed that these disparities stem from the cows’ capacity to adapt to metabolic challenges during the transition period. While bacterial dysbiosis and insufficient immune responses have been correlated with CLE, SE has not been linked to uterine dysbiosis. Instead, it has been shown to associate with negative energy balance [48]. Researchers have established a connection between inadequate adaptation to negative energy balance and impaired regulation of inflammation [64,65]. This is because immune cells in dairy cows, found across various tissues, regularly metabolize nutrients [66]. Moreover, metabolic dysfunction can influence the distribution of immune cells and impede immune responses [67]. Therefore, as a result, aberrant inflammation can occur even without the presence of pathogenic bacteria in the uterine environment [68,69].

In our study, we observed 17 proteins in UF-EVs that showed varying levels of enrichment between healthy cows and those with SE. Conversely, another research study has identified 186 proteins showing differential enrichment in UF between healthy cows and those with SE [70]. Moreover, a separate investigation revealed 330 proteins exhibiting differential enrichment in cell cultures between the group treated with lipopolysaccharide (LPS), an endotoxin recognized for inducing uterine inflammation, and the non-treated group [30]. Variations in the quantity/number of differentially enriched proteins may result from variances in the materials analysed, disparities in MS techniques, and differences in the methodology used to classify the SE. However, these contrasting findings may imply that diagnosing SE might require multiple protein markers, with a blend of variously expressed levels serving as diagnostic indicators. Nonetheless, our study identified HTRA1 as a significantly expressed protein in SE samples compared to both healthy cows and cows with CLE. Interestingly, similarly have been noted the distinct enrichment of HTRA1 in the LPS-treated group compared to the control, which is consistent with our observations [70]. Therefore, HTRA1 emerges as a promising biomarker for SE, facilitating differentiation between CLE and healthy cows. To validate or contest this hypothesis, future studies with larger sample sizes are imperative.

While the structure of HTRA1 has been well described [71,72], the activity remains to be completely elucidated. Previous investigations have described the involvement of HTRA1 in the WNT signalling pathway, wherein it influences cell homeostasis and proliferation by stabilizing the regulatory protein β-catenin [73]. For example, overexpression of HTRA1 in human embryonic kidney (HEK)293T cells led to a notable 60% reduction in their proliferation capacity [74]. Interestingly, in osteoarthritis, a multifactorial joint inflammatory disorder, the levels of HTRA1 mRNA were 7-fold elevated in the cartilage of early-stage osteoarthritis patients compared to healthy controls [75]. Furthermore, mouse tissues afflicted with late-stage osteoarthritis after destabilization surgery expressed HTRA1, which diminished the expression of transforming growth factor beta 1 (TGF-β1). TGF-β1 is a factor associated with cartilage repair [76,77]. This suggests a plausible role for HTRA1 in early tissue degeneration and disease progression. Notably, the exploration of HTRA1’s role in SE remains relatively uncharted. Future research is needed to decipher the role of HTRA1 in SE and its potential use as a biomarker.

Our data strongly indicate that UF-EVs from uteri affected by endometritis negatively influence embryo development, and endometritis is recognized for its detrimental effects on both endometrial function and embryo viability [29,61,78]. Embryos exposed to inflammatory mediators during embryo development were found to have fewer trophectoderm cells [78,79] where trophectoderm cells in conceptus are known to initiate uterine contact, a necessity to the events of implantation [80]. Hence, endometritis could potentially have direct or indirect effects on embryonic losses in cattle. The present study is the first to explore the influence of bovine UF-EVs from cows with CLE, SE, and healthy conditions on embryo developmental rates and quality. According to present data, a significant decline in morula rates on day 5 among embryos supplemented with UF-EVs from cows with endometritis compared to those supplemented with UF-EVs from healthy cows or the control group was noted. Moreover, when supplemented with UF-EVs from cows with CLE as opposed to healthy cows, the quality of blastocysts diminished. Thus, UF-EVs from cows with endometritis can impact embryo development, resulting in decreased developmental rates and quality. Consequently, lower pregnancy rates may not solely stem from functional differences in the endometrium; instead, they could also be partly attributed to reduced embryo quality, particularly in cases of CLE. At present, there is no recognized EV enrichment method that can separate EVs from different non-EV particles. As a result, various biomolecules within the UF may be co-enriched with EVs, potentially forming the EV protein corona [17,81], and be measured alongside UF-EV proteins. However, our study demonstrated the enrichment of EV-associated proteins and the decrease in known non-EV-related protein contaminants in the isolated samples compared to raw UF. This suggests that our EV enrichment method successfully achieved significant enrichment of EVs from bovine UF, thereby showing potential for further refinement towards the development of a bioassay platform. Additionally, given the similarities observed, we propose the cow as a potential animal model for studying the aetiology of human chronic endometritis in the future.

A limitation of this study is the small number of animals included in the analysis. Thus, future studies with larger sample sizes are warranted to confirm these findings.

## 5. Conclusions

The two forms of bovine endometritis exhibit distinctly different proteomic profiles in UF-EVs. Significantly, the varying enrichment of UF-EV proteins indicates an aberrant inflammatory response characteristic of SE as opposed to CLE. Our findings suggest that the pathogenesis of CLE and SE may follow divergent paths, suggesting two distinct mechanisms underlying disease progression. Among the subset of proteins showing differential enrichment, HTRA1 emerges as a potential candidate for diagnosing SE. Furthermore, we demonstrated that UF-EVs from endometritis uteri influence embryo developmental rates and quality. Specifically, UF-EVs from cows with CLE decrease morula rates and blastocyst quality. Further investigations are essential to elucidate the precise mechanisms behind SE development and to assess the viability of HTRA1 as a diagnostic biomarker.

## Data Availability

The data that support the findings of this study are available from the corresponding author upon reasonable request. The mass spectrometry proteomics data have been deposited to the ProteomeXchange Consortium via the PRIDE [82] partner repository with the dataset identifier PXD044219.

## Supplementary Materials

The following supporting information can be downloaded at: https://www.mdpi.com/article/10.3390/biom14060626/s1, Supplementary File S1: Clinical data of the cows used in the study. Supplementary File S2: Evaluation of study population. Supplementary File S3: Western blotting images of HTRA1 and β-actin, Supplementary File S4: Uterine fluid extracellular vesicles proteins of healthy cows, cows with subclinical and clinical endometritis identified in the dataset. Supplementary File S5: Raw Western blotting images of HTRA1 and β-actin.
