# Supplementary material for: Investigation of Uterine Fluid Extracellular Vesicles’ Proteomic Profiles Provides Novel Diagnostic Biomarkers of Bovine Endometritis
Source: Biomolecules. 2024 May 25;14(6):626. doi: 10.3390/biom14060626 (PMC11202259; doi:10.3390/biom14060626)
Supplement: Supplementary file 1 [file biomolecules-14-00626-s001.zip › biomolecules-3005193-supplementary/Additional file 1.pdf]

Additional file 1: Clinical data of the cows used in the study.

**Cows**	**NrL**	**Twins/ stillbirth/ difficult calving**	**Milk fever**	**T °C**	**6-10d char. of vaginal mucus**	**BCS**	**LMS**	**Acquired fluid amount (mL)**	**US evaluation of ovarian structures (mm)**	**35-42d char. of vaginal mucus**	**Milk P4 concentration (ng/mL)**	**Uterine cytology (PMN%)**
Healthy cow 1 (H1)	2	None	None	38.8	Clear odorless	3.75	0	84	RO: CL 25.0, F 14.2.<br>LO: CL 26.2.	Clear odorless (score 0)	14.90	0
Healthy cow 2 (H2)	2	None	None	38.5	Reddish-brown odorless	3.75	0	96	RO: CL 32.5, F 2x 10.0.<br>LO: CL 24.0, F 17.8.	Clear odorless (score 0)	12.01	0.5
Healthy cow 3 (H3)	1	None	None	37.2	Clear odorless	3.25	0	84	RO: F 4x 0.7, F 1.0.<br>LO: CL 35.7, F 13.7.	Clear odorless (score 0)	11.64	0
Cow with subclinical endometritis 1 (SE1)	2	None	None	38.5	Clear with flakes odorless	3.5	0	80	RO: F 17.1.<br>LO: F 2x 8.0, CL 26.2.	Clear odorless (score 0)	5.03	8.5
Cow with subclinical endometritis 2 (SE2)	1	None	None	38.6	Clear odorless	3.5	0	94	RO: CL 22.6, F 17.4, F 15.4, F 14.3, F 10.6, F 7.2.<br>LO: F 9.0, F 6.1.	Clear odorless (score 0)	17.74	9.0
Cow with subclinical endometritis 3 (SE3)	1	None	None	37.9	Reddish-brown odorless	3.5	0	89	RO: CL 38.8, F 19.0.<br>LO: F 21.1.	Clear with flakes (score 1)	14.64	26.5
Cow with clinical endometritis 1 (CLE1)	2	None	Yes	38.5	Clear odorless	3.5	0	110	RO: CL 18.8, F 9.0.<br>LO: CL 18.5, F 18.8.	White foul smelling (score 2)	11.60	Too many to count

Cow with clinical endometritis 2 (CLE2)	2	None	None	38.4	Clear with pink flakes odorless	3.5	0	100	RO: F 15.8, F 9.4, F 0.8. LO: CL 21.2, CL 19.1, F 0.7, F 0.8.	White foul smelling (score 2)	13.47	Too many to count
Cow with clinical endometritis 3 (CLE3)	2	None	None	38.1	Clear with white flakes odorless	3.5	0	94	RO: F 2x 9.7. LO: F 25.3, F 18.8.	Brownish with > 50% white pus foul smelling (score 2)	0	Too many to count

NrL: Number of lactations past before calving; T°C: rectal temperature; char.: characteristics; 6-10d: 6 to 10 days postpartum; BCS: body condition score; LMS: lameness score; US: ultrasonography; P4: progesterone; PMN%: proportion of polymorphonuclear neutrophils; RO: right ovary; LO: left ovary; CL: *corpus luteum*; F: follicle.
