# Supplementary material for: Investigation of Uterine Fluid Extracellular Vesicles’ Proteomic Profiles Provides Novel Diagnostic Biomarkers of Bovine Endometritis
Source: Biomolecules. 2024 May 25;14(6):626. doi: 10.3390/biom14060626 (PMC11202259; doi:10.3390/biom14060626)
Supplement: Supplementary file 1 [file biomolecules-14-00626-s001.zip › biomolecules-3005193-supplementary/Additional file 2.pdf]

Additional file 2: Evaluation of the study population

Protocol for evaluation of the oestrous cycle using milk progesterone measurements by an enzyme immunoassay

To negate the effect of the time of milk extraction on the milk progesterone concentration, milk samples were collected not earlier than 3 h after previous milking [38], that is between 3 and 5h after morning milking. Milk was withdrawn by stripping and collected in plastic tubes containing potassium dichromate for preservation. Samples were frozen at –80 °C until progesterone analysis.

Concentrations of progesterone in milk were measured by an enzyme immunoassay [39], which was modified by using the secondary antibody technique. The specificity of the monoclonal antibody and assay have previously been reported [40]. All milk samples were analysed on one microplate. The intra-assay coefficients of variation were < 10%. The limit of Se using a 20 µL sample was < 0.5 ng/mL.

Unless mentioned otherwise, all used materials were purchased from Sigma Chemical Co. (St. Louis, MO, USA). The goat anti-mouse antibody (3 µg/mL; A/S LabAs, Tartu, Estonia) solution was pipetted to the EIA plate (Nunc Immuno Plate F96 Maxisorp) wells, followed by overnight incubation at 4 °C with the plate covered. The next day, the plate was brought to room temperature (RT) and washed twice with PBST (0.15M 1L PBS with 0.5 mL Tween-20). Stabilizing solution (50 g/L sucrose, 5 g/L bovine serum albumin, 0.2 g/L thimerosal in PBS) was added to the wells and incubated for 5 min at RT. After discarding the solution, the plate was dried at RT, sealed with tape, and stored at 4 °C until further use.

To determine milk progesterone levels, the coated EIA plate, reagents and milk samples equilibrated to RT. Working solutions of 1:1875 anti-progesterone monoclonal antibody 9C11 (MAb 9C11) and 1:1000 progesterone-horseradish peroxidase conjugate (P-HRP) in PBSTC were prepared. Each well received 20 µL of milk samples or progesterone standards in milk (0, 1, 2.5, 5, 10, 30 and 60 ng/mL), followed by 100 µL of MAb 9C11 and P-HRP working solutions. After sealing with tape, the plate was shaken at RT for 40 min. Subsequently, the plate was washed 3x with PBST, and 200 µL of chromogen-substrate solution (200

µL of 3.3’.5.5’-tetramethylbenzidine 10 mg/mL and 20 µL of 10% H2O2 in 20 mL of 0.1M acetate/citrate buffer) were added to each well. The colour reaction was halted after 30 min of incubation at RT in the dark by adding 100 µL of 3M H2SO4 into each well. Optical densities were read at 450 nm with MultiskanTM FC 1.01.17 (Thermo Fisher Scientific, Waltham, MA, USA), and the progesterone concentrations (Supplementary file 1) were determined through 4-parameter curve fit calculation.

Evaluation of uterine health status

Evaluation of vaginal mucus characteristics

Collected vaginal mucus characteristics were evaluated using a gradings system previously described in Williams et al. 2005 [41]. Briefly, the vaginal mucus colour, proportion and volume of pus was assessed and scored into four categories: clear or translucent mucus (score 0), mucus that contained white or off-white flecks of pus (score 1), < 50 mL exudate contained ≤ 50% white or off-white mucopurulent pus (score 2), and > 50 mL exudate contained white or yellow purulent pus (score 3).

Cytological evaluation of uterine fluid

Cytology slides were prepared from the cell pellets acquired after the initial centrifugation step of differential centrifugation described in the manuscript. These slides were subsequently stained and counted according to Valdmann et al. 2022 [42]. To elaborate, two cytological examination slides were prepared from the acquired cell pellet. The slides were immediately fixed under a current of warm air and the May-Grünwald-Giemsa staining protocol was followed. Firstly, the slides were placed in May-Grünwald stain (VWR Prolabo Chemicals, Leuven, Belgium) for 5 min, then transferred into diluted Giemsa stain (VWR Prolabo Chemicals, Leuven, Belgium) for 25 min and finally washed with distilled water. The slides were visualized under a light microscope (Olympus BX51; Olympus, Tokyo, Japan) at 1000x magnification. For each stained slide, a total of 100 epithelial cells or PMNs were counted, and the average percentage of PMNs relative to all cells was calculated.

Classification of uterine health status

Uterine health status was classified into three categories based on the absence or presence of clinical and cytological evidence of inflammation: healthy cows, cows with SE and cows with CLE. Cows were considered healthy when during the clinical examination no clinical diseases were diagnosed, the characteristics of vaginal mucus was scored 0 at sampling, and the proportion of PMNs in the UF sample was <1%. SE was diagnosed in cows that did not suffer any clinical disease, had vaginal mucus characteristics scored 0 or 1, and had a PMN proportion >5%. Cows were designated as having CLE if no clinical diseases were diagnosed during clinical examinations (except clinical hypocalcaemia), vaginal mucus characteristics were scored 2 or 3, and the PMN proportion was too high to be counted on the slides.
