# Supplementary material for: Investigation of Uterine Fluid Extracellular Vesicles’ Proteomic Profiles Provides Novel Diagnostic Biomarkers of Bovine Endometritis
Source: Biomolecules. 2024 May 25;14(6):626. doi: 10.3390/biom14060626 (PMC11202259; doi:10.3390/biom14060626)
Supplement: Supplementary file 1 [file biomolecules-14-00626-s001.zip › biomolecules-3005193-supplementary/Additional file 3.pdf]

HTRA1

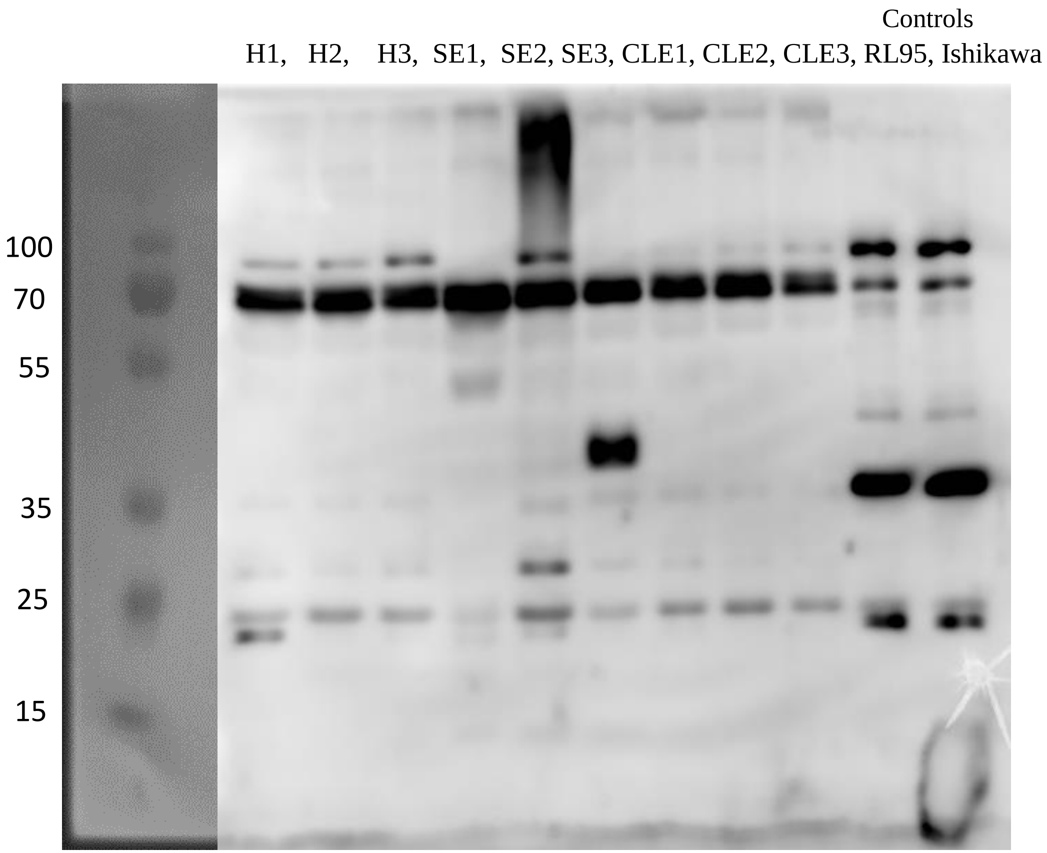

β-actin

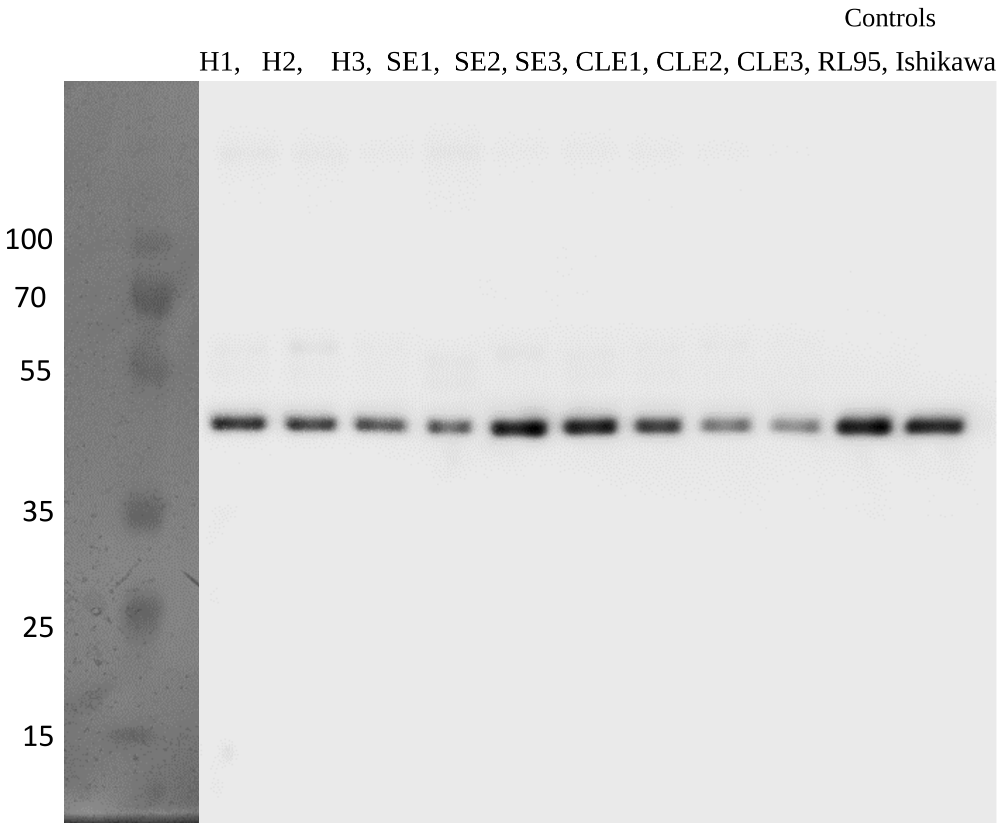

H = healthy cows
SE = cows with subclinical endometritis
CLE = cows with clinical endometritis
