# Supplementary material for: Investigation of Uterine Fluid Extracellular Vesicles’ Proteomic Profiles Provides Novel Diagnostic Biomarkers of Bovine Endometritis
Source: Biomolecules. 2024 May 25;14(6):626. doi: 10.3390/biom14060626 (PMC11202259; doi:10.3390/biom14060626)
Supplement: Supplementary file 1 [file biomolecules-14-00626-s001.zip › biomolecules-3005193-supplementary/Additional file 4.pdf]

**Additional file 4:** Uterine fluid (UF) extracellular vesicles (EVs) proteins of healthy cows (H), cows with subclinical (SE) and clinical endometritis (CLE) identified in the dataset. The significantly differentially enriched (P ≤ 0.05) proteins are marked as TRUE between the specified comparisons.

| Protein ID | Protein name | Log2 fold change | | | P-adjusted value | | | Significant | | |
|---|---|---|---|---|---|---|---|---|---|---|
| | | H vs CLE | H vs SE | SE vs CLE | H vs CLE | H vs SE | SE vs CLE | H vs CLE | H vs SE | SE vs CLE |
| **A0A3Q1LFQ2** | Uncharacterized protein | -0.705 | -0.0937 | -0.611 | 0.606 | 0.959 | 0.684 | FALSE | FALSE | FALSE |
| **A0A3Q1LI44** | Ig-like domain-containing protein | 0.225 | 0.9 | -0.675 | 0.895 | 0.633 | 0.716 | FALSE | FALSE | FALSE |
| **A0A3Q1LJT1** | Ig-like domain-containing protein | -2.03 | -0.487 | -1.54 | 0.324 | 0.865 | 0.486 | FALSE | FALSE | FALSE |
| **A0A3Q1LPG0** | Uncharacterized protein | -1.25 | -0.116 | -1.14 | 0.44 | 0.96 | 0.529 | FALSE | FALSE | FALSE |
| **A0A3Q1LQ52** | SEA domain-containing protein | 0.248 | -1.93 | 2.18 | 0.885 | 0.26 | 0.156 | FALSE | FALSE | FALSE |
| **A0A3Q1LWV8** | Ig-like domain-containing protein | -0.733 | -0.453 | -0.28 | 0.684 | 0.852 | 0.894 | FALSE | FALSE | FALSE |
| **A0A3Q1MDT7** | Histone H4 | -5.72 | -3.76 | -1.96 | 0.0243 | 0.253 | 0.475 | TRUE | FALSE | FALSE |
| **A0A3Q1MGL5** | Uncharacterized protein | 5.57 | 0.866 | 4.71 | 0.00027 | 0.516 | 0.00207 | TRUE | FALSE | TRUE |
| **A0A3Q1MI76** | Uncharacterized protein | 5.25 | 0.779 | 4.47 | 0.000463 | 0.591 | 0.0039 | TRUE | FALSE | TRUE |
| **A0A3Q1ML26** | Ig-like domain-containing protein | -0.772 | -0.185 | -0.587 | 0.51 | 0.917 | 0.663 | FALSE | FALSE | FALSE |
| **A0A3Q1MSF6** | Ig-like domain-containing protein | -1.02 | 0.172 | -1.19 | 0.426 | 0.933 | 0.382 | FALSE | FALSE | FALSE |
| **A0A3Q1N072** | Uncharacterized protein | -3.03 | -0.632 | -2.4 | 0.00742 | 0.633 | 0.0529 | TRUE | FALSE | FALSE |

| | | | | | | | | | | |
|---|---|---|---|---|---|---|---|---|---|---|
| **A4GALT** | Alpha 1 4-galactosyltransferase | 0.158 | 1.49 | -1.33 | 0.9 | 0.279 | 0.277 | FALSE | FALSE | FALSE |
| **AARS1** | Alanine-RNA ligase | -0.192 | 0.432 | -0.624 | 0.895 | 0.802 | 0.68 | FALSE | FALSE | FALSE |
| **ABCA13** | ATP binding cassette subfamily A member 13 | 0.192 | 0.321 | -0.129 | 0.863 | 0.788 | 0.903 | FALSE | FALSE | FALSE |
| **ABCB1** | Uncharacterized protein | -0.449 | 1.24 | -1.69 | 0.76 | 0.431 | 0.261 | FALSE | FALSE | FALSE |
| **ABCE1** | ABCE1 protein | -0.183 | 0.432 | -0.615 | 0.874 | 0.709 | 0.561 | FALSE | FALSE | FALSE |
| **ABCF1** | ATP binding cassette subfamily F member 1 | -0.468 | 1.41 | -1.88 | 0.738 | 0.353 | 0.194 | FALSE | FALSE | FALSE |
| **ABHD14B** | Putative protein-lysine deacylase ABHD14B | 0.944 | 0.0756 | 0.868 | 0.554 | 0.969 | 0.62 | FALSE | FALSE | FALSE |
| **ABI1** | Abl interactor 1 | 0.546 | 1.24 | -0.697 | 0.654 | 0.35 | 0.586 | FALSE | FALSE | FALSE |
| **ABRACL** | Costars family protein ABRACL | 0.158 | 1.94 | -1.79 | 0.917 | 0.285 | 0.277 | FALSE | FALSE | FALSE |
| **ACAT2** | Acetyl-CoA acetyltransferase 2 | -3.6 | -3.29 | -0.31 | 0.0787 | 0.236 | 0.901 | FALSE | FALSE | FALSE |
| **ACE** | Angiotensin-converting enzyme | -0.207 | 0.452 | -0.66 | 0.885 | 0.758 | 0.619 | FALSE | FALSE | FALSE |
| **ACLY** | ATP-citrate synthase | -2.21 | -2.8 | 0.586 | 0.227 | 0.244 | 0.803 | FALSE | FALSE | FALSE |
| **ACO1** | Cytoplasmic aconitate hydratase | 4.6 | 1.85 | 2.75 | 0.0217 | 0.416 | 0.217 | TRUE | FALSE | FALSE |
| **ACSL3** | Acyl-CoA synthetase long chain family member 3 | -1.12 | -0.721 | -0.397 | 0.362 | 0.629 | 0.798 | FALSE | FALSE | FALSE |

| | | | | | | | | | | |
|---|---|---|---|---|---|---|---|---|---|---|
| **ACSL4** | Acyl-CoA synthetase long chain family member 4 | 3.54 | 0.275 | 3.26 | 0.0215 | 0.902 | 0.0646 | TRUE | FALSE | FALSE |
| **ACTG1** | Actin cytoplasmic 2 | -1.3 | -1.52 | 0.219 | 0.254 | 0.274 | 0.875 | FALSE | FALSE | FALSE |
| **ACTN1** | Alpha-actinin-1 | -2.73 | -2.79 | 0.0592 | 0.246 | 0.323 | 0.985 | FALSE | FALSE | FALSE |
| **ACTN4** | Alpha-actinin-4 | -2.18 | -2.4 | 0.217 | 0.11 | 0.224 | 0.898 | FALSE | FALSE | FALSE |
| **ACTR1A** | Actin related protein 1A | 1.32 | 0.605 | 0.718 | 0.235 | 0.659 | 0.566 | FALSE | FALSE | FALSE |
| **ACTR2** | Actin-related protein 2 | -4.71 | -3.12 | -1.59 | 0.0119 | 0.232 | 0.419 | TRUE | FALSE | FALSE |
| **ACTR3** | Actin-related protein 3 | -2.76 | -2.35 | -0.413 | 0.0767 | 0.253 | 0.841 | FALSE | FALSE | FALSE |
| **ADA** | Adenosine deaminase | -1.62 | -2.84 | 1.22 | 0.36 | 0.236 | 0.527 | FALSE | FALSE | FALSE |
| **ADAM10** | Disintegrin and metalloproteinase domain-containing protein 10 | -0.794 | -0.348 | -0.445 | 0.494 | 0.822 | 0.748 | FALSE | FALSE | FALSE |
| **ADAM8** | ADAM metallopeptidase domain 8 | -1.33 | 1.26 | -2.58 | 0.307 | 0.395 | 0.0797 | FALSE | FALSE | FALSE |
| **ADAM9** | ADAM metallopeptidase domain 9 | -1.12 | -0.882 | -0.235 | 0.306 | 0.484 | 0.858 | FALSE | FALSE | FALSE |
| **ADAMTS7** | ADAM metallopeptidase with thrombospondin type 1 motif 7 | 0.175 | -0.368 | 0.544 | 0.881 | 0.76 | 0.618 | FALSE | FALSE | FALSE |

| | | | | | | | | | | |
|---|---|---|---|---|---|---|---|---|---|---|
| **ADGRG3** | Adhesion G protein-coupled receptor G3 | 0.151 | 1.49 | -1.34 | 0.941 | 0.538 | 0.562 | FALSE | FALSE | FALSE |
| **ADH5** | Alcohol dehydrogenase class-3 | -0.179 | -0.223 | 0.0441 | 0.881 | 0.867 | 0.975 | FALSE | FALSE | FALSE |
| **ADRM1** | Proteasomal ubiquitin receptor ADRM1 | -1.19 | -0.695 | -0.493 | 0.354 | 0.652 | 0.747 | FALSE | FALSE | FALSE |
| **ADSL** | Adenylosuccinate lyase | -1.59 | -0.73 | -0.857 | 0.119 | 0.553 | 0.438 | FALSE | FALSE | FALSE |
| **ADSS2** | Adenylosuccinate synthetase isozyme 2 | -0.924 | -1.56 | 0.635 | 0.633 | 0.459 | 0.784 | FALSE | FALSE | FALSE |
| **AFDN** | Afadin adherens junction formation factor | 0.158 | 1.14 | -0.982 | 0.892 | 0.339 | 0.362 | FALSE | FALSE | FALSE |
| **AGO1** | Argonaute RISC component 1 | 0.411 | 1.47 | -1.06 | 0.789 | 0.365 | 0.489 | FALSE | FALSE | FALSE |
| **AGO2** | Protein argonaute-2 | 1.43 | 1.33 | 0.0978 | 0.208 | 0.326 | 0.942 | FALSE | FALSE | FALSE |
| **AGR2** | Anterior gradient 2 protein disulphide isomerase family member | 5.79 | 3.36 | 2.43 | 0.00181 | 0.16 | 0.177 | TRUE | FALSE | FALSE |
| **AGRN** | Agrin | 1.58 | 3.05 | -1.47 | 0.0929 | 0.0244 | 0.17 | FALSE | TRUE | FALSE |
| **AHCY** | Adenosylhomocysteinase | -1.11 | -1.81 | 0.693 | 0.381 | 0.257 | 0.636 | FALSE | FALSE | FALSE |
| **AHCYL1** | Adenosylhomocysteinase like 1 | -0.153 | 0.135 | -0.288 | 0.895 | 0.933 | 0.825 | FALSE | FALSE | FALSE |
| **AHNAK** | AHNAK nucleoprotein | 4.33 | 1.45 | 2.89 | 0.0245 | 0.519 | 0.179 | TRUE | FALSE | FALSE |

| | | | | | | | | | | |
|---|---|---|---|---|---|---|---|---|---|---|
| **AHNAK2** | AHNAK nucleoprotein 2 | 0.582 | 1.69 | -1.1 | 0.633 | 0.256 | 0.372 | FALSE | FALSE | FALSE |
| **AIDA** | Axin interactor dorsalization associated | 0.158 | 1.63 | -1.47 | 0.9 | 0.257 | 0.237 | FALSE | FALSE | FALSE |
| **AIMP1** | Aminoacyl tRNA synthetase complex interacting multifunctional protein 1 | 1.45 | 1.26 | 0.187 | 0.26 | 0.393 | 0.901 | FALSE | FALSE | FALSE |
| **AIMP2** | Aminoacyl tRNA synthase complex-interacting multifunctional protein 2 | 2.9 | -0.21 | 3.11 | 0.0958 | 0.938 | 0.116 | FALSE | FALSE | FALSE |
| **AK1** | Adenylate kinase isoenzyme 1 | -0.415 | 0.308 | -0.724 | 0.74 | 0.852 | 0.584 | FALSE | FALSE | FALSE |
| **AKAP13** | A-kinase anchoring protein 13 | 0.361 | -0.0796 | 0.44 | 0.738 | 0.956 | 0.716 | FALSE | FALSE | FALSE |
| **AKAP9** | A-kinase anchoring protein 9 | 1.43 | 1.44 | -0.00822 | 0.597 | 0.647 | 0.997 | FALSE | FALSE | FALSE |
| **AKR1A1** | Aldo-keto reductase family 1 member A1 | 1.15 | -1.6 | 2.76 | 0.229 | 0.232 | 0.0171 | FALSE | FALSE | TRUE |
| **AKR1B1** | Aldo-keto reductase family 1 member B1 | -0.0445 | -3.57 | 3.52 | 0.976 | 0.0943 | 0.0356 | FALSE | FALSE | TRUE |
| **AKR7A2** | Uncharacterized protein | -1.04 | 0.809 | -1.85 | 0.593 | 0.725 | 0.347 | FALSE | FALSE | FALSE |

| | | | | | | | | | | |
|---|---|---|---|---|---|---|---|---|---|---|
| **ALDH16A1** | Aldehyde dehydrogenase family 16 member A1 | -0.532 | 0.594 | -1.13 | 0.644 | 0.657 | 0.332 | FALSE | FALSE | FALSE |
| **ALDH1A1** | Aldehyde dehydrogenase 1A1 | -1.1 | -0.667 | -0.428 | 0.389 | 0.666 | 0.791 | FALSE | FALSE | FALSE |
| **ALDH2** | Aldehyde dehydrogenase mitochondrial | -0.297 | 1.51 | -1.81 | 0.848 | 0.332 | 0.216 | FALSE | FALSE | FALSE |
| **ALDH3A2** | Aldehyde dehydrogenase | 4.18 | 2.91 | 1.27 | 0.0804 | 0.316 | 0.654 | FALSE | FALSE | FALSE |
| **ALDH3B1** | Aldehyde dehydrogenase family 3 member B1 | 4.48 | 2.4 | 2.08 | 0.00673 | 0.244 | 0.228 | TRUE | FALSE | FALSE |
| **ALDH9A1** | 4-trimethylaminobutyraldehyde dehydrogenase | 0.237 | -1.55 | 1.78 | 0.886 | 0.332 | 0.229 | FALSE | FALSE | FALSE |
| **ALDOA** | Fructose-bisphosphate aldolase | -1.14 | -1.16 | 0.0189 | 0.352 | 0.397 | 0.988 | FALSE | FALSE | FALSE |
| **ALDOC** | Fructose-bisphosphate aldolase | -1.38 | -0.143 | -1.23 | 0.262 | 0.938 | 0.35 | FALSE | FALSE | FALSE |
| **ALPL** | Alkaline phosphatase tissue-nonspecific isozyme | 1.41 | -1.86 | 3.28 | 0.28 | 0.257 | 0.0308 | FALSE | FALSE | TRUE |
| **ALYREF** | THO complex subunit 4 | 0.379 | 1.01 | -0.633 | 0.737 | 0.396 | 0.592 | FALSE | FALSE | FALSE |

| | | | | | | | | | | |
|---|---|---|---|---|---|---|---|---|---|---|
| **ANKFY1** | Ankyrin repeat and FYVE domain containing 1 | -0.659 | 0.527 | -1.19 | 0.569 | 0.696 | 0.326 | FALSE | FALSE | FALSE |
| **ANKIB1** | RBR-type E3 ubiquitin transferase | -2.25 | -2.5 | 0.247 | 0.115 | 0.224 | 0.889 | FALSE | FALSE | FALSE |
| **ANO6** | Anoctamin | 0.158 | 1.36 | -1.2 | 0.895 | 0.285 | 0.295 | FALSE | FALSE | FALSE |
| **ANP32A** | Acidic leucine-rich nuclear phosphoprotein 32 family member A | 0.417 | 1.03 | -0.615 | 0.755 | 0.48 | 0.678 | FALSE | FALSE | FALSE |
| **ANPEP** | Aminopeptidase | 10.7 | 2.07 | 8.63 | 0.000055 | 0.3 | 0.000811 | TRUE | FALSE | TRUE |
| **ANXA1** | Annexin A1 | -0.868 | -0.205 | -0.663 | 0.389 | 0.883 | 0.559 | FALSE | FALSE | FALSE |
| **ANXA11** | Annexin A11 | -2.16 | -0.0537 | -2.11 | 0.0548 | 0.971 | 0.0938 | FALSE | FALSE | FALSE |
| **ANXA2** | Annexin A2 | 6.12 | 0.714 | 5.41 | 0.000678 | 0.69 | 0.00418 | TRUE | FALSE | TRUE |
| **ANXA3** | Annexin A3 | -0.614 | -1.1 | 0.49 | 0.662 | 0.467 | 0.765 | FALSE | FALSE | FALSE |
| **ANXA4** | Annexin A4 | 3.76 | 0.702 | 3.06 | 0.00546 | 0.648 | 0.0346 | TRUE | FALSE | TRUE |
| **ANXA5** | Annexin A5 | 1.84 | 0.524 | 1.32 | 0.174 | 0.763 | 0.362 | FALSE | FALSE | FALSE |
| **ANXA6** | Annexin A6 | -1.15 | -0.174 | -0.971 | 0.362 | 0.93 | 0.471 | FALSE | FALSE | FALSE |
| **ANXA7** | Annexin A7 | -0.237 | 0.229 | -0.466 | 0.877 | 0.9 | 0.759 | FALSE | FALSE | FALSE |
| **ANXA8** | Annexin A8 | 1.03 | 0.495 | 0.533 | 0.438 | 0.768 | 0.742 | FALSE | FALSE | FALSE |
| **AP1B1** | AP complex subunit beta | -0.702 | 0.13 | -0.832 | 0.51 | 0.936 | 0.469 | FALSE | FALSE | FALSE |
| **AP1G1** | AP-1 complex subunit gamma | -0.0624 | -0.589 | 0.526 | 0.959 | 0.689 | 0.714 | FALSE | FALSE | FALSE |
| **AP1M2** | AP-1 complex subunit mu-2 | -0.603 | 0.43 | -1.03 | 0.611 | 0.763 | 0.387 | FALSE | FALSE | FALSE |
| **AP2A1** | AP-2 complex subunit alpha | 2.42 | 0.237 | 2.19 | 0.238 | 0.938 | 0.325 | FALSE | FALSE | FALSE |
| **AP2A2** | AP-2 complex subunit alpha-2 | 0.982 | 0.158 | 0.824 | 0.449 | 0.936 | 0.571 | FALSE | FALSE | FALSE |

| | | | | | | | | | | |
|---|---|---|---|---|---|---|---|---|---|---|
| **AP2B1** | AP-2 complex subunit beta | 2.1 | 0.713 | 1.39 | 0.152 | 0.69 | 0.381 | FALSE | FALSE | FALSE |
| **AP2M1** | AP-2 complex subunit mu | 2.63 | 1.06 | 1.57 | 0.0419 | 0.48 | 0.268 | TRUE | FALSE | FALSE |
| **AP3B1** | AP-3 complex subunit beta-1 | -1.41 | -0.371 | -1.04 | 0.246 | 0.822 | 0.423 | FALSE | FALSE | FALSE |
| **AP3D1** | AP-3 complex subunit delta-1 | -0.549 | 0.969 | -1.52 | 0.658 | 0.468 | 0.232 | FALSE | FALSE | FALSE |
| **APBB1IP** | Amyloid beta protein binding family B member 1 interacting protein | 0.00795 | 0.156 | -0.148 | 0.994 | 0.919 | 0.902 | FALSE | FALSE | FALSE |
| **APEX1** | DNA-(apurinic or apyrimidinic site) endonuclease | 0.321 | 0.53 | -0.21 | 0.755 | 0.657 | 0.858 | FALSE | FALSE | FALSE |
| **APMAP** | Adipocyte plasma membrane-associated protein | -0.704 | 0.0856 | -0.79 | 0.468 | 0.955 | 0.445 | FALSE | FALSE | FALSE |
| **APOB** | Apolipoprotein B | -0.451 | -1.69 | 1.24 | 0.838 | 0.431 | 0.561 | FALSE | FALSE | FALSE |
| **APOC3** | Apolipoprotein C-III | 0.654 | 0.674 | -0.0195 | 0.628 | 0.666 | 0.988 | FALSE | FALSE | FALSE |
| **APOD** | Apolipoprotein D | 2.4 | 1.12 | 1.28 | 0.172 | 0.599 | 0.505 | FALSE | FALSE | FALSE |
| **APPL1** | APPL1 protein | -0.396 | 0.291 | -0.687 | 0.744 | 0.858 | 0.594 | FALSE | FALSE | FALSE |
| **APRT** | Adenine phosphoribosyltransferase | 1.11 | -0.963 | 2.07 | 0.382 | 0.512 | 0.144 | FALSE | FALSE | FALSE |
| **ARCN1** | Coatomer subunit delta | 1.37 | 1.27 | 0.106 | 0.204 | 0.326 | 0.933 | FALSE | FALSE | FALSE |
| **ARF1** | ADP-ribosylation factor 1 | -1.02 | -2.7 | 1.68 | 0.623 | 0.265 | 0.423 | FALSE | FALSE | FALSE |
| **ARF4** | ADP-ribosylation factor 4 | 0.964 | -0.318 | 1.28 | 0.508 | 0.873 | 0.401 | FALSE | FALSE | FALSE |

| | | | | | | | | | | |
|---|---|---|---|---|---|---|---|---|---|---|
| **ARF6** | ADP-ribosylation factor 6 | -0.91 | -0.937 | 0.0268 | 0.379 | 0.418 | 0.985 | FALSE | FALSE | FALSE |
| **ARFGEF1** | Brefeldin A-inhibited guanine nucleotide-exchange protein 1 | -1.61 | 0.392 | -2 | 0.314 | 0.862 | 0.246 | FALSE | FALSE | FALSE |
| **ARHGAP1** | Rho GTPase activating protein 1 | -0.777 | 0.266 | -1.04 | 0.498 | 0.866 | 0.386 | FALSE | FALSE | FALSE |
| **ARHGAP25** | Rho GTPase activating protein 25 | 0.157 | 0.766 | -0.609 | 0.903 | 0.602 | 0.667 | FALSE | FALSE | FALSE |
| **ARHGAP26** | Rho GTPase-activating protein 26 | -0.946 | -1.19 | 0.247 | 0.362 | 0.326 | 0.851 | FALSE | FALSE | FALSE |
| **ARHGAP27** | Rho GTPase activating protein 27 | 0.963 | -1.01 | 1.98 | 0.56 | 0.599 | 0.251 | FALSE | FALSE | FALSE |
| **ARHGDIA** | Rho GDP-dissociation inhibitor 1 | 0.492 | -0.755 | 1.25 | 0.744 | 0.664 | 0.412 | FALSE | FALSE | FALSE |
| **ARHGDIB** | Rho GDP-dissociation inhibitor 2 | -2.86 | -2.22 | -0.643 | 0.254 | 0.438 | 0.846 | FALSE | FALSE | FALSE |
| **ARHGEF1** | Rho guanine nucleotide exchange factor 1 | 0.158 | 2.64 | -2.48 | 0.898 | 0.103 | 0.0541 | FALSE | FALSE | FALSE |
| **ARHGEF7** | Rho guanine nucleotide exchange factor 7 | 1.08 | 1.3 | -0.219 | 0.447 | 0.418 | 0.9 | FALSE | FALSE | FALSE |
| **ARID1A** | AT-rich interaction domain 1A | -0.591 | -0.406 | -0.185 | 0.698 | 0.842 | 0.915 | FALSE | FALSE | FALSE |

| | | | | | | | | | | |
|---|---|---|---|---|---|---|---|---|---|---|
| **ARL3** | ADP-ribosylation factor-like protein 3 | -0.119 | 0.602 | -0.721 | 0.915 | 0.658 | 0.561 | FALSE | FALSE | FALSE |
| **ARL8B** | ADP-ribosylation factor-like protein 8B | -0.795 | 0.259 | -1.05 | 0.494 | 0.87 | 0.386 | FALSE | FALSE | FALSE |
| **ARPC1A** | Actin-related protein 2/3 complex subunit 1A | -1.08 | -1.56 | 0.482 | 0.306 | 0.253 | 0.703 | FALSE | FALSE | FALSE |
| **ARPC1B** | Actin-related protein 2/3 complex subunit 1B | -3.45 | -2.14 | -1.3 | 0.0764 | 0.351 | 0.559 | FALSE | FALSE | FALSE |
| **ARPC2** | Actin-related protein 2/3 complex subunit 2 | -3.31 | -1.92 | -1.39 | 0.175 | 0.502 | 0.62 | FALSE | FALSE | FALSE |
| **ARPC3** | Actin-related protein 2/3 complex subunit 3 | -1.85 | -1.49 | -0.362 | 0.236 | 0.4 | 0.856 | FALSE | FALSE | FALSE |
| **ARPC4** | Actin-related protein 2/3 complex subunit 4 | -3.36 | -3 | -0.36 | 0.0606 | 0.232 | 0.873 | FALSE | FALSE | FALSE |
| **ARPC5** | Actin-related protein 2/3 complex subunit 5 | -2.99 | -1.79 | -1.2 | 0.019 | 0.259 | 0.378 | TRUE | FALSE | FALSE |
| **ARRB2** | Beta-arrestin-2 | 0.251 | 0.0383 | 0.212 | 0.865 | 0.981 | 0.886 | FALSE | FALSE | FALSE |
| **ARRDC1** | Arrestin domain containing 1 | 0.963 | -0.541 | 1.5 | 0.67 | 0.858 | 0.522 | FALSE | FALSE | FALSE |
| **ASRGL1** | Isoaspartyl peptidase/L-asparaginase | -0.472 | 0.13 | -0.601 | 0.766 | 0.955 | 0.743 | FALSE | FALSE | FALSE |
| **ASS1** | Argininosuccinate synthase | -1.97 | -0.925 | -1.04 | 0.221 | 0.639 | 0.562 | FALSE | FALSE | FALSE |

| | | | | | | | | | | |
|---|---|---|---|---|---|---|---|---|---|---|
| **ATG3** | Ubiquitin-like-conjugating enzyme ATG3 | 0.695 | 0.729 | -0.0332 | 0.528 | 0.572 | 0.983 | FALSE | FALSE | FALSE |
| **ATIC** | Bifunctional purine biosynthesis protein ATIC | 1.01 | -1.75 | 2.76 | 0.379 | 0.24 | 0.0346 | FALSE | FALSE | TRUE |
| **ATL3** | Atlastin GTPase 3 | 0.0856 | 0.274 | -0.188 | 0.946 | 0.874 | 0.901 | FALSE | FALSE | FALSE |
| **ATP12A** | Sodium/potassium-transporting ATPase subunit alpha | 1.2 | 0.885 | 0.317 | 0.389 | 0.599 | 0.856 | FALSE | FALSE | FALSE |
| **ATP13A3** | ATPase 13A3 | 0.0375 | -1.13 | 1.17 | 0.981 | 0.512 | 0.469 | FALSE | FALSE | FALSE |
| **ATP1A1** | Sodium/potassium-transporting ATPase subunit alpha-1 | 4.44 | -0.0921 | 4.53 | 0.00748 | 0.967 | 0.0175 | TRUE | FALSE | TRUE |
| **ATP1A2** | Sodium/potassium-transporting ATPase subunit alpha-2 | -0.468 | 0.179 | -0.647 | 0.67 | 0.903 | 0.575 | FALSE | FALSE | FALSE |
| **ATP1B1** | Sodium/potassium-transporting ATPase subunit beta | 4.32 | 0.492 | 3.83 | 0.0113 | 0.833 | 0.0444 | TRUE | FALSE | TRUE |
| **ATP2A2** | Calcium-transporting ATPase | -0.754 | -0.464 | -0.291 | 0.503 | 0.741 | 0.841 | FALSE | FALSE | FALSE |
| **ATP2B4** | Plasma membrane calcium-transporting ATPase 4 | -1.41 | -1.38 | -0.0256 | 0.174 | 0.274 | 0.985 | FALSE | FALSE | FALSE |

| | | | | | | | | | | |
|---|---|---|---|---|---|---|---|---|---|---|
| **ATP6AP1** | V-type proton ATPase subunit S1 | -1.44 | -0.993 | -0.45 | 0.352 | 0.589 | 0.821 | FALSE | FALSE | FALSE |
| **ATP6AP2** | Renin receptor | -1.03 | -1.1 | 0.071 | 0.571 | 0.605 | 0.975 | FALSE | FALSE | FALSE |
| **ATP6V1A** | V-type proton ATPase catalytic subunit A | -1.07 | 0.244 | -1.31 | 0.289 | 0.862 | 0.232 | FALSE | FALSE | FALSE |
| **ATP6V1B2** | V-type proton ATPase subunit B brain isoform | 0.318 | 0.599 | -0.281 | 0.756 | 0.61 | 0.824 | FALSE | FALSE | FALSE |
| **ATP8A1** | Phospholipid-transporting ATPase | -0.834 | -1.08 | 0.247 | 0.402 | 0.351 | 0.847 | FALSE | FALSE | FALSE |
| **ATP8B1** | Phospholipid-transporting ATPase | -1.69 | -1.74 | 0.0551 | 0.172 | 0.26 | 0.975 | FALSE | FALSE | FALSE |
| **ATRN** | Attractin | -0.924 | 0.605 | -1.53 | 0.393 | 0.648 | 0.202 | FALSE | FALSE | FALSE |
| **ATXN2L** | Ataxin 2 like | -1.81 | -2.06 | 0.247 | 0.171 | 0.236 | 0.876 | FALSE | FALSE | FALSE |
| **AZU1** | Azurocidin 1 | -9.72 | -7.01 | -2.71 | 0.000282 | 0.0189 | 0.228 | TRUE | TRUE | FALSE |
| **B2M** | Beta-2-microglobulin | 3.31 | 0.0137 | 3.3 | 0.00687 | 0.994 | 0.0177 | TRUE | FALSE | TRUE |
| **B3GNT3** | Hexosyltransferase | 0.796 | -2.56 | 3.35 | 0.76 | 0.366 | 0.222 | FALSE | FALSE | FALSE |
| **B4GALT1** | Beta-1-4-galactosyltransferase 1 | -0.356 | -2.23 | 1.87 | 0.892 | 0.371 | 0.417 | FALSE | FALSE | FALSE |
| **BAIAP2** | Brain-specific angiogenesis inhibitor 1-associated protein 2 | 5.29 | 0.736 | 4.56 | 0.00839 | 0.765 | 0.039 | TRUE | FALSE | TRUE |

| | | | | | | | | | | |
|---|---|---|---|---|---|---|---|---|---|---|
| **BAIAP2L1** | BAR/IMD domain containing adaptor protein 2 like 1 | 4.98 | 2.24 | 2.73 | 0.0135 | 0.339 | 0.22 | TRUE | FALSE | FALSE |
| **BASP1** | Brain acid soluble protein 1 | 2.01 | 0.655 | 1.35 | 0.177 | 0.727 | 0.396 | FALSE | FALSE | FALSE |
| **BCAM** | Basal cell adhesion molecule | 0.158 | 1.11 | -0.955 | 0.898 | 0.388 | 0.423 | FALSE | FALSE | FALSE |
| **BCAS1** | Brain enriched myelin associated protein 1 | 0.771 | 1.62 | -0.849 | 0.576 | 0.31 | 0.57 | FALSE | FALSE | FALSE |
| **BCAT1** | Branched-chain-amino-acid aminotransferase | 0.158 | 1.19 | -1.03 | 0.892 | 0.326 | 0.344 | FALSE | FALSE | FALSE |
| **BGN** | Biglycan | 0.639 | -0.252 | 0.891 | 0.562 | 0.866 | 0.432 | FALSE | FALSE | FALSE |
| **BIN2** | Bridging integrator 2 | -0.559 | 0.792 | -1.35 | 0.676 | 0.605 | 0.322 | FALSE | FALSE | FALSE |
| **BLVRB** | Flavin reductase (NADPH) | 0.227 | -2.14 | 2.37 | 0.917 | 0.393 | 0.322 | FALSE | FALSE | FALSE |
| **BoLA** | Major histocompatibility complex class I | 0.837 | -0.275 | 1.11 | 0.487 | 0.867 | 0.381 | FALSE | FALSE | FALSE |
| **BOLA** | Ig-like domain-containing protein | -1.29 | -0.563 | -0.728 | 0.51 | 0.835 | 0.759 | FALSE | FALSE | FALSE |
| **BoLA-DQB** | BoLa class II histocompatibility antigen DQB*0101 beta chain | -0.559 | -2.18 | 1.62 | 0.773 | 0.316 | 0.404 | FALSE | FALSE | FALSE |
| **BoLA-DRA** | BoLA-DR-alpha | 1.56 | -2.72 | 4.28 | 0.352 | 0.232 | 0.0254 | FALSE | FALSE | TRUE |
| **BOLA-DRB2** | Ig-like domain-containing protein | -0.984 | -0.776 | -0.208 | 0.434 | 0.61 | 0.893 | FALSE | FALSE | FALSE |

| | | | | | | | | | | |
|---|---|---|---|---|---|---|---|---|---|---|
| **BOLA-DRB3** | Ig-like domain-containing protein | -0.43 | 0.75 | -1.18 | 0.755 | 0.637 | 0.39 | FALSE | FALSE | FALSE |
| **BOLA-NC1** | Non-classical MHC class I antigen | -0.721 | -0.36 | -0.361 | 0.621 | 0.854 | 0.841 | FALSE | FALSE | FALSE |
| **BPI** | Bactericidal permeability-increasing protein | -1.76 | 1.3 | -3.06 | 0.272 | 0.484 | 0.0873 | FALSE | FALSE | FALSE |
| **BPIFB1** | BPI fold-containing family B member 1 | 9.11 | 1.77 | 7.33 | 9.68E-05 | 0.344 | 0.00124 | TRUE | FALSE | TRUE |
| **BPNT1** | 3(2)5-bisphosphate nucleotidase 1 | -0.874 | -1.36 | 0.483 | 0.426 | 0.299 | 0.716 | FALSE | FALSE | FALSE |
| **BROX** | BRO1 domain-containing protein BROX | -0.833 | -0.481 | -0.352 | 0.394 | 0.69 | 0.773 | FALSE | FALSE | FALSE |
| **BSPRY** | B-box and SPRY domain containing | -1.7 | -0.373 | -1.33 | 0.246 | 0.858 | 0.395 | FALSE | FALSE | FALSE |
| **BUB3** | Mitotic checkpoint protein BUB3 | -0.825 | 0.593 | -1.42 | 0.503 | 0.69 | 0.277 | FALSE | FALSE | FALSE |
| **C1QA** | Complement C1q subcomponent subunit A | -1.36 | -0.852 | -0.504 | 0.247 | 0.538 | 0.723 | FALSE | FALSE | FALSE |
| **C1QB** | Complement C1q subcomponent subunit B | 0.51 | 0.123 | 0.386 | 0.809 | 0.964 | 0.867 | FALSE | FALSE | FALSE |
| **C1QC** | Adiponectin B | -0.879 | -0.362 | -0.517 | 0.633 | 0.883 | 0.821 | FALSE | FALSE | FALSE |
| **C1R** | Complement subcomponent C1r | -0.428 | -3.7 | 3.27 | 0.901 | 0.334 | 0.344 | FALSE | FALSE | FALSE |
| **C1S** | Complement C1s subcomponent | -2.68 | -4.77 | 2.09 | 0.277 | 0.186 | 0.428 | FALSE | FALSE | FALSE |
| **C23H6orf132** | Chromosome 23 C6orf132 homolog | 1.92 | 0.901 | 1.02 | 0.177 | 0.605 | 0.521 | FALSE | FALSE | FALSE |

| | | | | | | | | | | |
|---|---|---|---|---|---|---|---|---|---|---|
| **C6** | Complement component C6 | -2.06 | 0.0836 | -2.15 | 0.174 | 0.967 | 0.206 | FALSE | FALSE | FALSE |
| **C8B** | Complement component C8 beta chain | -1.26 | 0.429 | -1.69 | 0.239 | 0.754 | 0.157 | FALSE | FALSE | FALSE |
| **C8G** | Uncharacterized protein | 0.212 | 0.813 | -0.601 | 0.912 | 0.729 | 0.803 | FALSE | FALSE | FALSE |
| **CA4** | Carbonic anhydrase 4 | 0.938 | 1.11 | -0.169 | 0.434 | 0.416 | 0.903 | FALSE | FALSE | FALSE |
| **CAB39** | Calcium-binding protein 39 | -0.461 | -1.75 | 1.29 | 0.695 | 0.236 | 0.282 | FALSE | FALSE | FALSE |
| **CACNA2D1** | Calcium voltage-gated channel auxiliary subunit alpha2delta 1 | 0.508 | -0.588 | 1.1 | 0.743 | 0.752 | 0.489 | FALSE | FALSE | FALSE |
| **CAD** | CAD protein | 0.574 | 1.71 | -1.13 | 0.641 | 0.256 | 0.359 | FALSE | FALSE | FALSE |
| **CALM** | Calmodulin | 2.41 | -0.122 | 2.53 | 0.0493 | 0.95 | 0.0726 | TRUE | FALSE | FALSE |
| **CALR** | Calreticulin | 4.47 | 1.52 | 2.95 | 0.00947 | 0.42 | 0.115 | TRUE | FALSE | FALSE |
| **CAMK2B** | Calcium/calmodulin-dependent protein kinase | -0.642 | -0.00117 | -0.641 | 0.63 | 0.999 | 0.663 | FALSE | FALSE | FALSE |
| **CAMK2D** | Calcium/calmodulin-dependent protein kinase type II subunit delta | 0.783 | 2.37 | -1.59 | 0.654 | 0.256 | 0.36 | FALSE | FALSE | FALSE |
| **CAND1** | Cullin-associated NEDD8-dissociated protein 1 | -0.0647 | -0.857 | 0.792 | 0.952 | 0.469 | 0.476 | FALSE | FALSE | FALSE |
| **CANX** | Calnexin | 2.16 | 0.0602 | 2.1 | 0.184 | 0.978 | 0.239 | FALSE | FALSE | FALSE |

| | | | | | | | | | | |
|---|---|---|---|---|---|---|---|---|---|---|
| **CAP1** | Adenylyl cyclase-associated protein 1 | -1.73 | -1.63 | -0.096 | 0.115 | 0.256 | 0.942 | FALSE | FALSE | FALSE |
| **CAPG** | Macrophage-capping protein | -1.06 | -3.18 | 2.12 | 0.539 | 0.186 | 0.239 | FALSE | FALSE | FALSE |
| **CAPN1** | Calpain-1 catalytic subunit | 1.84 | -0.135 | 1.97 | 0.257 | 0.955 | 0.267 | FALSE | FALSE | FALSE |
| **CAPN2** | Calpain-2 catalytic subunit | -2.18 | -1.79 | -0.395 | 0.2 | 0.363 | 0.856 | FALSE | FALSE | FALSE |
| **CAPN5** | Calpain 5 | 3.41 | 0.218 | 3.19 | 0.00724 | 0.901 | 0.0244 | TRUE | FALSE | TRUE |
| **CAPNS1** | Calpain small subunit 1 | 1.23 | -0.53 | 1.76 | 0.413 | 0.788 | 0.277 | FALSE | FALSE | FALSE |
| **CAPRIN1** | Caprin-1 | -0.837 | 0.345 | -1.18 | 0.488 | 0.835 | 0.352 | FALSE | FALSE | FALSE |
| **CAPS** | Calcyphosin | -0.409 | 1.6 | -2.01 | 0.777 | 0.324 | 0.185 | FALSE | FALSE | FALSE |
| **CAPZA1** | F-actin-capping protein subunit alpha-1 | -1.55 | -1.27 | -0.279 | 0.152 | 0.326 | 0.845 | FALSE | FALSE | FALSE |
| **CAPZA2** | F-actin-capping protein subunit alpha | -0.378 | -0.668 | 0.29 | 0.74 | 0.606 | 0.838 | FALSE | FALSE | FALSE |
| **CAPZB** | F-actin-capping protein subunit beta | -0.932 | -0.569 | -0.362 | 0.402 | 0.676 | 0.799 | FALSE | FALSE | FALSE |
| **CARMIL1** | Capping protein regulator and myosin 1 linker 1 | -1.21 | 2 | -3.2 | 0.474 | 0.317 | 0.0869 | FALSE | FALSE | FALSE |
| **CASP6** | Caspase-6 | -0.943 | 0.85 | -1.79 | 0.581 | 0.673 | 0.312 | FALSE | FALSE | FALSE |
| **CASP8** | Caspase 8 | -2.07 | -0.00846 | -2.06 | 0.149 | 0.996 | 0.197 | FALSE | FALSE | FALSE |
| **CAT** | Catalase | 0.158 | 2.38 | -2.22 | 0.94 | 0.337 | 0.327 | FALSE | FALSE | FALSE |
| **CATHL1** | Cathelicidin-1 | -8.41 | -7.01 | -1.4 | 9.68E-05 | 0.00244 | 0.381 | TRUE | TRUE | FALSE |
| **CATHL1.1** | Cathelicidin-1 | -6.53 | -5.25 | -1.28 | 4.91E-05 | 0.00125 | 0.229 | TRUE | TRUE | FALSE |

| | | | | | | | | | | |
|---|---|---|---|---|---|---|---|---|---|---|
| **CATHL2** | Cathelicidin-2 | -2.85 | -1.19 | -1.66 | 0.0115 | 0.356 | 0.179 | TRUE | FALSE | FALSE |
| **CATHL3** | Cathelicidin-3 | 0.158 | 0.33 | -0.171 | 0.889 | 0.784 | 0.876 | FALSE | FALSE | FALSE |
| **CATHL4** | Cathelicidin-4 | -4.27 | -2.94 | -1.33 | 0.0013 | 0.0756 | 0.277 | TRUE | FALSE | FALSE |
| **CATHL5** | Cathelicidin-5 | -0.68 | -0.65 | -0.0293 | 0.63 | 0.69 | 0.985 | FALSE | FALSE | FALSE |
| **CATHL6** | Cathelicidin-6 | -1.31 | 0.0374 | -1.34 | 0.186 | 0.978 | 0.226 | FALSE | FALSE | FALSE |
| **CATHL7** | Cathelicidin-7 | -6.82 | -6.42 | -0.409 | 4.91E-05 | 0.000602 | 0.744 | TRUE | TRUE | FALSE |
| **CCAR1** | Cell division cycle and apoptosis regulator 1 | -0.759 | -0.411 | -0.349 | 0.503 | 0.775 | 0.812 | FALSE | FALSE | FALSE |
| **CCAR2** | Cell cycle and apoptosis regulator 2 | -0.963 | 0.17 | -1.13 | 0.521 | 0.938 | 0.475 | FALSE | FALSE | FALSE |
| **CCDC186** | Coiled-coil domain containing 186 | 1.81 | 2.68 | -0.866 | 0.195 | 0.186 | 0.584 | FALSE | FALSE | FALSE |
| **CCDC6** | Coiled-coil domain containing 6 | -0.702 | -0.0735 | -0.628 | 0.526 | 0.962 | 0.613 | FALSE | FALSE | FALSE |
| **CCM2** | CCM2 scaffold protein | -1.41 | -0.0807 | -1.33 | 0.562 | 0.978 | 0.62 | FALSE | FALSE | FALSE |
| **CCNY** | CCNY protein | 0.158 | 2.02 | -1.86 | 0.905 | 0.236 | 0.197 | FALSE | FALSE | FALSE |
| **CCT2** | T-complex protein 1 subunit beta | 0.355 | -0.464 | 0.819 | 0.782 | 0.762 | 0.543 | FALSE | FALSE | FALSE |
| **CCT3** | T-complex protein 1 subunit gamma | 0.993 | -0.36 | 1.35 | 0.488 | 0.858 | 0.366 | FALSE | FALSE | FALSE |
| **CCT4** | T-complex protein 1 subunit delta | -0.722 | -0.936 | 0.214 | 0.731 | 0.692 | 0.924 | FALSE | FALSE | FALSE |
| **CCT5** | T-complex protein 1 subunit epsilon | 0.0309 | -1.31 | 1.34 | 0.987 | 0.487 | 0.444 | FALSE | FALSE | FALSE |
| **CCT6A** | T-complex protein 1 subunit zeta | 0.833 | -0.187 | 1.02 | 0.461 | 0.913 | 0.395 | FALSE | FALSE | FALSE |
| **CCT7** | T-complex protein 1 subunit eta | 1.94 | -0.078 | 2.01 | 0.144 | 0.967 | 0.177 | FALSE | FALSE | FALSE |

| | | | | | | | | | | |
|---|---|---|---|---|---|---|---|---|---|---|
| **CCT8** | T-complex protein 1 subunit theta | -0.511 | -1.61 | 1.1 | 0.75 | 0.357 | 0.501 | FALSE | FALSE | FALSE |
| **CD109** | CD109 molecule | 4.5 | 2.13 | 2.37 | 0.0517 | 0.418 | 0.344 | FALSE | FALSE | FALSE |
| **CD14** | Monocyte differentiation antigen CD14 | 2.07 | -0.616 | 2.69 | 0.0566 | 0.648 | 0.0346 | FALSE | FALSE | TRUE |
| **CD177** | CD177 molecule | -9.97 | -5.27 | -4.7 | 0.000411 | 0.103 | 0.0669 | TRUE | FALSE | FALSE |
| **CD2AP** | CD2 associated protein | -0.213 | -0.18 | -0.0339 | 0.881 | 0.918 | 0.984 | FALSE | FALSE | FALSE |
| **CD44** | CD44 antigen | 0.295 | -1.03 | 1.33 | 0.865 | 0.538 | 0.385 | FALSE | FALSE | FALSE |
| **CD46** | Membrane cofactor protein | 1.78 | 0.497 | 1.29 | 0.103 | 0.718 | 0.282 | FALSE | FALSE | FALSE |
| **CD47** | Leukocyte surface antigen CD47 | 0.875 | -1.09 | 1.97 | 0.668 | 0.642 | 0.336 | FALSE | FALSE | FALSE |
| **CD48** | CD48 molecule | -0.709 | -1.36 | 0.647 | 0.56 | 0.328 | 0.632 | FALSE | FALSE | FALSE |
| **CD55** | Uncharacterized protein | 1.18 | -0.744 | 1.92 | 0.628 | 0.807 | 0.433 | FALSE | FALSE | FALSE |
| **CD58** | CD58 molecule | 0.454 | 0.569 | -0.114 | 0.67 | 0.644 | 0.921 | FALSE | FALSE | FALSE |
| **CD59** | CD59 glycoprotein | 0.611 | -1.79 | 2.41 | 0.691 | 0.31 | 0.144 | FALSE | FALSE | FALSE |
| **CD5L** | CD5 molecule like | 1.03 | 0.72 | 0.312 | 0.647 | 0.799 | 0.901 | FALSE | FALSE | FALSE |
| **CD63** | CD63 antigen | 0.692 | 0.468 | 0.225 | 0.738 | 0.862 | 0.921 | FALSE | FALSE | FALSE |
| **CD82** | Tetraspanin | -0.437 | 0.219 | -0.656 | 0.704 | 0.886 | 0.595 | FALSE | FALSE | FALSE |
| **CD9** | CD9 antigen | 0.386 | -1.63 | 2.02 | 0.767 | 0.275 | 0.144 | FALSE | FALSE | FALSE |
| **CD99** | CD99 molecule | -0.372 | -0.274 | -0.0973 | 0.73 | 0.848 | 0.933 | FALSE | FALSE | FALSE |
| **CDA** | Cytidine deaminase | -0.761 | -0.136 | -0.625 | 0.58 | 0.947 | 0.686 | FALSE | FALSE | FALSE |
| **CDC37** | Hsp90 co-chaperone Cdc37 | 0.158 | 1.1 | -0.938 | 0.898 | 0.393 | 0.43 | FALSE | FALSE | FALSE |
| **CDC42** | Cell division control protein 42 homolog | -0.153 | -0.979 | 0.826 | 0.895 | 0.408 | 0.469 | FALSE | FALSE | FALSE |
| **CDH1** | Cadherin-1 | -0.919 | -0.702 | -0.217 | 0.415 | 0.606 | 0.875 | FALSE | FALSE | FALSE |
| **CDH17** | Cadherin 17 | 1.45 | -3.73 | 5.18 | 0.447 | 0.186 | 0.0223 | FALSE | FALSE | TRUE |

| | | | | | | | | | | |
|---|---|---|---|---|---|---|---|---|---|---|
| **CDK16** | Cyclin dependent kinase 16 | -2.59 | -2.33 | -0.262 | 0.102 | 0.256 | 0.894 | FALSE | FALSE | FALSE |
| **CDSN** | Corneodesmosin | -0.961 | -1.21 | 0.247 | 0.404 | 0.363 | 0.858 | FALSE | FALSE | FALSE |
| **CEACAM1** | Uncharacterized protein | 0.322 | -1.26 | 1.59 | 0.809 | 0.363 | 0.229 | FALSE | FALSE | FALSE |
| **CENPV** | Centromere protein V | -0.824 | -0.401 | -0.423 | 0.485 | 0.794 | 0.769 | FALSE | FALSE | FALSE |
| **CFDP2** | Craniofacial development protein 2 | 5.41 | 2.02 | 3.39 | 0.00499 | 0.335 | 0.0873 | TRUE | FALSE | FALSE |
| **CFDP2.1** | Craniofacial development protein 2 | 0.256 | 0.914 | -0.658 | 0.876 | 0.565 | 0.674 | FALSE | FALSE | FALSE |
| **CFL1** | Cofilin-1 | -1.95 | -1.98 | 0.0322 | 0.184 | 0.271 | 0.985 | FALSE | FALSE | FALSE |
| **CFP** | Complement factor properdin | 0.257 | -0.0885 | 0.346 | 0.789 | 0.95 | 0.75 | FALSE | FALSE | FALSE |
| **CGN1** | Conglutinin | -1.65 | -0.52 | -1.13 | 0.404 | 0.852 | 0.619 | FALSE | FALSE | FALSE |
| **CHI3L1** | Chitinase-3-like protein 1 | -0.349 | 3.2 | -3.55 | 0.875 | 0.214 | 0.0808 | FALSE | FALSE | FALSE |
| **CHMP1A** | Charged multivesicular body protein 1a | -1.32 | 0.222 | -1.54 | 0.352 | 0.917 | 0.306 | FALSE | FALSE | FALSE |
| **CHMP1B** | Charged multivesicular body protein 1b | -0.237 | 1.2 | -1.44 | 0.826 | 0.285 | 0.174 | FALSE | FALSE | FALSE |
| **CHMP2A** | Charged multivesicular body protein 2A | 0.00161 | 1.3 | -1.29 | 0.999 | 0.265 | 0.222 | FALSE | FALSE | FALSE |
| **CHMP4B** | CHMP4B protein | -1.15 | -1.05 | -0.102 | 0.274 | 0.388 | 0.933 | FALSE | FALSE | FALSE |

| | | | | | | | | | | |
|---|---|---|---|---|---|---|---|---|---|---|
| **CIB1** | Calcium and integrin-binding protein 1 | 0.00706 | 0.233 | -0.226 | 0.997 | 0.915 | 0.898 | FALSE | FALSE | FALSE |
| **CKAP4** | Cytoskeleton associated protein 4 | -1.3 | -0.93 | -0.367 | 0.229 | 0.446 | 0.788 | FALSE | FALSE | FALSE |
| **CKAP5** | Cytoskeleton associated protein 5 | -1.83 | -2.08 | 0.247 | 0.152 | 0.236 | 0.875 | FALSE | FALSE | FALSE |
| **CKB** | Creatine kinase B-type | 2.3 | -0.388 | 2.69 | 0.022 | 0.763 | 0.0228 | TRUE | FALSE | TRUE |
| **CLCA1** | Chloride channel accessory 1 | 6.66 | 0.283 | 6.37 | 0.00491 | 0.933 | 0.0154 | TRUE | FALSE | TRUE |
| **CLDN3** | Claudin-3 | 1.09 | 0.106 | 0.989 | 0.389 | 0.955 | 0.471 | FALSE | FALSE | FALSE |
| **CLIC1** | Chloride intracellular channel protein 1 | 0.671 | -0.851 | 1.52 | 0.609 | 0.565 | 0.257 | FALSE | FALSE | FALSE |
| **CLIC4** | Chloride intracellular channel protein 4 | 1.21 | -1.18 | 2.4 | 0.362 | 0.425 | 0.1 | FALSE | FALSE | FALSE |
| **CLIC5** | Chloride intracellular channel protein 5 | -0.173 | 0.987 | -1.16 | 0.892 | 0.434 | 0.332 | FALSE | FALSE | FALSE |
| **CLIC6** | Chloride intracellular channel protein | 0.168 | 0.839 | -0.671 | 0.89 | 0.46 | 0.549 | FALSE | FALSE | FALSE |
| **CLINT1** | Clathrin interactor 1 | -1.63 | -1.14 | -0.487 | 0.249 | 0.485 | 0.787 | FALSE | FALSE | FALSE |
| **CLTA** | Clathrin light chain | 5.13 | 1.83 | 3.3 | 0.00479 | 0.344 | 0.0797 | TRUE | FALSE | FALSE |
| **CLTB** | Clathrin light chain | 3.04 | 2.16 | 0.878 | 0.0644 | 0.284 | 0.654 | FALSE | FALSE | FALSE |
| **CLTC** | Clathrin heavy chain 1 | 2.54 | 0.463 | 2.08 | 0.0499 | 0.788 | 0.154 | TRUE | FALSE | FALSE |

| | | | | | | | | | | |
|---|---|---|---|---|---|---|---|---|---|---|
| **CMAS** | N-acylneuraminate cytidylyltransferase | -0.763 | -0.165 | -0.598 | 0.463 | 0.917 | 0.617 | FALSE | FALSE | FALSE |
| **CMBL** | Carboxymethylenebutenolidase homolog | -0.847 | -0.0949 | -0.752 | 0.377 | 0.95 | 0.469 | FALSE | FALSE | FALSE |
| **CMTM7** | CKLF-like MARVEL transmembrane domain containing 7 | -0.594 | 0.13 | -0.723 | 0.578 | 0.935 | 0.524 | FALSE | FALSE | FALSE |
| **CNBP** | CCHC-type zinc finger nucleic acid binding protein | 0.369 | 2.27 | -1.9 | 0.829 | 0.244 | 0.239 | FALSE | FALSE | FALSE |
| **CNDP2** | Cytosolic non-specific dipeptidase | 0.252 | -0.994 | 1.25 | 0.886 | 0.561 | 0.415 | FALSE | FALSE | FALSE |
| **CNN2** | Calponin-2 | -0.543 | -1.3 | 0.752 | 0.719 | 0.421 | 0.654 | FALSE | FALSE | FALSE |
| **CNN3** | Calponin-3 | -1.26 | -1.51 | 0.247 | 0.249 | 0.265 | 0.857 | FALSE | FALSE | FALSE |
| **CNOT1** | CCR4-NOT transcription complex subunit 1 | -1.02 | -0.804 | -0.215 | 0.371 | 0.543 | 0.875 | FALSE | FALSE | FALSE |
| **CNP** | 2'3-cyclic-nucleotide 3-phosphodiesterase | 1.49 | -1.14 | 2.63 | 0.298 | 0.487 | 0.0938 | FALSE | FALSE | FALSE |
| **CNTLN** | Centlein | 0.0463 | 0.403 | -0.357 | 0.97 | 0.805 | 0.824 | FALSE | FALSE | FALSE |
| **COL15A1** | Collagen type XV alpha 1 chain | -0.705 | 0.501 | -1.21 | 0.609 | 0.763 | 0.385 | FALSE | FALSE | FALSE |
| **COL1A1** | Collagen alpha-1(I) chain | -0.0142 | 0.601 | -0.615 | 0.992 | 0.689 | 0.663 | FALSE | FALSE | FALSE |
| **COL1A2** | Collagen alpha-2(I) chain | -1.14 | -0.374 | -0.763 | 0.486 | 0.867 | 0.684 | FALSE | FALSE | FALSE |
| **COL4A1** | Collagen type IV alpha 1 chain | 0.696 | 1.09 | -0.393 | 0.615 | 0.472 | 0.82 | FALSE | FALSE | FALSE |

| | | | | | | | | | | |
|---|---|---|---|---|---|---|---|---|---|---|
| **COL6A1** | Collagen type VI alpha 1 chain | -1.02 | -1.25 | 0.233 | 0.415 | 0.387 | 0.876 | FALSE | FALSE | FALSE |
| **COL6A2** | Collagen type VI alpha 2 chain | 0.695 | 1.31 | -0.613 | 0.689 | 0.493 | 0.763 | FALSE | FALSE | FALSE |
| **COL6A3** | Collagen type VI alpha 3 chain | -1.92 | 0.522 | -2.45 | 0.306 | 0.842 | 0.232 | FALSE | FALSE | FALSE |
| **COPA** | Coatomer subunit alpha | 7.4 | 1.37 | 6.03 | 9.05E-05 | 0.342 | 0.000811 | TRUE | FALSE | TRUE |
| **COPB1** | Coatomer subunit beta | 3.08 | 1.42 | 1.66 | 0.152 | 0.587 | 0.476 | FALSE | FALSE | FALSE |
| **COPB2** | Coatomer subunit beta | 4.47 | 1.29 | 3.18 | 0.00207 | 0.381 | 0.0312 | TRUE | FALSE | TRUE |
| **COPE** | Coatomer subunit epsilon | 0.216 | 0.561 | -0.346 | 0.838 | 0.608 | 0.755 | FALSE | FALSE | FALSE |
| **COPG1** | Coatomer subunit gamma | 3.05 | 1.01 | 2.04 | 0.04 | 0.574 | 0.222 | TRUE | FALSE | FALSE |
| **COPG2** | Coatomer subunit gamma | -0.814 | -1.06 | 0.247 | 0.42 | 0.363 | 0.848 | FALSE | FALSE | FALSE |
| **CORO1A** | Coronin-1A | -6.88 | -6.04 | -0.843 | 0.0205 | 0.172 | 0.825 | TRUE | FALSE | FALSE |
| **CORO1B** | Coronin | -0.0607 | -0.295 | 0.234 | 0.956 | 0.842 | 0.857 | FALSE | FALSE | FALSE |
| **CORO2A** | Coronin-2A | -2.04 | -1.38 | -0.66 | 0.0904 | 0.339 | 0.647 | FALSE | FALSE | FALSE |
| **COTL1** | Coactosin-like protein | -1.4 | -1.15 | -0.247 | 0.174 | 0.342 | 0.853 | FALSE | FALSE | FALSE |
| **CP** | Ceruloplasmin | -2.25 | -0.91 | -1.34 | 0.0629 | 0.526 | 0.315 | FALSE | FALSE | FALSE |
| **CPD** | Carboxypeptidase D | -0.323 | -2.36 | 2.04 | 0.881 | 0.265 | 0.277 | FALSE | FALSE | FALSE |
| **CPNE1** | Copine-1 | -0.163 | -0.736 | 0.573 | 0.892 | 0.553 | 0.636 | FALSE | FALSE | FALSE |
| **CPNE2** | Copine 2 | -0.745 | 0.984 | -1.73 | 0.596 | 0.526 | 0.233 | FALSE | FALSE | FALSE |
| **CPNE3** | CPNE3 protein | -1.85 | -0.309 | -1.54 | 0.0787 | 0.835 | 0.194 | FALSE | FALSE | FALSE |
| **CRIP1** | Cysteine-rich protein 1 | 0.835 | 0.525 | 0.309 | 0.377 | 0.647 | 0.796 | FALSE | FALSE | FALSE |

| | | | | | | | | | | |
|---|---|---|---|---|---|---|---|---|---|---|
| **CRIP2** | Cysteine-rich protein 2 | 0.558 | -0.0216 | 0.58 | 0.666 | 0.992 | 0.684 | FALSE | FALSE | FALSE |
| **CRISP3** | Cysteine-rich secretory protein 2 | 0.469 | -0.0885 | 0.558 | 0.63 | 0.952 | 0.592 | FALSE | FALSE | FALSE |
| **CRYBG1** | Crystallin beta-gamma domain containing 1 | -0.542 | 0.904 | -1.45 | 0.789 | 0.69 | 0.475 | FALSE | FALSE | FALSE |
| **CSDE1** | Cold shock domain containing E1 | -0.973 | 0.0261 | -0.999 | 0.394 | 0.988 | 0.412 | FALSE | FALSE | FALSE |
| **CSE1L** | Exportin-2 | -2.74 | 0.615 | -3.36 | 0.421 | 0.901 | 0.352 | FALSE | FALSE | FALSE |
| **CSK** | Tyrosine-protein kinase | -1.39 | -2.02 | 0.628 | 0.172 | 0.186 | 0.585 | FALSE | FALSE | FALSE |
| **CSNK2A1** | Casein kinase II subunit alpha | -2.13 | -0.445 | -1.68 | 0.184 | 0.846 | 0.331 | FALSE | FALSE | FALSE |
| **CSNK2B** | Casein kinase II subunit beta | -0.663 | -0.168 | -0.494 | 0.607 | 0.931 | 0.738 | FALSE | FALSE | FALSE |
| **CSRP1** | Cysteine and glycine-rich protein 1 | -0.181 | 0.322 | -0.502 | 0.885 | 0.812 | 0.668 | FALSE | FALSE | FALSE |
| **CST6** | Cystatin E/M | -1 | -0.925 | -0.0761 | 0.562 | 0.65 | 0.975 | FALSE | FALSE | FALSE |
| **CSTB** | Cystatin-B | 2.62 | 0.281 | 2.34 | 0.0275 | 0.867 | 0.0808 | TRUE | FALSE | FALSE |
| **CTBP1** | C-terminal binding protein 1 | -0.983 | -0.254 | -0.729 | 0.39 | 0.873 | 0.571 | FALSE | FALSE | FALSE |
| **CTNNA1** | Catenin alpha-1 | 5.34 | 2.28 | 3.06 | 0.00556 | 0.285 | 0.128 | TRUE | FALSE | FALSE |
| **CTNNB1** | Catenin beta-1 | -0.26 | 0.583 | -0.843 | 0.835 | 0.659 | 0.469 | FALSE | FALSE | FALSE |
| **CTNND1** | Catenin delta 1 | 1.68 | 1.42 | 0.264 | 0.139 | 0.301 | 0.856 | FALSE | FALSE | FALSE |
| **CTSA** | Carboxypeptidase | 0.158 | 1.61 | -1.45 | 0.912 | 0.333 | 0.332 | FALSE | FALSE | FALSE |
| **CTSB** | Cathepsin B | -0.31 | -0.175 | -0.135 | 0.784 | 0.913 | 0.915 | FALSE | FALSE | FALSE |
| **CTSD** | Cathepsin D | -0.554 | -2.54 | 1.98 | 0.789 | 0.276 | 0.336 | FALSE | FALSE | FALSE |
| **CTSH** | Pro-cathepsin H | 0.158 | 0.476 | -0.317 | 0.915 | 0.804 | 0.857 | FALSE | FALSE | FALSE |
| **CTSZ** | Cathepsin Z | -0.519 | -1.37 | 0.848 | 0.74 | 0.416 | 0.618 | FALSE | FALSE | FALSE |

| | | | | | | | | | | |
|---|---|---|---|---|---|---|---|---|---|---|
| **CUL3** | Cullin 3 | -1.61 | -1.32 | -0.294 | 0.229 | 0.393 | 0.857 | FALSE | FALSE | FALSE |
| **CXADR** | Coxsackievirus and adenovirus receptor homolog | 0.158 | 1.16 | -1 | 0.892 | 0.332 | 0.351 | FALSE | FALSE | FALSE |
| **CXCL17** | C-X-C motif chemokine 17 | 1.15 | 0.9 | 0.247 | 0.287 | 0.465 | 0.856 | FALSE | FALSE | FALSE |
| **CYB5B** | Cytochrome b5 type B | 0.955 | 0.37 | 0.585 | 0.344 | 0.768 | 0.605 | FALSE | FALSE | FALSE |
| **CYB5R3** | NADH-cytochrome b5 reductase | 0.664 | 1.02 | -0.358 | 0.613 | 0.48 | 0.825 | FALSE | FALSE | FALSE |
| **CYBB** | XK-related protein | -1.37 | -1.55 | 0.183 | 0.434 | 0.431 | 0.926 | FALSE | FALSE | FALSE |
| **CYBRD1** | Plasma membrane ascorbate-dependent reductase CYBRD1 | -1.65 | -1.59 | -0.058 | 0.247 | 0.342 | 0.975 | FALSE | FALSE | FALSE |
| **CYFIP1** | Cytoplasmic FMR1-interacting protein | 1.54 | -0.181 | 1.72 | 0.265 | 0.933 | 0.253 | FALSE | FALSE | FALSE |
| **CYFIP2** | Cytoplasmic FMR1-interacting protein | 0.188 | 0.263 | -0.0749 | 0.892 | 0.867 | 0.961 | FALSE | FALSE | FALSE |
| **CYP39A1** | CYP39A1 protein | -1.81 | -2.2 | 0.397 | 0.196 | 0.236 | 0.827 | FALSE | FALSE | FALSE |
| **CYRIB** | CYFIP-related Rac1 interactor B | -1.65 | -1.61 | -0.0452 | 0.338 | 0.403 | 0.985 | FALSE | FALSE | FALSE |
| **CYSTM1** | Cysteine-rich and transmembrane domain-containing protein 1 | -0.489 | 0.0506 | -0.539 | 0.61 | 0.967 | 0.602 | FALSE | FALSE | FALSE |
| **DAG1** | Dystroglycan 1 | -1.56 | -1.33 | -0.231 | 0.194 | 0.344 | 0.875 | FALSE | FALSE | FALSE |
| **DARS1** | Aspartate-tRNA ligase cytoplasmic | 3.94 | 1.27 | 2.68 | 0.0314 | 0.565 | 0.193 | TRUE | FALSE | FALSE |
| **DBNL** | Drebrin-like protein | 0.831 | -1.82 | 2.65 | 0.676 | 0.393 | 0.2 | FALSE | FALSE | FALSE |

| | | | | | | | | | | |
|---|---|---|---|---|---|---|---|---|---|---|
| **DCDC2** | Doublecortin domain containing 2 | -2.34 | -2.59 | 0.247 | 0.0149 | 0.0635 | 0.843 | TRUE | FALSE | FALSE |
| **DCTN1** | Dynactin subunit 1 | 0.809 | -0.394 | 1.2 | 0.715 | 0.896 | 0.619 | FALSE | FALSE | FALSE |
| **DCTN2** | Dynactin subunit 2 | 2.08 | 0.804 | 1.27 | 0.12 | 0.629 | 0.381 | FALSE | FALSE | FALSE |
| **DDAH1** | N(G)N(G)-dimethylarginine dimethylaminohydrolase 1 | -0.11 | -0.00459 | -0.105 | 0.915 | 0.996 | 0.928 | FALSE | FALSE | FALSE |
| **DDAH2** | N(G)N(G)-dimethylarginine dimethylaminohydrolase 2 | -0.734 | 1.9 | -2.63 | 0.751 | 0.444 | 0.268 | FALSE | FALSE | FALSE |
| **DDB1** | DNA damage-binding protein 1 | -1.06 | 0.568 | -1.63 | 0.422 | 0.733 | 0.253 | FALSE | FALSE | FALSE |
| **DDOST** | Dolichyl-diphosphooligosaccharide--protein glycosyltransferase 48 kDa subunit | -1.45 | 0.131 | -1.58 | 0.302 | 0.952 | 0.294 | FALSE | FALSE | FALSE |
| **DDX1** | ATP-dependent RNA helicase DDX1 | 4.12 | 2.11 | 2.01 | 0.00489 | 0.236 | 0.179 | TRUE | FALSE | FALSE |
| **DDX17** | RNA helicase | 5.4 | 2.4 | 3 | 0.000641 | 0.197 | 0.0411 | TRUE | FALSE | TRUE |
| **DDX31** | DEAD-box helicase 31 | 4.21 | 1.78 | 2.43 | 0.0161 | 0.373 | 0.21 | TRUE | FALSE | FALSE |
| **DDX39B** | Spliceosome RNA helicase DDX39B | 0.178 | -0.933 | 1.11 | 0.924 | 0.689 | 0.604 | FALSE | FALSE | FALSE |
| **DDX3X** | RNA helicase | 4.71 | 1.18 | 3.54 | 0.0117 | 0.599 | 0.0866 | TRUE | FALSE | FALSE |
| **DEFB10** | Beta-defensin 10 | 0.743 | -0.828 | 1.57 | 0.51 | 0.522 | 0.203 | FALSE | FALSE | FALSE |
| **DEFB12** | Beta-defensin 12 | -2.08 | -1.46 | -0.621 | 0.0983 | 0.335 | 0.68 | FALSE | FALSE | FALSE |
| **DEFB2** | Beta-defensin 2 | 0.0499 | -1.01 | 1.06 | 0.972 | 0.553 | 0.497 | FALSE | FALSE | FALSE |

| | | | | | | | | | | |
|---|---|---|---|---|---|---|---|---|---|---|
| **DEK** | DEK protein | -1.98 | -0.113 | -1.87 | 0.147 | 0.955 | 0.222 | FALSE | FALSE | FALSE |
| **DGKA** | Diacylglycerol kinase alpha | -0.0221 | 2.35 | -2.37 | 0.991 | 0.253 | 0.177 | FALSE | FALSE | FALSE |
| **DHX15** | RNA helicase | 1.61 | 0.947 | 0.663 | 0.281 | 0.599 | 0.714 | FALSE | FALSE | FALSE |
| **DHX30** | RNA helicase | -0.736 | -0.217 | -0.519 | 0.552 | 0.901 | 0.72 | FALSE | FALSE | FALSE |
| **DHX36** | ATP-dependent DNA/RNA helicase DHX36 | 2.17 | 2.92 | -0.746 | 0.439 | 0.365 | 0.841 | FALSE | FALSE | FALSE |
| **DHX57** | DExH-box helicase 57 | -1.36 | -1.61 | 0.247 | 0.249 | 0.268 | 0.865 | FALSE | FALSE | FALSE |
| **DHX9** | ATP-dependent RNA helicase A | 1.33 | 0.608 | 0.721 | 0.428 | 0.781 | 0.722 | FALSE | FALSE | FALSE |
| **DIAPH1** | Uncharacterized protein | -0.459 | -1.47 | 1.01 | 0.774 | 0.393 | 0.552 | FALSE | FALSE | FALSE |
| **DIP2B** | Disco interacting B | -0.35 | -0.475 | 0.125 | 0.834 | 0.804 | 0.942 | FALSE | FALSE | FALSE |
| **DIS3L2** | DIS3-like exonuclease 2 | -2.63 | -1.26 | -1.36 | 0.0419 | 0.393 | 0.329 | TRUE | FALSE | FALSE |
| **DKC1** | DKC1 protein | -0.988 | -1.23 | 0.247 | 0.345 | 0.316 | 0.853 | FALSE | FALSE | FALSE |
| **DLG1** | Discs large MAGUK scaffold protein 1 | 0.594 | 1.71 | -1.12 | 0.751 | 0.393 | 0.571 | FALSE | FALSE | FALSE |
| **DNAH1** | Dynein axonemal heavy chain 1 | 1.16 | 1.68 | -0.517 | 0.3 | 0.253 | 0.696 | FALSE | FALSE | FALSE |
| **DNAH5** | Dynein axonemal heavy chain 5 | -1.69 | -1.94 | 0.247 | 0.172 | 0.236 | 0.873 | FALSE | FALSE | FALSE |
| **DNAJA1** | DnaJ homolog subfamily A member 1 | -0.234 | -0.205 | -0.0288 | 0.886 | 0.918 | 0.985 | FALSE | FALSE | FALSE |
| **DNAJA2** | DnaJ homolog subfamily A member 2 | 2.49 | 1.02 | 1.47 | 0.0315 | 0.438 | 0.245 | TRUE | FALSE | FALSE |

| | | | | | | | | | | |
|---|---|---|---|---|---|---|---|---|---|---|
| **DNAJB1** | DnaJ homolog subfamily B member 1 | -0.82 | 1.06 | -1.88 | 0.63 | 0.583 | 0.277 | FALSE | FALSE | FALSE |
| **DNAJC9** | DnaJ heat shock protein family (Hsp40) member C9 | -1.95 | -1.48 | -0.467 | 0.0925 | 0.292 | 0.744 | FALSE | FALSE | FALSE |
| **DNM2** | Dynamin GTPase | 1.25 | -0.987 | 2.23 | 0.274 | 0.446 | 0.0809 | FALSE | FALSE | FALSE |
| **DNPEP** | Aspartyl aminopeptidase | 0.158 | 1.94 | -1.79 | 0.905 | 0.236 | 0.197 | FALSE | FALSE | FALSE |
| **DOCK1** | Dedicator of cytokinesis 1 | -1.67 | -0.721 | -0.951 | 0.324 | 0.734 | 0.62 | FALSE | FALSE | FALSE |
| **DOCK2** | Dedicator of cytokinesis 2 | 0.698 | 0.919 | -0.221 | 0.591 | 0.521 | 0.88 | FALSE | FALSE | FALSE |
| **DOCK7** | Dedicator of cytokinesis 7 | -1.23 | -1.48 | 0.247 | 0.261 | 0.271 | 0.857 | FALSE | FALSE | FALSE |
| **DOCK9** | Dedicator of cytokinesis 9 | -0.25 | 0.201 | -0.451 | 0.892 | 0.933 | 0.824 | FALSE | FALSE | FALSE |
| **DPP3** | Dipeptidyl peptidase 3 | 1.25 | 0.661 | 0.585 | 0.402 | 0.72 | 0.744 | FALSE | FALSE | FALSE |
| **DPP4** | Dipeptidyl peptidase 4 | -0.482 | -4.32 | 3.84 | 0.809 | 0.0947 | 0.0629 | FALSE | FALSE | FALSE |
| **DPYSL2** | Dihydropyrimidinase-related protein 2 | 0.541 | 0.855 | -0.314 | 0.613 | 0.465 | 0.815 | FALSE | FALSE | FALSE |
| **DPYSL3** | DPYSL3 protein | 0.394 | 0.243 | 0.152 | 0.75 | 0.881 | 0.915 | FALSE | FALSE | FALSE |
| **DRG1** | Developmentally-regulated GTP-binding protein 1 | -0.663 | 0.556 | -1.22 | 0.572 | 0.69 | 0.318 | FALSE | FALSE | FALSE |
| **DSG2** | Desmoglein 2 | -1.15 | -1.39 | 0.247 | 0.3 | 0.289 | 0.857 | FALSE | FALSE | FALSE |
| **DSP** | Desmoplakin | 6.76 | 3.71 | 3.05 | 0.012 | 0.261 | 0.282 | TRUE | FALSE | FALSE |
| **DSTN** | Destrin | -1.21 | -0.773 | -0.44 | 0.249 | 0.538 | 0.73 | FALSE | FALSE | FALSE |

| | | | | | | | | | | |
|---|---|---|---|---|---|---|---|---|---|---|
| **DYNC1H1** | Dynein cytoplasmic 1 heavy chain 1 | 6.47 | 0.398 | 6.07 | 9.68E-05 | 0.788 | 0.000811 | TRUE | FALSE | TRUE |
| **DYNC1I2** | Cytoplasmic dynein 1 intermediate chain 2 | 0.759 | 1.05 | -0.294 | 0.527 | 0.426 | 0.846 | FALSE | FALSE | FALSE |
| **DYNC1LI1** | Dynein light intermediate chain | 1.02 | 1.28 | -0.265 | 0.348 | 0.316 | 0.847 | FALSE | FALSE | FALSE |
| **DYNC1LI2** | Dynein light intermediate chain | -0.224 | 1.38 | -1.61 | 0.865 | 0.285 | 0.183 | FALSE | FALSE | FALSE |
| **DYNLL1** | Dynein light chain 1 cytoplasmic | 0.404 | -0.145 | 0.549 | 0.731 | 0.933 | 0.668 | FALSE | FALSE | FALSE |
| **DYNLRB1** | Dynein light chain roadblock-type 1 | -0.415 | 0.263 | -0.678 | 0.707 | 0.858 | 0.563 | FALSE | FALSE | FALSE |
| **DYNLT1** | Dynein light chain Tctex-type 1 | -1.28 | -1.53 | 0.247 | 0.247 | 0.265 | 0.857 | FALSE | FALSE | FALSE |
| **E1BAY6** | Uncharacterized protein | -1.25 | 0.456 | -1.71 | 0.391 | 0.816 | 0.277 | FALSE | FALSE | FALSE |
| **ECM1** | Extracellular matrix protein 1 | -0.864 | -0.165 | -0.699 | 0.444 | 0.926 | 0.583 | FALSE | FALSE | FALSE |
| **ECM2** | Extracellular matrix protein 2 | -0.531 | 0.161 | -0.692 | 0.726 | 0.94 | 0.677 | FALSE | FALSE | FALSE |
| **EEA1** | Early endosome antigen 1 | 4.59 | 2.6 | 1.99 | 0.00498 | 0.224 | 0.229 | TRUE | FALSE | FALSE |
| **EEF1A1** | Elongation factor 1-alpha 1 | 3.3 | 0.516 | 2.78 | 0.00724 | 0.723 | 0.037 | TRUE | FALSE | TRUE |
| **EEF1B** | Elongation factor 1-beta | 0.981 | 0.341 | 0.64 | 0.413 | 0.838 | 0.647 | FALSE | FALSE | FALSE |
| **EEF1D** | Elongation factor 1-delta | 1.97 | -0.302 | 2.28 | 0.175 | 0.883 | 0.166 | FALSE | FALSE | FALSE |

| | | | | | | | | | | |
|---|---|---|---|---|---|---|---|---|---|---|
| **EEF1E1** | Eukaryotic translation elongation factor 1 epsilon 1 | 1.35 | 0.942 | 0.407 | 0.388 | 0.621 | 0.841 | FALSE | FALSE | FALSE |
| **EEF1G** | Elongation factor 1-gamma | 1.47 | -0.785 | 2.26 | 0.393 | 0.709 | 0.229 | FALSE | FALSE | FALSE |
| **EEF2** | Elongation factor 2 | 1.72 | -0.3 | 2.02 | 0.18 | 0.867 | 0.162 | FALSE | FALSE | FALSE |
| **EFEMP1** | EGF containing fibulin extracellular matrix protein 1 | -0.899 | -0.397 | -0.502 | 0.609 | 0.865 | 0.816 | FALSE | FALSE | FALSE |
| **EFHD2** | EF-hand domain-containing protein D2 | -0.13 | 4.35 | -4.48 | 0.9 | 0.00335 | 0.00124 | FALSE | TRUE | TRUE |
| **EFNA1** | Ephrin-A1 | -0.648 | -0.53 | -0.118 | 0.565 | 0.69 | 0.924 | FALSE | FALSE | FALSE |
| **EFR3A** | EFR3 homolog A | -1.39 | -0.52 | -0.875 | 0.283 | 0.754 | 0.549 | FALSE | FALSE | FALSE |
| **EFTUD2** | 116 kDa U5 small nuclear ribonucleoprotein component | 0.628 | 0.697 | -0.0692 | 0.692 | 0.707 | 0.975 | FALSE | FALSE | FALSE |
| **EHD1** | EH domain-containing protein 1 | 1.09 | -0.878 | 1.97 | 0.341 | 0.501 | 0.116 | FALSE | FALSE | FALSE |
| **EHD4** | EH domain containing 4 | -0.293 | -0.539 | 0.246 | 0.817 | 0.69 | 0.857 | FALSE | FALSE | FALSE |
| **EIF2A** | Eukaryotic translation initiation factor 2A | -0.511 | -0.757 | 0.247 | 0.608 | 0.484 | 0.841 | FALSE | FALSE | FALSE |
| **EIF2AK2** | Eukaryotic translation initiation factor 2-alpha kinase 2 | -0.808 | -0.255 | -0.553 | 0.501 | 0.876 | 0.692 | FALSE | FALSE | FALSE |

| | | | | | | | | | | |
|---|---|---|---|---|---|---|---|---|---|---|
| **EIF2B3** | Translation initiation factor eIF-2B subunit gamma | -0.783 | 0.696 | -1.48 | 0.597 | 0.689 | 0.327 | FALSE | FALSE | FALSE |
| **EIF2B4** | Translation initiation factor eIF-2B subunit delta | 1.67 | 1.07 | 0.596 | 0.0837 | 0.349 | 0.591 | FALSE | FALSE | FALSE |
| **EIF2S1** | Eukaryotic translation initiation factor 2 subunit 1 | 1.61 | 0.841 | 0.767 | 0.348 | 0.688 | 0.704 | FALSE | FALSE | FALSE |
| **EIF2S2** | Eukaryotic translation initiation factor 2 subunit 2 | -0.0718 | 0.691 | -0.763 | 0.955 | 0.644 | 0.575 | FALSE | FALSE | FALSE |
| **EIF2S3** | Eukaryotic translation initiation factor 2 subunit 3 | 4.77 | 1.96 | 2.81 | 0.00278 | 0.265 | 0.0797 | TRUE | FALSE | FALSE |
| **EIF3A** | Eukaryotic translation initiation factor 3 subunit A | -1.46 | -0.136 | -1.32 | 0.221 | 0.939 | 0.303 | FALSE | FALSE | FALSE |
| **EIF3B** | Eukaryotic translation initiation factor 3 subunit B | -0.638 | 0.223 | -0.861 | 0.55 | 0.876 | 0.433 | FALSE | FALSE | FALSE |
| **EIF3C** | Eukaryotic translation initiation factor 3 subunit C | -0.26 | 0.661 | -0.921 | 0.84 | 0.628 | 0.434 | FALSE | FALSE | FALSE |

| | | | | | | | | | | |
|---|---|---|---|---|---|---|---|---|---|---|
| **EIF3D** | Eukaryotic translation initiation factor 3 subunit D | -0.246 | 1.22 | -1.46 | 0.871 | 0.393 | 0.277 | FALSE | FALSE | FALSE |
| **EIF3E** | Eukaryotic translation initiation factor 3 subunit E | 1.07 | 0.562 | 0.504 | 0.473 | 0.763 | 0.786 | FALSE | FALSE | FALSE |
| **EIF3F** | Eukaryotic translation initiation factor 3 subunit F | -0.439 | -0.13 | -0.309 | 0.658 | 0.931 | 0.796 | FALSE | FALSE | FALSE |
| **EIF3G** | Eukaryotic translation initiation factor 3 subunit G | -0.269 | 1.87 | -2.14 | 0.832 | 0.224 | 0.0808 | FALSE | FALSE | FALSE |
| **EIF3H** | Eukaryotic translation initiation factor 3 subunit H | -0.223 | 0.487 | -0.71 | 0.849 | 0.69 | 0.516 | FALSE | FALSE | FALSE |
| **EIF3I** | Eukaryotic translation initiation factor 3 subunit I | -0.803 | 0.12 | -0.922 | 0.551 | 0.952 | 0.522 | FALSE | FALSE | FALSE |
| **EIF3L** | Eukaryotic translation initiation factor 3 subunit L | -0.734 | 0.039 | -0.773 | 0.5 | 0.978 | 0.511 | FALSE | FALSE | FALSE |
| **EIF4A1** | Eukaryotic initiation factor 4A-I | -0.0498 | -1.52 | 1.47 | 0.97 | 0.321 | 0.282 | FALSE | FALSE | FALSE |
| **EIF4A2** | RNA helicase | 0.158 | 0.581 | -0.422 | 0.888 | 0.599 | 0.697 | FALSE | FALSE | FALSE |

| | | | | | | | | | | |
|---|---|---|---|---|---|---|---|---|---|---|
| **EIF4A3** | Eukaryotic initiation factor 4A-III | 0.158 | 2.32 | -2.16 | 0.915 | 0.236 | 0.193 | FALSE | FALSE | FALSE |
| **EIF4E** | Eukaryotic translation initiation factor 4E | -0.943 | -0.46 | -0.484 | 0.439 | 0.767 | 0.744 | FALSE | FALSE | FALSE |
| **EIF4G1** | Eukaryotic translation initiation factor 4 gamma 1 | 3.22 | 3.01 | 0.203 | 0.0878 | 0.236 | 0.926 | FALSE | FALSE | FALSE |
| **EIF5A** | Eukaryotic translation initiation factor 5A-1 | 1.25 | 0.00587 | 1.24 | 0.314 | 0.996 | 0.345 | FALSE | FALSE | FALSE |
| **ELANE** | ELA2 protein | -4.57 | -1.61 | -2.96 | 0.00839 | 0.397 | 0.114 | TRUE | FALSE | FALSE |
| **ELAVL1** | ELAV-like protein | 1.62 | 1.57 | 0.046 | 0.174 | 0.276 | 0.977 | FALSE | FALSE | FALSE |
| **ELMO1** | Engulfment and cell motility 1 | -2.01 | -0.219 | -1.79 | 0.219 | 0.933 | 0.31 | FALSE | FALSE | FALSE |
| **ELOC** | Elongin-C | -0.346 | 0.628 | -0.974 | 0.765 | 0.642 | 0.407 | FALSE | FALSE | FALSE |
| **ELP1** | Elongator complex protein 1 | 1.3 | 2.63 | -1.33 | 0.51 | 0.268 | 0.545 | FALSE | FALSE | FALSE |
| **ELP3** | Elongator complex protein 3 | -0.516 | 0.602 | -1.12 | 0.654 | 0.648 | 0.332 | FALSE | FALSE | FALSE |
| **EMB** | Embigin | 0.942 | 0.0655 | 0.877 | 0.623 | 0.978 | 0.68 | FALSE | FALSE | FALSE |
| **EMILIN2** | Elastin microfibril interfacer 2 | -0.228 | 0.334 | -0.563 | 0.852 | 0.809 | 0.642 | FALSE | FALSE | FALSE |
| **EML2** | EMAP like 2 | -0.723 | -0.807 | 0.0841 | 0.662 | 0.672 | 0.969 | FALSE | FALSE | FALSE |
| **EML3** | EMAP like 3 | -0.699 | -0.946 | 0.247 | 0.474 | 0.393 | 0.846 | FALSE | FALSE | FALSE |
| **EML4** | EMAP like 4 | 2.87 | 1.53 | 1.33 | 0.082 | 0.418 | 0.467 | FALSE | FALSE | FALSE |
| **ENO1** | Alpha-enolase | -1.45 | -1.7 | 0.251 | 0.254 | 0.272 | 0.874 | FALSE | FALSE | FALSE |

| | | | | | | | | | | |
|---|---|---|---|---|---|---|---|---|---|---|
| **ENPP1** | Ectonucleotide pyrophosphatase/phosphodiesterase 1 | 1.65 | 0.283 | 1.36 | 0.117 | 0.852 | 0.239 | FALSE | FALSE | FALSE |
| **ENPP3** | Ectonucleotide pyrophosphatase/phosphodiesterase family member 3 | 6.26 | 0.414 | 5.84 | 0.00374 | 0.876 | 0.0139 | TRUE | FALSE | TRUE |
| **ENTPD2** | Ectonucleoside triphosphate diphosphohydrolase 2 | 2.41 | 0.243 | 2.16 | 0.037 | 0.881 | 0.0919 | TRUE | FALSE | FALSE |
| **EPB41** | Protein 4.1 | -0.00114 | 0.771 | -0.772 | 0.999 | 0.67 | 0.654 | FALSE | FALSE | FALSE |
| **EPB41L3** | Erythrocyte membrane protein band 4.1 like 3 | -0.569 | -0.402 | -0.167 | 0.578 | 0.749 | 0.889 | FALSE | FALSE | FALSE |
| **EPCAM** | Epithelial cell adhesion molecule | 2.25 | 1.14 | 1.11 | 0.0517 | 0.393 | 0.377 | FALSE | FALSE | FALSE |
| **EPHX1** | Epoxide hydrolase | -0.865 | -1.11 | 0.247 | 0.405 | 0.36 | 0.853 | FALSE | FALSE | FALSE |
| **EPHX2** | Epoxide hydrolase 2 | 0.345 | 0.997 | -0.651 | 0.881 | 0.66 | 0.78 | FALSE | FALSE | FALSE |
| **EPM2AIP1** | EPM2A interacting protein 1 | -0.208 | 1.52 | -1.73 | 0.892 | 0.316 | 0.222 | FALSE | FALSE | FALSE |
| **EPN1** | Epsin 1 | 0.158 | 1.28 | -1.12 | 0.895 | 0.316 | 0.327 | FALSE | FALSE | FALSE |
| **EPPK1** | Epiplakin 1 | -0.33 | 0.856 | -1.19 | 0.755 | 0.452 | 0.277 | FALSE | FALSE | FALSE |
| **EPRS1** | Glutamyl-prolyl-tRNA synthetase 1 | 5.45 | 0.761 | 4.69 | 0.015 | 0.797 | 0.0659 | TRUE | FALSE | FALSE |
| **EPS8** | Epidermal growth factor receptor pathway substrate 8 | 4.25 | 0.326 | 3.93 | 0.0323 | 0.915 | 0.0808 | TRUE | FALSE | FALSE |
| **EPS8L1** | EPS8 like 1 | 2.9 | -1.36 | 4.26 | 0.0812 | 0.484 | 0.0299 | FALSE | FALSE | TRUE |

| | | | | | | | | | | |
|---|---|---|---|---|---|---|---|---|---|---|
| **EPS8L2** | EPS8 like 2 | 0.348 | -2.09 | 2.44 | 0.873 | 0.323 | 0.216 | FALSE | FALSE | FALSE |
| **ERGIC1** | Endoplasmic reticulum-Golgi intermediate compartment protein | 2.89 | 1.29 | 1.6 | 0.0376 | 0.416 | 0.287 | TRUE | FALSE | FALSE |
| **ERH** | Enhancer of rudimentary homolog | -0.653 | -0.0834 | -0.569 | 0.562 | 0.958 | 0.654 | FALSE | FALSE | FALSE |
| **ERLIN1** | ER lipid raft associated 1 | 2.17 | 2.23 | -0.0565 | 0.114 | 0.236 | 0.975 | FALSE | FALSE | FALSE |
| **ERP29** | Endoplasmic reticulum resident protein 29 | -1.15 | -1.39 | 0.247 | 0.284 | 0.279 | 0.856 | FALSE | FALSE | FALSE |
| **ERP44** | Endoplasmic reticulum resident protein 44 | 3.22 | 1.31 | 1.91 | 0.0306 | 0.439 | 0.239 | TRUE | FALSE | FALSE |
| **ESD** | S-formylglutathione hydrolase | -0.0228 | -0.834 | 0.811 | 0.99 | 0.633 | 0.62 | FALSE | FALSE | FALSE |
| **ESYT1** | Extended synaptotagmin 1 | 0.736 | 1.77 | -1.03 | 0.557 | 0.256 | 0.423 | FALSE | FALSE | FALSE |
| **EVPL** | Envoplakin | -0.444 | 0.436 | -0.879 | 0.679 | 0.733 | 0.416 | FALSE | FALSE | FALSE |
| **EXOC4** | Exocyst complex component Sec8 | -0.86 | 1.12 | -1.98 | 0.613 | 0.565 | 0.259 | FALSE | FALSE | FALSE |
| **EXOC8** | Exocyst complex component 8 | -0.524 | -0.506 | -0.018 | 0.667 | 0.722 | 0.988 | FALSE | FALSE | FALSE |
| **EZR** | Ezrin | 1.39 | -1.02 | 2.41 | 0.243 | 0.447 | 0.0739 | FALSE | FALSE | FALSE |
| **F11R** | Junctional adhesion molecule A | -1.8 | -1.62 | -0.181 | 0.0979 | 0.256 | 0.894 | FALSE | FALSE | FALSE |
| **F13A1** | Coagulation factor XIII A chain | -0.018 | -0.221 | 0.203 | 0.992 | 0.933 | 0.919 | FALSE | FALSE | FALSE |

| | | | | | | | | | | |
|---|---|---|---|---|---|---|---|---|---|---|
| **F1MZ96** | Uncharacterized protein | -0.96 | 0.361 | -1.32 | 0.383 | 0.804 | 0.268 | FALSE | FALSE | FALSE |
| **F1N160** | Uncharacterized protein | -1.21 | 0.116 | -1.32 | 0.381 | 0.955 | 0.363 | FALSE | FALSE | FALSE |
| **F1N4S4** | MHC_I-like_Ag-recog domain-containing protein | 0.158 | 0.927 | -0.769 | 0.892 | 0.413 | 0.474 | FALSE | FALSE | FALSE |
| **FABP5** | Fatty acid-binding protein 5 | 1.36 | -0.802 | 2.17 | 0.375 | 0.665 | 0.197 | FALSE | FALSE | FALSE |
| **FAM120A** | Constitutive coactivator of PPAR-gamma-like protein 1 | 2.5 | 2.95 | -0.454 | 0.104 | 0.194 | 0.824 | FALSE | FALSE | FALSE |
| **FAM234A** | Protein FAM234A | -0.496 | 0.719 | -1.21 | 0.697 | 0.629 | 0.347 | FALSE | FALSE | FALSE |
| **FAM3C** | Protein FAM3C | -0.114 | 1.21 | -1.33 | 0.927 | 0.4 | 0.329 | FALSE | FALSE | FALSE |
| **FAM3D** | FAM3 metabolism regulating signaling molecule D | -1.54 | -1.74 | 0.199 | 0.194 | 0.256 | 0.892 | FALSE | FALSE | FALSE |
| **FAM98B** | Family with sequence similarity 98 member B | 0.349 | 1.15 | -0.797 | 0.802 | 0.423 | 0.575 | FALSE | FALSE | FALSE |
| **FARP1** | FERM ARH/RhoGEF and pleckstrin domain protein 1 | -1.14 | -1.01 | -0.134 | 0.244 | 0.371 | 0.909 | FALSE | FALSE | FALSE |
| **FARSA** | Phenylalanine--tRNA ligase alpha subunit | 0.333 | 1.83 | -1.49 | 0.76 | 0.214 | 0.194 | FALSE | FALSE | FALSE |
| **FARSB** | Phenylalanine--tRNA ligase beta subunit | -1.96 | -1.42 | -0.541 | 0.0888 | 0.316 | 0.701 | FALSE | FALSE | FALSE |
| **FASN** | Fatty acid synthase | 2.15 | -1.36 | 3.52 | 0.238 | 0.519 | 0.0854 | FALSE | FALSE | FALSE |

| | | | | | | | | | | |
|---|---|---|---|---|---|---|---|---|---|---|
| **FAT2** | FAT atypical cadherin 2 | 0.158 | 1.71 | -1.55 | 0.915 | 0.332 | 0.331 | FALSE | FALSE | FALSE |
| **FAU** | 40S ribosomal protein S30 | 5.93 | 2.91 | 3.02 | 0.000892 | 0.186 | 0.0752 | TRUE | FALSE | FALSE |
| **FBXO6** | F-box only protein 6 | -1.92 | -2.16 | 0.247 | 0.152 | 0.236 | 0.879 | FALSE | FALSE | FALSE |
| **FCER1G** | High affinity immunoglobulin epsilon receptor subunit gamma | -1.33 | 0.169 | -1.5 | 0.447 | 0.952 | 0.423 | FALSE | FALSE | FALSE |
| **FCGBP** | Uncharacterized protein | 5.26 | -1.67 | 6.93 | 0.00736 | 0.431 | 0.00394 | TRUE | FALSE | TRUE |
| **FCN2** | Ficolin-2 | -2.74 | -0.643 | -2.1 | 0.0856 | 0.756 | 0.236 | FALSE | FALSE | FALSE |
| **FCSK** | FUK protein | -0.658 | 0.805 | -1.46 | 0.537 | 0.502 | 0.202 | FALSE | FALSE | FALSE |
| **FERMT3** | Fermitin family homolog 3 | -2.6 | -2.3 | -0.295 | 0.194 | 0.333 | 0.901 | FALSE | FALSE | FALSE |
| **FGG** | FGG protein | -0.687 | -0.754 | 0.0672 | 0.615 | 0.639 | 0.972 | FALSE | FALSE | FALSE |
| **FH** | Fumarate hydratase mitochondrial | 0.158 | 2.15 | -1.99 | 0.907 | 0.236 | 0.185 | FALSE | FALSE | FALSE |
| **FHL2** | Four and a half LIM domains protein 2 | -1.4 | -0.623 | -0.774 | 0.36 | 0.744 | 0.658 | FALSE | FALSE | FALSE |
| **FKBP15** | Peptidylprolyl isomerase | -0.37 | -0.448 | 0.0782 | 0.823 | 0.822 | 0.969 | FALSE | FALSE | FALSE |
| **FKBP4** | Peptidyl-prolyl cis-trans isomerase FKBP4 | -0.271 | -0.235 | -0.0355 | 0.869 | 0.903 | 0.985 | FALSE | FALSE | FALSE |
| **FLII** | FLII actin remodeling protein | -0.329 | 0.244 | -0.573 | 0.75 | 0.858 | 0.601 | FALSE | FALSE | FALSE |
| **FLNA** | Filamin A | -0.0963 | -0.399 | 0.303 | 0.934 | 0.796 | 0.841 | FALSE | FALSE | FALSE |
| **FLNB** | Filamin B | 5.6 | 1.28 | 4.32 | 0.0663 | 0.745 | 0.211 | FALSE | FALSE | FALSE |
| **FLOT1** | Flotillin-1 | -0.22 | 0.756 | -0.975 | 0.862 | 0.532 | 0.377 | FALSE | FALSE | FALSE |

| | | | | | | | | | | |
|---|---|---|---|---|---|---|---|---|---|---|
| **FLVCR2** | Feline leukemia virus subgroup C cellular receptor family member 2 | 0.14 | -1.66 | 1.8 | 0.928 | 0.363 | 0.29 | FALSE | FALSE | FALSE |
| **FMNL1** | Formin like 1 | 0.275 | -0.745 | 1.02 | 0.842 | 0.605 | 0.421 | FALSE | FALSE | FALSE |
| **FMR1** | Synaptic functional regulator FMR1 | 3.37 | 2.26 | 1.12 | 0.0278 | 0.256 | 0.511 | TRUE | FALSE | FALSE |
| **FN1** | Fibronectin | 2.44 | 0.0741 | 2.37 | 0.166 | 0.975 | 0.227 | FALSE | FALSE | FALSE |
| **FOLR1** | Folate receptor alpha | 0.217 | -1.64 | 1.85 | 0.917 | 0.512 | 0.416 | FALSE | FALSE | FALSE |
| **FOLR1.1** | Folate receptor alpha | 0.32 | 0.35 | -0.0299 | 0.865 | 0.87 | 0.986 | FALSE | FALSE | FALSE |
| **FRK** | Tyrosine-protein kinase | -0.832 | -1.91 | 1.07 | 0.676 | 0.379 | 0.619 | FALSE | FALSE | FALSE |
| **FRYL** | FRY like transcription coactivator | -1.79 | 0.185 | -1.97 | 0.285 | 0.941 | 0.277 | FALSE | FALSE | FALSE |
| **FSCN1** | Fascin | -0.77 | -1.85 | 1.08 | 0.636 | 0.316 | 0.526 | FALSE | FALSE | FALSE |
| **FTH1** | Ferritin heavy chain | -0.0391 | 0.162 | -0.201 | 0.97 | 0.919 | 0.876 | FALSE | FALSE | FALSE |
| **FTL** | Ferritin light chain | -1.09 | 0.101 | -1.19 | 0.567 | 0.967 | 0.562 | FALSE | FALSE | FALSE |
| **FUCA1** | Tissue alpha-L-fucosidase | -3.99 | -3.48 | -0.507 | 0.0137 | 0.141 | 0.807 | TRUE | FALSE | FALSE |
| **FUCA2** | Alpha-L-fucosidase | -0.432 | -1.64 | 1.21 | 0.74 | 0.271 | 0.355 | FALSE | FALSE | FALSE |
| **FUS** | RNA-binding protein FUS | -0.247 | 0.721 | -0.968 | 0.84 | 0.565 | 0.386 | FALSE | FALSE | FALSE |
| **FXR1** | RNA-binding protein FXR1 | 0.514 | 0.00812 | 0.506 | 0.612 | 0.995 | 0.654 | FALSE | FALSE | FALSE |
| **G3BP1** | Ras GTPase-activating protein-binding protein 1 | -0.871 | -0.287 | -0.584 | 0.503 | 0.871 | 0.703 | FALSE | FALSE | FALSE |

| | | | | | | | | | | |
|---|---|---|---|---|---|---|---|---|---|---|
| **G3MXB5** | Uncharacterized protein | 0.885 | -1.56 | 2.45 | 0.63 | 0.43 | 0.207 | FALSE | FALSE | FALSE |
| **G3N0V0** | Uncharacterized protein | -2.53 | 0.137 | -2.67 | 0.0801 | 0.952 | 0.101 | FALSE | FALSE | FALSE |
| **G3N2D7** | Ig-like domain-containing protein | -1.41 | 0.201 | -1.61 | 0.447 | 0.943 | 0.416 | FALSE | FALSE | FALSE |
| **G5E5A7** | Uncharacterized protein | 3.94 | 0.951 | 2.99 | 0.00839 | 0.585 | 0.0693 | TRUE | FALSE | FALSE |
| **G5E5T5** | Uncharacterized protein | -1.29 | 0.0741 | -1.37 | 0.322 | 0.967 | 0.327 | FALSE | FALSE | FALSE |
| **G5E5V1** | Ig-like domain-containing protein | -1.31 | 0.31 | -1.62 | 0.299 | 0.86 | 0.235 | FALSE | FALSE | FALSE |
| **G6PD** | Glucose-6-phosphate 1-dehydrogenase | -0.432 | -1.64 | 1.21 | 0.785 | 0.342 | 0.445 | FALSE | FALSE | FALSE |
| **GAA** | Lysosomal alpha-glucosidase | 0.158 | 1.6 | -1.44 | 0.905 | 0.289 | 0.288 | FALSE | FALSE | FALSE |
| **GAK** | Cyclin G associated kinase | -0.569 | 0.59 | -1.16 | 0.689 | 0.728 | 0.423 | FALSE | FALSE | FALSE |
| **GALNT3** | Polypeptide N-acetylgalactosaminyltransferase | -2.11 | 0.0476 | -2.16 | 0.229 | 0.985 | 0.254 | FALSE | FALSE | FALSE |
| **GALNT6** | Polypeptide N-acetylgalactosaminyltransferase 6 | -0.37 | 0.174 | -0.543 | 0.755 | 0.918 | 0.677 | FALSE | FALSE | FALSE |
| **GALNT7** | Polypeptide N-acetylgalactosaminyltransferase | -2.72 | -1.46 | -1.26 | 0.151 | 0.512 | 0.559 | FALSE | FALSE | FALSE |
| **GANAB** | Glucosidase II alpha subunit | -2.14 | -0.949 | -1.2 | 0.127 | 0.578 | 0.431 | FALSE | FALSE | FALSE |

| | | | | | | | | | | |
|---|---|---|---|---|---|---|---|---|---|---|
| **GAPDH** | Glyceraldehyde-3-phosphate dehydrogenase | -1.02 | -1.24 | 0.222 | 0.369 | 0.342 | 0.873 | FALSE | FALSE | FALSE |
| **GARS1** | Glycine--tRNA ligase | -0.432 | -1.44 | 1 | 0.797 | 0.409 | 0.561 | FALSE | FALSE | FALSE |
| **GART** | Trifunctional purine biosynthetic protein adenosine-3 | -1.29 | -0.685 | -0.601 | 0.373 | 0.69 | 0.726 | FALSE | FALSE | FALSE |
| **GAS6** | Growth arrest specific 6 | -2.38 | -0.81 | -1.57 | 0.324 | 0.8 | 0.56 | FALSE | FALSE | FALSE |
| **GBE1** | 1'4-alpha-glucan branching enzyme | 0.488 | 0.522 | -0.0337 | 0.739 | 0.765 | 0.985 | FALSE | FALSE | FALSE |
| **GCA** | Grancalcin | -1.01 | 0.437 | -1.45 | 0.362 | 0.754 | 0.229 | FALSE | FALSE | FALSE |
| **GCLC** | Glutamate--cysteine ligase | 0.612 | -0.961 | 1.57 | 0.666 | 0.538 | 0.277 | FALSE | FALSE | FALSE |
| **GCN1** | GCN1 activator of EIF2AK4 | -0.217 | -0.0178 | -0.199 | 0.892 | 0.993 | 0.901 | FALSE | FALSE | FALSE |
| **GCNT2** | Glucosaminyl (N-acetyl) transferase 2 (I blood group) | -0.666 | -1.88 | 1.21 | 0.601 | 0.236 | 0.345 | FALSE | FALSE | FALSE |
| **GCNT3** | Beta-1'3-galactosyl-O-glycosyl-glycoprotein beta-1 | -0.0224 | 1.22 | -1.24 | 0.987 | 0.373 | 0.331 | FALSE | FALSE | FALSE |
| **GDA** | Guanine deaminase | -1.17 | -1.3 | 0.139 | 0.377 | 0.388 | 0.926 | FALSE | FALSE | FALSE |
| **GDI1** | Rab GDP dissociation inhibitor alpha | -0.388 | -0.301 | -0.0876 | 0.765 | 0.862 | 0.957 | FALSE | FALSE | FALSE |
| **GDI2** | Rab GDP dissociation inhibitor | 1.69 | -0.924 | 2.62 | 0.3 | 0.643 | 0.146 | FALSE | FALSE | FALSE |

| | | | | | | | | | | |
|---|---|---|---|---|---|---|---|---|---|---|
| **GFPT1** | Glutamine--fructose-6-phosphate transaminase (isomerizing) | 1.19 | 0.341 | 0.851 | 0.649 | 0.931 | 0.784 | FALSE | FALSE | FALSE |
| **GGT1** | Glutathione hydrolase | 2.6 | 1.52 | 1.08 | 0.0419 | 0.326 | 0.431 | TRUE | FALSE | FALSE |
| **GIMAP4** | GTPase IMAP family member 4 | 0.158 | 1.69 | -1.53 | 0.927 | 0.4 | 0.417 | FALSE | FALSE | FALSE |
| **GIMAP7** | GTPase IMAP family member 7 | 0.158 | -2.99 | 3.14 | 0.922 | 0.201 | 0.0866 | FALSE | FALSE | FALSE |
| **GIPC2** | PDZ domain-containing protein GIPC2 | 0.055 | -0.902 | 0.957 | 0.97 | 0.599 | 0.549 | FALSE | FALSE | FALSE |
| **GIT1** | GIT ArfGAP 1 | -0.467 | -0.00537 | -0.462 | 0.695 | 0.996 | 0.738 | FALSE | FALSE | FALSE |
| **GLG1** | Golgi apparatus protein 1 | 0.804 | 1.01 | -0.204 | 0.573 | 0.532 | 0.901 | FALSE | FALSE | FALSE |
| **GLIPR2** | GLI pathogenesis-related 2 | 0.418 | -1.1 | 1.52 | 0.811 | 0.558 | 0.363 | FALSE | FALSE | FALSE |
| **GLOD4** | GLOD4 protein | -1.07 | -0.875 | -0.192 | 0.412 | 0.572 | 0.901 | FALSE | FALSE | FALSE |
| **GLRX** | Glutaredoxin-1 | 0.197 | 1.11 | -0.912 | 0.89 | 0.401 | 0.47 | FALSE | FALSE | FALSE |
| **GLRX3** | Glutaredoxin-3 | -0.444 | -0.613 | 0.17 | 0.674 | 0.608 | 0.888 | FALSE | FALSE | FALSE |
| **GLUL** | Glutamine synthetase | -0.317 | -0.342 | 0.0251 | 0.864 | 0.87 | 0.988 | FALSE | FALSE | FALSE |
| **GM2A** | GM2 ganglioside activator | 3.64 | 0.383 | 3.26 | 0.0244 | 0.866 | 0.076 | TRUE | FALSE | FALSE |
| **GMFG** | Glia maturation factor gamma | -3.56 | -2.7 | -0.861 | 0.0551 | 0.257 | 0.703 | FALSE | FALSE | FALSE |
| **GMPR2** | GMP reductase 2 | 0.158 | 2.53 | -2.37 | 0.917 | 0.236 | 0.17 | FALSE | FALSE | FALSE |

| | | | | | | | | | | |
|---|---|---|---|---|---|---|---|---|---|---|
| **GNA11** | Guanine nucleotide-binding protein subunit alpha-11 | 1.18 | -0.561 | 1.74 | 0.561 | 0.837 | 0.404 | FALSE | FALSE | FALSE |
| **GNA13** | G protein subunit alpha 13 | 0.731 | -1.37 | 2.1 | 0.668 | 0.45 | 0.229 | FALSE | FALSE | FALSE |
| **GNAI1** | Guanine nucleotide-binding protein G(i) subunit alpha-1 | -0.376 | 0.0566 | -0.433 | 0.74 | 0.967 | 0.738 | FALSE | FALSE | FALSE |
| **GNAI2** | G protein subunit alpha i2 | 0.301 | -0.899 | 1.2 | 0.782 | 0.432 | 0.277 | FALSE | FALSE | FALSE |
| **GNAI3** | G protein subunit alpha i3 | 1.56 | -1.31 | 2.87 | 0.257 | 0.407 | 0.0697 | FALSE | FALSE | FALSE |
| **GNAQ** | G protein subunit alpha q | 5.55 | 0.081 | 5.47 | 0.00972 | 0.978 | 0.0254 | TRUE | FALSE | TRUE |
| **GNAS** | Guanine nucleotide-binding protein G(s) subunit alpha isoforms short | -0.481 | -0.956 | 0.475 | 0.714 | 0.512 | 0.755 | FALSE | FALSE | FALSE |
| **GNB1** | Guanine nucleotide-binding protein G(I)/G(S)/G(T) subunit beta-1 | 1.28 | -0.443 | 1.72 | 0.243 | 0.751 | 0.159 | FALSE | FALSE | FALSE |
| **GNB2** | Guanine nucleotide-binding protein G(I)/G(S)/G(T) subunit beta-2 | 1.08 | -0.394 | 1.47 | 0.341 | 0.788 | 0.229 | FALSE | FALSE | FALSE |
| **GNB4** | G protein subunit beta 4 | -0.0526 | 0.4 | -0.452 | 0.959 | 0.755 | 0.706 | FALSE | FALSE | FALSE |
| **GNG10** | Guanine nucleotide-binding protein subunit gamma | -0.101 | -0.629 | 0.529 | 0.927 | 0.644 | 0.684 | FALSE | FALSE | FALSE |

| | | | | | | | | | | |
|---|---|---|---|---|---|---|---|---|---|---|
| **GNG12** | Guanine nucleotide-binding protein G(I)/G(S)/G(O) subunit gamma-12 | 2.63 | 1.09 | 1.54 | 0.068 | 0.524 | 0.327 | FALSE | FALSE | FALSE |
| **GNG5** | Guanine nucleotide-binding protein G(I)/G(S)/G(O) subunit gamma-5 | 1.54 | -1.21 | 2.76 | 0.343 | 0.513 | 0.123 | FALSE | FALSE | FALSE |
| **GOLGA3** | Golgin A3 | -2.51 | -2.14 | -0.372 | 0.0221 | 0.186 | 0.789 | TRUE | FALSE | FALSE |
| **GOLGA4** | Golgin A4 | 1.18 | 3.23 | -2.05 | 0.568 | 0.236 | 0.334 | FALSE | FALSE | FALSE |
| **GOLM1** | Golgi membrane protein 1 | -1.24 | -0.691 | -0.552 | 0.559 | 0.8 | 0.838 | FALSE | FALSE | FALSE |
| **GP2** | Glycoprotein 2 | 3.63 | 3.42 | 0.201 | 0.0058 | 0.054 | 0.894 | TRUE | FALSE | FALSE |
| **GPC1** | Glypican-1 | -3.69 | -2.55 | -1.14 | 0.176 | 0.416 | 0.73 | FALSE | FALSE | FALSE |
| **GPHN** | Gephyrin | 2.46 | 0.245 | 2.21 | 0.189 | 0.933 | 0.277 | FALSE | FALSE | FALSE |
| **GPLD1** | Phosphatidylinositol-glycan-specific phospholipase D | 0.39 | 1.77 | -1.38 | 0.715 | 0.219 | 0.222 | FALSE | FALSE | FALSE |
| **GPM6B** | Glycoprotein M6B | -1.77 | -0.815 | -0.95 | 0.0951 | 0.517 | 0.411 | FALSE | FALSE | FALSE |
| **GPRC5C** | G-protein-coupled receptor family C group 5 member C | 4.2 | 0.173 | 4.02 | 0.0479 | 0.956 | 0.0889 | TRUE | FALSE | FALSE |
| **GPX1** | Glutathione peroxidase 1 | -0.142 | -1.4 | 1.25 | 0.907 | 0.329 | 0.331 | FALSE | FALSE | FALSE |
| **GRB2** | Growth factor receptor-bound protein 2 | -0.436 | -0.0057 | -0.43 | 0.688 | 0.996 | 0.73 | FALSE | FALSE | FALSE |
| **GRK6** | G protein-coupled receptor kinase | -0.879 | -1.22 | 0.343 | 0.427 | 0.344 | 0.814 | FALSE | FALSE | FALSE |
| **GRP** | Gastrin-releasing peptide | -2.95 | -2.55 | -0.405 | 0.0873 | 0.256 | 0.856 | FALSE | FALSE | FALSE |

| | | | | | | | | | | |
|---|---|---|---|---|---|---|---|---|---|---|
| **GSPT1** | G1 to S phase transition 1 | -1.13 | 1.52 | -2.66 | 0.486 | 0.403 | 0.139 | FALSE | FALSE | FALSE |
| **GSR** | Glutathione reductase | -3.38 | -1.74 | -1.64 | 0.0548 | 0.393 | 0.386 | FALSE | FALSE | FALSE |
| **GSTP1** | Glutathione S-transferase | 0.899 | -1.5 | 2.4 | 0.422 | 0.268 | 0.0624 | FALSE | FALSE | FALSE |
| **GTPBP1** | GTP-binding protein 1 | -0.682 | 0.259 | -0.941 | 0.486 | 0.851 | 0.359 | FALSE | FALSE | FALSE |
| **GUSB** | Beta-glucuronidase | 0.158 | 1.28 | -1.12 | 0.895 | 0.31 | 0.324 | FALSE | FALSE | FALSE |
| **GYG1** | Glycogenin 1 | -1.02 | -0.606 | -0.417 | 0.422 | 0.69 | 0.798 | FALSE | FALSE | FALSE |
| **GYS1** | Glycogen [starch] synthase muscle | 3.02 | 1.17 | 1.85 | 0.0538 | 0.524 | 0.277 | FALSE | FALSE | FALSE |
| **H1-0** | Histone H1.0 | 0.503 | -0.176 | 0.68 | 0.785 | 0.947 | 0.744 | FALSE | FALSE | FALSE |
| **H1-1** | Histone H1.1 | -0.774 | -0.458 | -0.316 | 0.613 | 0.816 | 0.858 | FALSE | FALSE | FALSE |
| **H1-10** | H1.10 linker histone | 3 | 1.85 | 1.14 | 0.017 | 0.253 | 0.39 | TRUE | FALSE | FALSE |
| **H1-2** | Histone H1.2 | -2.8 | -0.634 | -2.17 | 0.00556 | 0.574 | 0.0435 | TRUE | FALSE | TRUE |
| **H2AC21** | Histone H2A | -4.09 | -1.9 | -2.19 | 0.00217 | 0.236 | 0.0919 | TRUE | FALSE | FALSE |
| **H2AJ** | Histone H2A.J | -5.54 | -3.76 | -1.78 | 0.00673 | 0.186 | 0.386 | TRUE | FALSE | FALSE |
| **H2BC12** | Histone H2B | -6.21 | -3.54 | -2.66 | 0.000411 | 0.0786 | 0.0873 | TRUE | FALSE | FALSE |
| **H3-4** | Histone H3.1 | -5.98 | -4.44 | -1.55 | 0.00839 | 0.173 | 0.522 | TRUE | FALSE | FALSE |
| **H4C9** | Histone H4 | -4.29 | -2.88 | -1.41 | 0.00833 | 0.197 | 0.395 | TRUE | FALSE | FALSE |
| **HABP2** | Hyaluronan-binding protein 2 | -1.93 | -2.18 | 0.247 | 0.0485 | 0.13 | 0.846 | TRUE | FALSE | FALSE |
| **HCK** | Tyrosine-protein kinase | -0.811 | -0.542 | -0.27 | 0.546 | 0.742 | 0.865 | FALSE | FALSE | FALSE |
| **HDLBP** | High density lipoprotein binding protein | -0.0676 | 1.61 | -1.67 | 0.954 | 0.259 | 0.185 | FALSE | FALSE | FALSE |
| **HEBP1** | Heme-binding protein 1 | -0.0014 | 0.38 | -0.382 | 0.999 | 0.738 | 0.726 | FALSE | FALSE | FALSE |

| | | | | | | | | | | |
|---|---|---|---|---|---|---|---|---|---|---|
| **HECTD3** | HECT domain E3 ubiquitin protein ligase 3 | -1.6 | -0.813 | -0.791 | 0.302 | 0.669 | 0.663 | FALSE | FALSE | FALSE |
| **HEG1** | Heart development protein with EGF like domains 1 | 1.24 | -0.292 | 1.53 | 0.457 | 0.903 | 0.386 | FALSE | FALSE | FALSE |
| **HGS** | Hepatocyte growth factor-regulated tyrosine kinase substrate | -1.42 | -0.419 | -1 | 0.26 | 0.804 | 0.469 | FALSE | FALSE | FALSE |
| **HK1** | Hexokinase | 0.511 | 0.644 | -0.133 | 0.642 | 0.608 | 0.915 | FALSE | FALSE | FALSE |
| **HK3** | Hexokinase | -4.64 | -3.56 | -1.09 | 0.0343 | 0.236 | 0.676 | TRUE | FALSE | FALSE |
| **HNRNPA0** | Heteroous nuclear ribonucleoprotein A0 | 0.338 | 0.284 | 0.0545 | 0.751 | 0.84 | 0.969 | FALSE | FALSE | FALSE |
| **HNRNPA1** | Heterogeneous nuclear ribonucleoprotein A1 | 1.98 | 0.245 | 1.73 | 0.149 | 0.903 | 0.246 | FALSE | FALSE | FALSE |
| **HNRNPA2B1** | Heterogeneous nuclear ribonucleoproteins A2/B1 | 0.813 | 1.28 | -0.467 | 0.679 | 0.565 | 0.846 | FALSE | FALSE | FALSE |
| **HNRNPA3** | Heteroous nuclear ribonucleoprotein A3 | 1.86 | 1.93 | -0.0663 | 0.0695 | 0.197 | 0.964 | FALSE | FALSE | FALSE |
| **HNRNPAB** | Heterogeneous nuclear ribonucleoprotein A/B | 3.95 | 1.9 | 2.05 | 0.00838 | 0.275 | 0.197 | TRUE | FALSE | FALSE |

| | | | | | | | | | | |
|---|---|---|---|---|---|---|---|---|---|---|
| **HNRNPD** | Heterogeneous nuclear ribonucleoprotein D | 1.7 | 0.306 | 1.39 | 0.117 | 0.843 | 0.243 | FALSE | FALSE | FALSE |
| **HNRNPF** | Heterogeneous nuclear ribonucleoprotein F | 2.41 | 1.95 | 0.46 | 0.0482 | 0.236 | 0.76 | TRUE | FALSE | FALSE |
| **HNRNPH1** | Heteroous nuclear ribonucleoprotein H1 | 4.63 | 1.9 | 2.73 | 0.00736 | 0.329 | 0.139 | TRUE | FALSE | FALSE |
| **HNRNPK** | Heterogeneous nuclear ribonucleoprotein K | 4.45 | 1.53 | 2.93 | 0.00331 | 0.339 | 0.0624 | TRUE | FALSE | FALSE |
| **HNRNPL** | Heteroous nuclear ribonucleoprotein L | 3.49 | 2.19 | 1.3 | 0.0227 | 0.257 | 0.428 | TRUE | FALSE | FALSE |
| **HNRNPM** | Heteroous nuclear ribonucleoprotein M | 1.74 | 2.88 | -1.14 | 0.209 | 0.16 | 0.445 | FALSE | FALSE | FALSE |
| **HNRNPR** | HNRNPR protein | 0.481 | 0.84 | -0.359 | 0.696 | 0.543 | 0.815 | FALSE | FALSE | FALSE |
| **HNRNPU** | Heterogeneous nuclear ribonucleoprotein U (Scaffold attachment factor A) | 6.74 | 3.07 | 3.66 | 0.00223 | 0.236 | 0.0889 | TRUE | FALSE | FALSE |
| **HNRNPUL1** | Heteroous nuclear ribonucleoprotein U like 1 | -0.915 | -0.0103 | -0.905 | 0.362 | 0.995 | 0.389 | FALSE | FALSE | FALSE |
| **HNRNPUL2** | Heteroous nuclear ribonucleoprotein U like 2 | 4.27 | 2.01 | 2.27 | 0.0113 | 0.312 | 0.218 | TRUE | FALSE | FALSE |

| | | | | | | | | | | |
|---|---|---|---|---|---|---|---|---|---|---|
| **HNRPC** | Heterogeneous nuclear ribonucleoprotein C (C1/C2) | 1.48 | 2.39 | -0.905 | 0.3 | 0.232 | 0.571 | FALSE | FALSE | FALSE |
| **HP** | Haptoglobin | -8.71 | -6.47 | -2.24 | 4.91E-05 | 0.00231 | 0.132 | TRUE | TRUE | FALSE |
| **HPCAL1** | Hippocalcin-like protein 1 | -0.0197 | 0.552 | -0.572 | 0.992 | 0.822 | 0.807 | FALSE | FALSE | FALSE |
| **HPRT1** | Hypoxanthine-guanine phosphoribosyltransferase | 0.55 | 0.0771 | 0.473 | 0.696 | 0.967 | 0.779 | FALSE | FALSE | FALSE |
| **HRAS** | HRas proto-onco GTPase | -1.1 | 0.189 | -1.29 | 0.375 | 0.919 | 0.327 | FALSE | FALSE | FALSE |
| **HRG** | Histidine-rich glycoprotein | 1.43 | -0.746 | 2.18 | 0.389 | 0.717 | 0.229 | FALSE | FALSE | FALSE |
| **HSD17B11** | Hydroxysteroid (17-beta) dehydrogenase 11 | -1.01 | -0.737 | -0.275 | 0.422 | 0.633 | 0.857 | FALSE | FALSE | FALSE |
| **HSD17B12** | Very-long-chain 3-oxoacyl-CoA reductase | 0.0402 | -0.447 | 0.488 | 0.968 | 0.69 | 0.655 | FALSE | FALSE | FALSE |
| **HSD17B13** | Hydroxysteroid (17-beta) dehydrogenase 13 | 0.0581 | 0.875 | -0.817 | 0.957 | 0.468 | 0.472 | FALSE | FALSE | FALSE |
| **HSP90AA1** | Heat shock protein HSP 90-alpha | 0.466 | -1.06 | 1.52 | 0.739 | 0.484 | 0.277 | FALSE | FALSE | FALSE |
| **HSP90AB1** | Heat shock protein HSP 90-beta | 1.73 | -0.725 | 2.45 | 0.0904 | 0.561 | 0.039 | FALSE | FALSE | TRUE |
| **HSP90B1** | Endoplasmin | 4 | 2.1 | 1.9 | 0.0158 | 0.285 | 0.282 | TRUE | FALSE | FALSE |
| **HSPA1A** | Heat shock 70 kDa protein 1A | 1.82 | 0.342 | 1.47 | 0.117 | 0.835 | 0.248 | FALSE | FALSE | FALSE |

| | | | | | | | | | | |
|---|---|---|---|---|---|---|---|---|---|---|
| **HSPA2** | Heat shock 70kDa protein 1A | -0.667 | 0.606 | -1.27 | 0.51 | 0.621 | 0.241 | FALSE | FALSE | FALSE |
| **HSPA4** | Heat shock 70 kDa protein 4 | 1.37 | -0.131 | 1.5 | 0.242 | 0.94 | 0.237 | FALSE | FALSE | FALSE |
| **HSPA5** | Endoplasmic reticulum chaperone BiP | 4.16 | 2.72 | 1.44 | 0.0767 | 0.335 | 0.592 | FALSE | FALSE | FALSE |
| **HSPA8** | Heat shock cognate 71 kDa protein | 0.766 | -0.291 | 1.06 | 0.434 | 0.83 | 0.318 | FALSE | FALSE | FALSE |
| **HSPB1** | Heat shock protein beta-1 | 2.29 | -0.146 | 2.43 | 0.0548 | 0.936 | 0.0747 | FALSE | FALSE | FALSE |
| **HSPG2** | Heparan sulfate proteoglycan 2 | -0.831 | -0.51 | -0.321 | 0.428 | 0.69 | 0.815 | FALSE | FALSE | FALSE |
| **HSPH1** | Heat shock protein 105 kDa | -0.943 | -0.532 | -0.411 | 0.369 | 0.676 | 0.744 | FALSE | FALSE | FALSE |
| **HTRA1** | Serine peptidase HtrA1 | -1.91 | -6.82 | 4.91 | 0.383 | 0.0261 | 0.0489 | FALSE | TRUE | TRUE |
| **HUWE1** | HECT-type E3 ubiquitin transferase | 3.5 | 1.42 | 2.08 | 0.0278 | 0.432 | 0.232 | TRUE | FALSE | FALSE |
| **HYAL2** | Hyaluronidase-2 | -1.42 | -1.71 | 0.293 | 0.223 | 0.256 | 0.846 | FALSE | FALSE | FALSE |
| **HYOU1** | Hypoxia up-regulated 1 | 1.78 | 0.856 | 0.927 | 0.206 | 0.621 | 0.561 | FALSE | FALSE | FALSE |
| **IARS1** | Isoleucine--tRNA ligase | 0.115 | 0.491 | -0.376 | 0.97 | 0.918 | 0.919 | FALSE | FALSE | FALSE |
| **ICAM1** | Intercellular adhesion molecule 1 | -1.65 | -1.48 | -0.167 | 0.226 | 0.35 | 0.918 | FALSE | FALSE | FALSE |
| **ICAM3** | Intercellular adhesion molecule 3 | -2.57 | -1.61 | -0.958 | 0.0812 | 0.357 | 0.57 | FALSE | FALSE | FALSE |

| | | | | | | | | | | |
|---|---|---|---|---|---|---|---|---|---|---|
| **ICOSLG** | Ig-like domain-containing protein | -1.04 | -1.13 | 0.0907 | 0.488 | 0.512 | 0.965 | FALSE | FALSE | FALSE |
| **IDE** | Insulin-degrading enzyme | 1.1 | 0.751 | 0.349 | 0.344 | 0.585 | 0.815 | FALSE | FALSE | FALSE |
| **IDH1** | Isocitrate dehydrogenase [NADP] | 1.53 | -1.81 | 3.33 | 0.183 | 0.236 | 0.0171 | FALSE | FALSE | TRUE |
| **IDUA** | Alpha-L-iduronidase | -1.76 | -0.986 | -0.777 | 0.458 | 0.744 | 0.799 | FALSE | FALSE | FALSE |
| **IFIT1** | Interferon induced protein with tetratricopeptide repeats 1 | 2.31 | 2.52 | -0.204 | 0.219 | 0.271 | 0.924 | FALSE | FALSE | FALSE |
| **IFT52** | Intraflagellar transport 52 | -1.96 | 0.0696 | -2.03 | 0.135 | 0.967 | 0.17 | FALSE | FALSE | FALSE |
| **IGF2BP2** | Insulin like growth factor 2 mRNA binding protein 2 | -0.652 | -0.401 | -0.252 | 0.537 | 0.76 | 0.848 | FALSE | FALSE | FALSE |
| **IGF2R** | Cation-independent mannose-6-phosphate receptor | -1.33 | -0.742 | -0.585 | 0.343 | 0.66 | 0.725 | FALSE | FALSE | FALSE |
| **IGSF5** | Immunoglobulin superfamily member 5 | -0.81 | 0.895 | -1.7 | 0.631 | 0.648 | 0.326 | FALSE | FALSE | FALSE |
| **IKBKB** | Inhibitor of nuclear factor kappa-B kinase subunit beta | -1.28 | -0.796 | -0.485 | 0.209 | 0.503 | 0.686 | FALSE | FALSE | FALSE |
| **IL1RAP** | Interleukin 1 receptor accessory protein | -1.56 | -0.956 | -0.606 | 0.207 | 0.512 | 0.677 | FALSE | FALSE | FALSE |
| **IL1RN** | Interleukin-1 | 0.158 | -0.795 | 0.954 | 0.892 | 0.482 | 0.359 | FALSE | FALSE | FALSE |

| | | | | | | | | | | |
|---|---|---|---|---|---|---|---|---|---|---|
| **ILF2** | Interleukin enhancer binding factor 2 | 1.17 | 1.28 | -0.101 | 0.488 | 0.512 | 0.965 | FALSE | FALSE | FALSE |
| **ILF3** | Interleukin enhancer binding factor 3 | 3.77 | 2.88 | 0.886 | 0.0598 | 0.259 | 0.718 | FALSE | FALSE | FALSE |
| **ILK** | Integrin-linked protein kinase | -0.449 | 0.797 | -1.25 | 0.731 | 0.591 | 0.342 | FALSE | FALSE | FALSE |
| **ILVBL** | 2-hydroxyacyl-CoA lyase 2 | 1.04 | 0.221 | 0.823 | 0.373 | 0.895 | 0.52 | FALSE | FALSE | FALSE |
| **IMPDH2** | Inosine-5-monophosphate dehydrogenase 2 | -1.17 | 0.369 | -1.54 | 0.381 | 0.843 | 0.281 | FALSE | FALSE | FALSE |
| **IPO4** | Importin 4 | 1.34 | 1.13 | 0.213 | 0.3 | 0.439 | 0.893 | FALSE | FALSE | FALSE |
| **IPO5** | Importin 5 | -0.0925 | -0.349 | 0.256 | 0.924 | 0.788 | 0.842 | FALSE | FALSE | FALSE |
| **IQGAP1** | IQ motif containing GTPase activating protein 1 | 2.34 | -0.983 | 3.33 | 0.249 | 0.69 | 0.145 | FALSE | FALSE | FALSE |
| **IQGAP2** | IQ motif containing GTPase activating protein 2 | 1.92 | -0.686 | 2.61 | 0.457 | 0.852 | 0.344 | FALSE | FALSE | FALSE |
| **IST1** | IST1 homolog | 1.21 | -0.975 | 2.19 | 0.458 | 0.628 | 0.227 | FALSE | FALSE | FALSE |
| **ITCH** | E3 ubiquitin-protein ligase | -0.87 | -0.536 | -0.334 | 0.427 | 0.69 | 0.815 | FALSE | FALSE | FALSE |
| **ITFG1** | Integrin alpha FG-GAP repeat containing 1 | -2.19 | -1.13 | -1.06 | 0.281 | 0.648 | 0.654 | FALSE | FALSE | FALSE |
| **ITGA2** | Integrin alpha-2 | -0.918 | 1.19 | -2.11 | 0.625 | 0.576 | 0.277 | FALSE | FALSE | FALSE |
| **ITGA3** | Integrin alpha-3 | 2.32 | 0.36 | 1.96 | 0.152 | 0.874 | 0.27 | FALSE | FALSE | FALSE |
| **ITGA6** | Integrin subunit alpha 6 | 2.81 | 2.16 | 0.646 | 0.0285 | 0.232 | 0.668 | TRUE | FALSE | FALSE |

| | | | | | | | | | | |
|---|---|---|---|---|---|---|---|---|---|---|
| **ITGAL** | Integrin alpha-L | 0.212 | 1.02 | -0.803 | 0.873 | 0.409 | 0.497 | FALSE | FALSE | FALSE |
| **ITGAM** | Integrin subunit alpha M | -4.86 | -2.79 | -2.07 | 0.00597 | 0.232 | 0.248 | TRUE | FALSE | FALSE |
| **ITGAV** | Integrin alpha-V | 0.31 | 0.865 | -0.555 | 0.805 | 0.512 | 0.677 | FALSE | FALSE | FALSE |
| **ITGAX** | VWFA domain-containing protein | -1.68 | -1.46 | -0.213 | 0.0782 | 0.25 | 0.857 | FALSE | FALSE | FALSE |
| **ITGB1** | Integrin beta-1 | 3.4 | 0.902 | 2.5 | 0.0116 | 0.569 | 0.0889 | TRUE | FALSE | FALSE |
| **ITGB1BP1** | Integrin beta-1-binding protein 1 | -1.01 | -1.05 | 0.0413 | 0.373 | 0.407 | 0.977 | FALSE | FALSE | FALSE |
| **ITGB2** | Integrin beta-2 | -4.71 | -3.07 | -1.64 | 0.0116 | 0.232 | 0.403 | TRUE | FALSE | FALSE |
| **ITGB3** | Integrin beta | -0.832 | 2.18 | -3.02 | 0.716 | 0.384 | 0.21 | FALSE | FALSE | FALSE |
| **ITGB4** | Integrin beta | 0.602 | 1.32 | -0.721 | 0.851 | 0.69 | 0.841 | FALSE | FALSE | FALSE |
| **ITGB6** | Integrin beta-6 | 0.876 | 0.948 | -0.0715 | 0.615 | 0.642 | 0.975 | FALSE | FALSE | FALSE |
| **ITM2B** | Integral membrane protein 2 | -2.16 | -1.34 | -0.816 | 0.12 | 0.401 | 0.617 | FALSE | FALSE | FALSE |
| **JAKMIP1** | Janus kinase and microtubule-interacting protein 1 | 0.151 | 0.199 | -0.0482 | 0.892 | 0.883 | 0.974 | FALSE | FALSE | FALSE |
| **JCHAIN** | Immunoglobulin J chain | 1.37 | -0.0231 | 1.4 | 0.362 | 0.993 | 0.381 | FALSE | FALSE | FALSE |
| **JSP.1** | MHC class I antigen | 0.957 | -0.616 | 1.57 | 0.42 | 0.67 | 0.228 | FALSE | FALSE | FALSE |
| **JUP** | Junction plakoglobin | 0.291 | 0.869 | -0.578 | 0.805 | 0.48 | 0.642 | FALSE | FALSE | FALSE |
| **KARS1** | Lysine--tRNA ligase | 4.51 | 1.95 | 2.55 | 0.0116 | 0.342 | 0.191 | TRUE | FALSE | FALSE |
| **KCTD12** | Potassium channel tetramerization domain containing 12 | -0.187 | 1.05 | -1.24 | 0.915 | 0.615 | 0.513 | FALSE | FALSE | FALSE |

| | | | | | | | | | | |
|---|---|---|---|---|---|---|---|---|---|---|
| **KHDRBS1** | KH RNA binding domain containing signal transduction associated 1 | 4.09 | 1.64 | 2.45 | 0.00546 | 0.316 | 0.107 | TRUE | FALSE | FALSE |
| **KHSRP** | KH-type splicing regulatory protein | 1.71 | 1.53 | 0.179 | 0.152 | 0.285 | 0.901 | FALSE | FALSE | FALSE |
| **KIAA1549** | KIAA1549 | -1.53 | -0.607 | -0.92 | 0.104 | 0.599 | 0.363 | FALSE | FALSE | FALSE |
| **KIF13B** | Kinesin family member 13B | -0.633 | 0.181 | -0.815 | 0.613 | 0.919 | 0.544 | FALSE | FALSE | FALSE |
| **KIF21A** | Kinesin family member 21A | 0.000707 | -0.592 | 0.592 | 0.999 | 0.61 | 0.584 | FALSE | FALSE | FALSE |
| **KIF2A** | Kinesin-like protein KIF2A | -1.14 | -0.787 | -0.353 | 0.324 | 0.565 | 0.815 | FALSE | FALSE | FALSE |
| **KIF5B** | Kinesin-like protein | 1.74 | 0.542 | 1.19 | 0.301 | 0.807 | 0.52 | FALSE | FALSE | FALSE |
| **KLC4** | Kinesin light chain 4 | -1.8 | -1.08 | -0.716 | 0.148 | 0.446 | 0.619 | FALSE | FALSE | FALSE |
| **KPNB1** | Karyopherin subunit beta 1 | 0.185 | -0.714 | 0.899 | 0.9 | 0.673 | 0.562 | FALSE | FALSE | FALSE |
| **KRAS** | KRAS proto-onco GTPase | 1.17 | -0.265 | 1.43 | 0.376 | 0.883 | 0.31 | FALSE | FALSE | FALSE |
| **KRIT1** | KRIT1 protein | -0.676 | -0.945 | 0.268 | 0.708 | 0.652 | 0.898 | FALSE | FALSE | FALSE |
| **KRT15** | KRT15 protein | 0.158 | 0.365 | -0.207 | 0.905 | 0.838 | 0.895 | FALSE | FALSE | FALSE |
| **KRT18** | Keratin 18 | 2.53 | 2.97 | -0.439 | 0.262 | 0.279 | 0.875 | FALSE | FALSE | FALSE |
| **KRT19** | Keratin type I cytoskeletal 19 | 6.5 | 1.27 | 5.23 | 0.000813 | 0.491 | 0.00835 | TRUE | FALSE | TRUE |
| **KRT33A** | KRT33A protein | 0.585 | 0.339 | 0.247 | 0.594 | 0.807 | 0.853 | FALSE | FALSE | FALSE |
| **KRT34** | Keratin 34 | -0.736 | -0.982 | 0.247 | 0.501 | 0.417 | 0.856 | FALSE | FALSE | FALSE |
| **KRT4** | KRT4 protein | -3.46 | -4.14 | 0.672 | 0.00687 | 0.0191 | 0.632 | TRUE | TRUE | FALSE |
| **KRT7** | Keratin type II cytoskeletal 7 | 2.21 | 0.384 | 1.83 | 0.154 | 0.862 | 0.277 | FALSE | FALSE | FALSE |
| **KRT8** | Keratin type II cytoskeletal 8 | 0.853 | 0.631 | 0.222 | 0.474 | 0.661 | 0.876 | FALSE | FALSE | FALSE |

| | | | | | | | | | | |
|---|---|---|---|---|---|---|---|---|---|---|
| **KRT81** | Keratin type II cytoskeletal Hb1 | 0.503 | 0.256 | 0.247 | 0.689 | 0.876 | 0.865 | FALSE | FALSE | FALSE |
| **KRT83** | Keratin type II cytoskeletal Hb3 | 3.17 | 5.59 | -2.41 | 0.3 | 0.201 | 0.469 | FALSE | FALSE | FALSE |
| **KRT85** | Keratin 85 | 0.724 | 0.478 | 0.247 | 0.46 | 0.69 | 0.846 | FALSE | FALSE | FALSE |
| **KTN1** | Kinectin 1 | 1.81 | 2 | -0.19 | 0.285 | 0.323 | 0.921 | FALSE | FALSE | FALSE |
| **LAMB3** | Laminin subunit beta 3 | 0.158 | 1.13 | -0.969 | 0.905 | 0.438 | 0.488 | FALSE | FALSE | FALSE |
| **LAMC1** | Laminin subunit gamma 1 | -0.457 | 0.918 | -1.37 | 0.689 | 0.464 | 0.245 | FALSE | FALSE | FALSE |
| **LAMC2** | Laminin subunit gamma 2 | 0.136 | -1.46 | 1.6 | 0.905 | 0.268 | 0.185 | FALSE | FALSE | FALSE |
| **LAMP1** | Lysosome-associated membrane glycoprotein 1 | 0.117 | 1.23 | -1.11 | 0.922 | 0.373 | 0.386 | FALSE | FALSE | FALSE |
| **LAMP2** | Lysosomal-associated membrane protein 2 | 3.81 | 2.1 | 1.71 | 0.0125 | 0.261 | 0.29 | TRUE | FALSE | FALSE |
| **LAMTOR3** | Ragulator complex protein LAMTOR3 | -0.634 | -0.459 | -0.175 | 0.607 | 0.756 | 0.901 | FALSE | FALSE | FALSE |
| **LANCL1** | Glutathione S-transferase LANCL1 | -1.61 | -0.669 | -0.939 | 0.0878 | 0.561 | 0.359 | FALSE | FALSE | FALSE |
| **LANCL2** | LANCL2 protein | -0.724 | -0.541 | -0.184 | 0.474 | 0.66 | 0.879 | FALSE | FALSE | FALSE |
| **LAP3** | Cytosol aminopeptidase | -1.12 | -1.32 | 0.197 | 0.609 | 0.599 | 0.934 | FALSE | FALSE | FALSE |
| **LARP1** | La ribonucleoprotein 1 translational regulator | -0.714 | -0.328 | -0.386 | 0.551 | 0.839 | 0.795 | FALSE | FALSE | FALSE |

| | | | | | | | | | | |
|---|---|---|---|---|---|---|---|---|---|---|
| **LARP7** | La-related protein 7 | -0.799 | -0.572 | -0.227 | 0.452 | 0.66 | 0.859 | FALSE | FALSE | FALSE |
| **LARS1** | Leucine--tRNA ligase | -0.303 | 1.09 | -1.39 | 0.837 | 0.447 | 0.309 | FALSE | FALSE | FALSE |
| **LASP1** | LIM and SH3 domain protein 1 | 2.04 | 0.804 | 1.24 | 0.0548 | 0.52 | 0.282 | FALSE | FALSE | FALSE |
| **LCAT** | Lecithin-cholesterol acyltransferase | 0.158 | 1.07 | -0.907 | 0.907 | 0.488 | 0.547 | FALSE | FALSE | FALSE |
| **LCN2** | Lipocalin 2 | -8.22 | -6.37 | -1.85 | 0.0003 | 0.0129 | 0.327 | TRUE | TRUE | FALSE |
| **LCP1** | Lymphocyte cytosolic protein 1 | -7.56 | -5.34 | -2.22 | 0.000378 | 0.0244 | 0.228 | TRUE | TRUE | FALSE |
| **LDHA** | L-lactate dehydrogenase A chain | 0.87 | -0.496 | 1.37 | 0.676 | 0.858 | 0.529 | FALSE | FALSE | FALSE |
| **LDHB** | L-lactate dehydrogenase | -1.9 | -0.403 | -1.5 | 0.169 | 0.835 | 0.316 | FALSE | FALSE | FALSE |
| **LGALS1** | Galectin-1 | 0.864 | -0.386 | 1.25 | 0.688 | 0.896 | 0.586 | FALSE | FALSE | FALSE |
| **LGALS3** | Galectin | 0.34 | -1.94 | 2.28 | 0.801 | 0.236 | 0.0931 | FALSE | FALSE | FALSE |
| **LGALS3BP** | Galectin-3-binding protein | 0.879 | -1.25 | 2.12 | 0.684 | 0.617 | 0.331 | FALSE | FALSE | FALSE |
| **LGALS7** | Galectin | -2.12 | -1.6 | -0.516 | 0.0392 | 0.239 | 0.668 | TRUE | FALSE | FALSE |
| **LGALS9** | Galectin | -0.551 | 0.659 | -1.21 | 0.654 | 0.642 | 0.329 | FALSE | FALSE | FALSE |
| **LGMN** | Legumain | 0.158 | 0.814 | -0.656 | 0.892 | 0.465 | 0.549 | FALSE | FALSE | FALSE |
| **LIMK2** | LIM domain kinase 2 | -2.57 | -2.81 | 0.247 | 0.00839 | 0.0326 | 0.841 | TRUE | TRUE | FALSE |
| **LIMS1** | LIM and senescent cell antigen-like-containing domain protein | 0.0394 | 0.116 | -0.0768 | 0.97 | 0.938 | 0.95 | FALSE | FALSE | FALSE |
| **LIN7C** | Protein lin-7 homolog C | 1.42 | 0.331 | 1.09 | 0.183 | 0.822 | 0.342 | FALSE | FALSE | FALSE |

| | | | | | | | | | | |
|---|---|---|---|---|---|---|---|---|---|---|
| **LLGL2** | LLGL scribble cell polarity complex component 2 | 0.842 | 1.86 | -1.01 | 0.593 | 0.299 | 0.548 | FALSE | FALSE | FALSE |
| **LMAN1** | Lectin mannose binding 1 | 0.956 | 1.59 | -0.632 | 0.544 | 0.363 | 0.73 | FALSE | FALSE | FALSE |
| **LMAN2** | LMAN2 protein | 2.65 | 1.43 | 1.21 | 0.0609 | 0.388 | 0.428 | FALSE | FALSE | FALSE |
| **LMNA** | Lamin A/C | -0.317 | 0.168 | -0.484 | 0.846 | 0.936 | 0.782 | FALSE | FALSE | FALSE |
| **LOC100847119** | Uncharacterized protein | -1.6 | -0.0022 | -1.59 | 0.264 | 0.999 | 0.307 | FALSE | FALSE | FALSE |
| **LOC100850808** | WAP domain-containing protein | 0.719 | 0.278 | 0.441 | 0.781 | 0.938 | 0.879 | FALSE | FALSE | FALSE |
| **LOC104975830** | Uncharacterized protein | -1.07 | -0.981 | -0.0928 | 0.334 | 0.43 | 0.943 | FALSE | FALSE | FALSE |
| **LOC107131803** | SERPIN domain-containing protein | -10.4 | -5.32 | -5.03 | 0.000309 | 0.0947 | 0.0467 | TRUE | FALSE | TRUE |
| **LOC107132283** | GP41 domain-containing protein | -0.223 | 0.118 | -0.341 | 0.871 | 0.946 | 0.816 | FALSE | FALSE | FALSE |
| **LOC407171** | Uncharacterized protein | -0.903 | 0.183 | -1.09 | 0.435 | 0.918 | 0.381 | FALSE | FALSE | FALSE |
| **LOC505658** | Peptidase S1 domain-containing protein | -6.45 | -5.09 | -1.36 | 0.00101 | 0.0294 | 0.445 | TRUE | TRUE | FALSE |
| **LOC506828** | Uncharacterized protein | -2.31 | -1.68 | -0.637 | 0.302 | 0.519 | 0.825 | FALSE | FALSE | FALSE |
| **LOC509283** | RING-type domain-containing protein | 0.778 | 0.545 | 0.234 | 0.549 | 0.729 | 0.876 | FALSE | FALSE | FALSE |
| **LOC511531** | GB1/RHD3-type G domain-containing protein | 0.158 | 0.499 | -0.341 | 0.892 | 0.673 | 0.779 | FALSE | FALSE | FALSE |
| **LOC519132** | SERPIN domain-containing protein | -6.32 | -1.93 | -4.39 | 0.000908 | 0.316 | 0.0214 | TRUE | FALSE | TRUE |

| | | | | | | | | | | |
|---|---|---|---|---|---|---|---|---|---|---|
| **LOC521224** | Uncharacterized protein | 2.47 | -0.524 | 3 | 0.169 | 0.835 | 0.137 | FALSE | FALSE | FALSE |
| **LOC522479** | SERPIN domain-containing protein | 0.158 | 1.68 | -1.52 | 0.9 | 0.257 | 0.232 | FALSE | FALSE | FALSE |
| **LOC523130** | Uncharacterized protein | 1.91 | -0.353 | 2.26 | 0.0925 | 0.822 | 0.0814 | FALSE | FALSE | FALSE |
| **LOC528040** | Uncharacterized protein | 0.695 | 1.41 | -0.712 | 0.642 | 0.388 | 0.665 | FALSE | FALSE | FALSE |
| **LOC616254** | ICAM_N domain-containing protein | 0.125 | -0.221 | 0.347 | 0.9 | 0.862 | 0.756 | FALSE | FALSE | FALSE |
| **LOC616942** | Ig-like domain-containing protein | 0.399 | -0.163 | 0.562 | 0.74 | 0.927 | 0.672 | FALSE | FALSE | FALSE |
| **LOC784254** | Carbonic anhydrase | -0.379 | -2.27 | 1.89 | 0.892 | 0.404 | 0.469 | FALSE | FALSE | FALSE |
| **LOC786410** | Serpin family B member 4 | -2.64 | 0.295 | -2.93 | 0.0314 | 0.865 | 0.037 | TRUE | FALSE | TRUE |
| **LOC788112** | LOC788112 protein | -4.14 | -2.22 | -1.92 | 0.00433 | 0.232 | 0.193 | TRUE | FALSE | FALSE |
| **LRBA** | LPS responsive beige-like anchor protein | -1.22 | 0.148 | -1.37 | 0.654 | 0.967 | 0.647 | FALSE | FALSE | FALSE |
| **LRP2** | LDL receptor related protein 2 | 0.973 | -0.159 | 1.13 | 0.772 | 0.969 | 0.774 | FALSE | FALSE | FALSE |
| **LRRC47** | Leucine rich repeat containing 47 | -1.44 | 1.12 | -2.56 | 0.281 | 0.462 | 0.0866 | FALSE | FALSE | FALSE |
| **LRRC57** | LRRC57 protein | -0.793 | -1.32 | 0.524 | 0.572 | 0.393 | 0.75 | FALSE | FALSE | FALSE |
| **LRRFIP1** | LRR binding FLII interacting protein 1 | -1.8 | -1.41 | -0.391 | 0.0844 | 0.274 | 0.765 | FALSE | FALSE | FALSE |
| **LSR** | Lipolysis stimulated lipoprotein receptor | -0.977 | -0.319 | -0.657 | 0.376 | 0.834 | 0.592 | FALSE | FALSE | FALSE |

| | | | | | | | | | | |
|---|---|---|---|---|---|---|---|---|---|---|
| **LTA4H** | Leukotriene A(4) hydrolase | -0.284 | -0.559 | 0.275 | 0.819 | 0.679 | 0.846 | FALSE | FALSE | FALSE |
| **LTBP1** | Latent transforming growth factor beta binding protein 1 | -1.8 | -0.635 | -1.16 | 0.549 | 0.874 | 0.742 | FALSE | FALSE | FALSE |
| **LTF** | Lactotransferrin | 0.619 | 1.29 | -0.668 | 0.66 | 0.393 | 0.663 | FALSE | FALSE | FALSE |
| **LY6G6C** | Lymphocyte antigen 6 complex locus protein G6c | 0.17 | -2.24 | 2.41 | 0.905 | 0.235 | 0.113 | FALSE | FALSE | FALSE |
| **LY6G6E** | Lymphocyte antigen 6 complex locus G6E | 0.187 | 0.473 | -0.286 | 0.892 | 0.749 | 0.847 | FALSE | FALSE | FALSE |
| **LY96** | Lymphocyte antigen 96 | -0.705 | 0.0374 | -0.742 | 0.609 | 0.984 | 0.62 | FALSE | FALSE | FALSE |
| **LYN** | Tyrosine-protein kinase | -0.0962 | -0.484 | 0.388 | 0.934 | 0.741 | 0.793 | FALSE | FALSE | FALSE |
| **LYPLA1** | Palmitoyl-protein hydrolase | -1 | -0.291 | -0.71 | 0.512 | 0.893 | 0.691 | FALSE | FALSE | FALSE |
| **M6PR** | Cation-dependent mannose-6-phosphate receptor | -0.516 | 0.774 | -1.29 | 0.689 | 0.606 | 0.331 | FALSE | FALSE | FALSE |
| **MACF1** | Microtubule actin crosslinking factor 1 | 1.58 | 1.56 | 0.025 | 0.2 | 0.291 | 0.986 | FALSE | FALSE | FALSE |
| **MACROH2A1** | Core histone macro-H2A | 0.462 | 0.231 | 0.231 | 0.676 | 0.873 | 0.857 | FALSE | FALSE | FALSE |
| **MAL2** | Protein MAL2 | -0.342 | 0.404 | -0.746 | 0.746 | 0.749 | 0.489 | FALSE | FALSE | FALSE |
| **MAN2A2** | Alpha-mannosidase | -1.24 | -0.163 | -1.07 | 0.572 | 0.958 | 0.663 | FALSE | FALSE | FALSE |
| **MAP1S** | Microtubule-associated protein 1S | -1.44 | 0.27 | -1.71 | 0.315 | 0.896 | 0.27 | FALSE | FALSE | FALSE |
| **MAP2K1** | MAP2K1 protein | -1.06 | 0.144 | -1.2 | 0.362 | 0.935 | 0.329 | FALSE | FALSE | FALSE |

| | | | | | | | | | | |
|---|---|---|---|---|---|---|---|---|---|---|
| **MAP4** | Microtubule-associated protein | 3.77 | 3.18 | 0.593 | 0.0102 | 0.129 | 0.73 | TRUE | FALSE | FALSE |
| **MAP7** | Microtubule associated protein 7 | 1.74 | 2.11 | -0.372 | 0.379 | 0.357 | 0.876 | FALSE | FALSE | FALSE |
| **MAPK1** | Mitogen-activated protein kinase 1 | -1.15 | -1.33 | 0.178 | 0.345 | 0.342 | 0.901 | FALSE | FALSE | FALSE |
| **MARCKS** | Myristoylated alanine-rich C-kinase substrate | 1.15 | -0.204 | 1.35 | 0.4 | 0.921 | 0.351 | FALSE | FALSE | FALSE |
| **MARCKSL1** | MARCKS-related protein | 1.96 | -0.362 | 2.32 | 0.172 | 0.858 | 0.146 | FALSE | FALSE | FALSE |
| **MARS1** | Methionine-tRNA ligase cytoplasmic | 3.41 | 1.18 | 2.22 | 0.0102 | 0.417 | 0.124 | TRUE | FALSE | FALSE |
| **MAT2B** | Methionine adenosyltransferase 2 subunit beta | -0.648 | 0.374 | -1.02 | 0.572 | 0.8 | 0.386 | FALSE | FALSE | FALSE |
| **MATN4** | Matrilin 4 | 0.777 | 1.56 | -0.782 | 0.672 | 0.424 | 0.706 | FALSE | FALSE | FALSE |
| **MATR3** | Uncharacterized protein | -0.09 | 1.29 | -1.38 | 0.967 | 0.597 | 0.54 | FALSE | FALSE | FALSE |
| **MBNL1** | Muscleblind like splicing regulator 1 | -0.316 | 0.47 | -0.785 | 0.755 | 0.69 | 0.442 | FALSE | FALSE | FALSE |
| **MDH1** | Malate dehydrogenase | 0.466 | 0.211 | 0.255 | 0.675 | 0.884 | 0.849 | FALSE | FALSE | FALSE |
| **MDH2** | Malate dehydrogenase mitochondrial | -0.452 | 2.27 | -2.72 | 0.836 | 0.315 | 0.193 | FALSE | FALSE | FALSE |
| **MDK** | Midkine mitochondrial | -0.578 | -0.429 | -0.15 | 0.695 | 0.822 | 0.926 | FALSE | FALSE | FALSE |
| **MELTF** | Melanotransferrin | -0.757 | -1.04 | 0.287 | 0.774 | 0.739 | 0.921 | FALSE | FALSE | FALSE |

| | | | | | | | | | | |
|---|---|---|---|---|---|---|---|---|---|---|
| **MET** | Hepatocyte growth factor receptor | 0.158 | 0.335 | -0.177 | 0.89 | 0.787 | 0.876 | FALSE | FALSE | FALSE |
| **MFAP4** | Microfibril-associated glycoprotein 4 | 0.46 | -1.3 | 1.76 | 0.862 | 0.629 | 0.455 | FALSE | FALSE | FALSE |
| **MFGE8** | Lactadherin | 1.23 | -1.48 | 2.71 | 0.362 | 0.342 | 0.0726 | FALSE | FALSE | FALSE |
| **MGAT2** | Alpha-16-mannosyl-glycoprotein 2-beta-N-acetylglucosaminyltransferase | -0.684 | 0.212 | -0.895 | 0.546 | 0.891 | 0.444 | FALSE | FALSE | FALSE |
| **MIF** | Macrophage migration inhibitory factor | -0.895 | -1.78 | 0.882 | 0.434 | 0.236 | 0.475 | FALSE | FALSE | FALSE |
| **MINK1** | Misshapen like kinase 1 | -0.841 | -0.957 | 0.116 | 0.404 | 0.401 | 0.921 | FALSE | FALSE | FALSE |
| **MME** | Neprilysin | 2.9 | -1.29 | 4.19 | 0.178 | 0.629 | 0.0859 | FALSE | FALSE | FALSE |
| **MMP3** | Matrix metallopeptidase 3 | 1.17 | 1.1 | 0.0717 | 0.381 | 0.469 | 0.969 | FALSE | FALSE | FALSE |
| **MMP9** | Matrix metalloproteinase-9 | -5.22 | -1.63 | -3.59 | 0.00687 | 0.431 | 0.0808 | TRUE | FALSE | FALSE |
| **MOB1A** | MOB kinase activator 1A | -0.71 | -1.14 | 0.431 | 0.577 | 0.413 | 0.779 | FALSE | FALSE | FALSE |
| **MON2** | Protein MON2 homolog | -1.2 | -0.105 | -1.1 | 0.352 | 0.955 | 0.417 | FALSE | FALSE | FALSE |
| **MOV10** | RNA helicase | 0.3 | 1.09 | -0.794 | 0.892 | 0.642 | 0.73 | FALSE | FALSE | FALSE |
| **MPO** | MPO protein | -9.07 | -5.54 | -3.53 | 0.000627 | 0.0756 | 0.144 | TRUE | FALSE | FALSE |
| **MPP7** | MAGUK p55 subfamily member 7 | -1.16 | -0.373 | -0.79 | 0.427 | 0.858 | 0.642 | FALSE | FALSE | FALSE |

| | | | | | | | | | | |
|---|---|---|---|---|---|---|---|---|---|---|
| **MRE11** | Double-strand break repair protein | -1.2 | -0.53 | -0.675 | 0.389 | 0.765 | 0.68 | FALSE | FALSE | FALSE |
| **M-SAA3.2** | Serum amyloid A protein | 2.54 | 0.29 | 2.25 | 0.11 | 0.902 | 0.21 | FALSE | FALSE | FALSE |
| **MSH2** | DNA mismatch repair protein Msh2 | -1.3 | -0.663 | -0.638 | 0.363 | 0.7 | 0.706 | FALSE | FALSE | FALSE |
| **MSH6** | DNA mismatch repair protein | -1.57 | -1.13 | -0.448 | 0.178 | 0.4 | 0.757 | FALSE | FALSE | FALSE |
| **MSI2** | Musashi RNA binding protein 2 | -0.612 | -0.404 | -0.208 | 0.567 | 0.759 | 0.87 | FALSE | FALSE | FALSE |
| **MSLN** | Uncharacterized protein | 5.04 | -0.913 | 5.95 | 0.00755 | 0.681 | 0.008 | TRUE | FALSE | TRUE |
| **MSN** | Moesin | -1.62 | -2.37 | 0.759 | 0.562 | 0.434 | 0.827 | FALSE | FALSE | FALSE |
| **MSRA** | Peptide-methionine (S)-S-oxide reductase | 0.372 | 0.428 | -0.0559 | 0.709 | 0.716 | 0.966 | FALSE | FALSE | FALSE |
| **MSTN** | Growth/differentiation factor 8 | -1.12 | -1.1 | -0.0198 | 0.662 | 0.71 | 0.994 | FALSE | FALSE | FALSE |
| **MTA2** | Metastasis associated 1 family member 2 | -1.09 | -0.586 | -0.504 | 0.387 | 0.7 | 0.742 | FALSE | FALSE | FALSE |
| **MTAP** | S-methyl-5-thioadenosine phosphorylase | 0.158 | 1.23 | -1.07 | 0.895 | 0.335 | 0.351 | FALSE | FALSE | FALSE |
| **MTHFD1** | C-1-tetrahydrofolate synthase cytoplasmic | -0.225 | 0.0228 | -0.248 | 0.842 | 0.988 | 0.845 | FALSE | FALSE | FALSE |
| **MTPN** | Myotrophin | -3.46 | -3.32 | -0.139 | 0.0609 | 0.214 | 0.954 | FALSE | FALSE | FALSE |

| | | | | | | | | | | |
|---|---|---|---|---|---|---|---|---|---|---|
| **MTREX** | Mtr4 exosome RNA helicase | -1.12 | 0.469 | -1.58 | 0.411 | 0.793 | 0.277 | FALSE | FALSE | FALSE |
| **MUC1** | Mucin-1 | 1.85 | 0.0681 | 1.78 | 0.0873 | 0.966 | 0.146 | FALSE | FALSE | FALSE |
| **MUC13** | Mucin 13, cell surface associated | -1.75 | -0.851 | -0.902 | 0.264 | 0.66 | 0.62 | FALSE | FALSE | FALSE |
| **MUC19** | Mucin-19 | -0.997 | 3.01 | -4.01 | 0.702 | 0.312 | 0.146 | FALSE | FALSE | FALSE |
| **MUC4** | Mucin 4  cell surface associated | 7.98 | 0.687 | 7.3 | 0.000411 | 0.763 | 0.0021 | TRUE | FALSE | TRUE |
| **MVB12A** | Multivesicular body subunit 12A | -0.607 | 0.344 | -0.951 | 0.654 | 0.849 | 0.489 | FALSE | FALSE | FALSE |
| **MVP** | Major vault protein | 4.27 | 2.01 | 2.26 | 0.0568 | 0.435 | 0.351 | FALSE | FALSE | FALSE |
| **MX1** | Interferon-induced GTP-binding protein Mx1 | -1.47 | -0.102 | -1.36 | 0.452 | 0.967 | 0.529 | FALSE | FALSE | FALSE |
| **MYADM** | Myeloid-associated differentiation marker | 0.895 | 0.128 | 0.767 | 0.384 | 0.936 | 0.489 | FALSE | FALSE | FALSE |
| **MYBBP1A** | MYB binding protein 1a | 0.381 | 0.555 | -0.175 | 0.709 | 0.642 | 0.88 | FALSE | FALSE | FALSE |
| **MYCBP2** | RCR-type E3 ubiquitin transferase | -1.22 | 0.388 | -1.61 | 0.356 | 0.829 | 0.253 | FALSE | FALSE | FALSE |
| **MYH10** | Myosin-10 | 2.94 | 2.94 | -0.00437 | 0.0107 | 0.0719 | 0.997 | TRUE | FALSE | FALSE |
| **MYH11** | Myosin heavy chain 11 | -0.097 | 0.643 | -0.74 | 0.923 | 0.599 | 0.503 | FALSE | FALSE | FALSE |
| **MYH14** | Myosin heavy chain 14 | 4.95 | 1.11 | 3.84 | 0.00375 | 0.541 | 0.0314 | TRUE | FALSE | TRUE |
| **MYH9** | Myosin heavy chain 9 | 2.23 | 0.401 | 1.83 | 0.114 | 0.842 | 0.239 | FALSE | FALSE | FALSE |
| **MYL12B** | Myosin regulatory light chain 12B | 2.35 | 0.608 | 1.74 | 0.0812 | 0.721 | 0.241 | FALSE | FALSE | FALSE |

| | | | | | | | | | | |
|---|---|---|---|---|---|---|---|---|---|---|
| **MYL3** | Myosin light chain 3 | 0.914 | 0.632 | 0.281 | 0.352 | 0.587 | 0.824 | FALSE | FALSE | FALSE |
| **MYL6** | Myosin light polypeptide 6 | 1.09 | 0.554 | 0.536 | 0.402 | 0.734 | 0.73 | FALSE | FALSE | FALSE |
| **MYO1A** | Unconventional myosin-Ia | 1.46 | -1.55 | 3.01 | 0.324 | 0.363 | 0.0726 | FALSE | FALSE | FALSE |
| **MYO1B** | Myosin IB | 4.53 | -0.63 | 5.16 | 0.00898 | 0.768 | 0.0127 | TRUE | FALSE | TRUE |
| **MYO1C** | Unconventional myosin-Ic | 1.91 | -0.379 | 2.29 | 0.133 | 0.833 | 0.11 | FALSE | FALSE | FALSE |
| **MYO1D** | Unconventional myosin-Id | 0.278 | -1.27 | 1.54 | 0.845 | 0.363 | 0.239 | FALSE | FALSE | FALSE |
| **MYO1E** | Myosin IE | -0.94 | -1.19 | 0.247 | 0.362 | 0.326 | 0.85 | FALSE | FALSE | FALSE |
| **MYO1F** | Myosin IF | -0.975 | -2.04 | 1.07 | 0.376 | 0.197 | 0.356 | FALSE | FALSE | FALSE |
| **MYO5B** | Myosin VB | -0.889 | -0.665 | -0.223 | 0.546 | 0.7 | 0.898 | FALSE | FALSE | FALSE |
| **MYO6** | Unconventional myosin-VI | 0.415 | 1.38 | -0.96 | 0.85 | 0.524 | 0.657 | FALSE | FALSE | FALSE |
| **MYO9B** | Myosin IXB | -0.0561 | -0.103 | 0.0468 | 0.973 | 0.966 | 0.985 | FALSE | FALSE | FALSE |
| **MYOF** | Myoferlin | 5.17 | 0.155 | 5.02 | 0.00839 | 0.955 | 0.0242 | TRUE | FALSE | TRUE |
| **NAA15** | N-alpha-acetyltransferase 15 | -0.928 | 0.873 | -1.8 | 0.595 | 0.666 | 0.315 | FALSE | FALSE | FALSE |
| **NANS** | N-acetylneuraminate synthase NatA auxiliary subunit | -0.643 | -1.28 | 0.636 | 0.609 | 0.356 | 0.647 | FALSE | FALSE | FALSE |
| **NAP1L4** | Nucleosome assembly protein 1-like 4 | 0.536 | -0.669 | 1.2 | 0.684 | 0.662 | 0.363 | FALSE | FALSE | FALSE |
| **NARS1** | Asparagine--tRNA ligase | 1.08 | -0.0295 | 1.11 | 0.452 | 0.99 | 0.474 | FALSE | FALSE | FALSE |
| **NAT10** | RNA cytidine acetyltransferase | -2.27 | -0.119 | -2.15 | 0.152 | 0.959 | 0.226 | FALSE | FALSE | FALSE |

| | | | | | | | | | | |
|---|---|---|---|---|---|---|---|---|---|---|
| **NCF1** | Neutrophil cytosol factor 1 | 0.98 | 1.14 | -0.156 | 0.484 | 0.472 | 0.921 | FALSE | FALSE | FALSE |
| **NCF2** | Neutrophil cytosol factor 2 | 1.43 | 0.979 | 0.455 | 0.174 | 0.417 | 0.723 | FALSE | FALSE | FALSE |
| **NCKAP1** | NCK associated protein 1 | -0.304 | 0.304 | -0.608 | 0.765 | 0.816 | 0.575 | FALSE | FALSE | FALSE |
| **NCKAP1L** | NCK associated protein 1 like | 0.536 | 2.6 | -2.06 | 0.707 | 0.186 | 0.174 | FALSE | FALSE | FALSE |
| **NCL** | Nucleolin | 9.27 | 4.26 | 5.01 | 0.00295 | 0.244 | 0.107 | TRUE | FALSE | FALSE |
| **NCSTN** | Nicastrin | 0.912 | -1.1 | 2.01 | 0.411 | 0.388 | 0.101 | FALSE | FALSE | FALSE |
| **NDRG2** | Protein NDRG2 | 0.158 | 1.22 | -1.07 | 0.895 | 0.337 | 0.355 | FALSE | FALSE | FALSE |
| **NEMF** | Nuclear export mediator factor | -1.63 | -1.87 | 0.247 | 0.184 | 0.25 | 0.873 | FALSE | FALSE | FALSE |
| **NEU1** | Sialidase-1 | 0.158 | 0.775 | -0.617 | 0.892 | 0.484 | 0.567 | FALSE | FALSE | FALSE |
| **NF2** | Neurofibromin 2 | -0.904 | 2.57 | -3.47 | 0.673 | 0.285 | 0.123 | FALSE | FALSE | FALSE |
| **NIBAN1** | Niban apoptosis regulator 1 | -3.17 | -3.42 | 0.252 | 0.086 | 0.201 | 0.912 | FALSE | FALSE | FALSE |
| **NIBAN2** | Niban apoptosis regulator 2 | 3.11 | -0.552 | 3.67 | 0.012 | 0.716 | 0.014 | TRUE | FALSE | TRUE |
| **NID1** | NID1 protein | -2.19 | -1.86 | -0.328 | 0.167 | 0.326 | 0.865 | FALSE | FALSE | FALSE |
| **NME2** | Nucleoside diphosphate kinase B | -2.44 | -1.45 | -0.995 | 0.0275 | 0.28 | 0.403 | TRUE | FALSE | FALSE |
| **NMRAL1** | NmrA-like family domain-containing protein 1 | -0.586 | -0.41 | -0.176 | 0.56 | 0.737 | 0.879 | FALSE | FALSE | FALSE |
| **NOP2** | NOP2 nucleolar protein | -2.87 | -1.98 | -0.883 | 0.0383 | 0.257 | 0.571 | TRUE | FALSE | FALSE |
| **NOP58** | NOP58 ribonucleoprotein | -0.905 | -0.189 | -0.715 | 0.4 | 0.903 | 0.555 | FALSE | FALSE | FALSE |

| | | | | | | | | | | |
|---|---|---|---|---|---|---|---|---|---|---|
| **NPC1** | NPC intracellular cholesterol transporter 1 | 0.312 | 0.067 | 0.245 | 0.871 | 0.975 | 0.898 | FALSE | FALSE | FALSE |
| **NPEPPS** | Aminopeptidase | -1.75 | -1.12 | -0.639 | 0.229 | 0.512 | 0.716 | FALSE | FALSE | FALSE |
| **NPM1** | Nucleophosmin | 3.8 | 2.2 | 1.61 | 0.0178 | 0.265 | 0.345 | TRUE | FALSE | FALSE |
| **NSDHL** | Sterol-4-alpha-carboxylate 3-dehydrogenase decarboxylating | -1.33 | -0.264 | -1.06 | 0.381 | 0.903 | 0.524 | FALSE | FALSE | FALSE |
| **NSF** | Vesicle-fusing ATPase | -0.593 | 0.804 | -1.4 | 0.684 | 0.633 | 0.342 | FALSE | FALSE | FALSE |
| **NT5C2** | Cytosolic purine 5-nucleotidase | -1.26 | 0.00379 | -1.27 | 0.375 | 0.998 | 0.395 | FALSE | FALSE | FALSE |
| **NT5E** | 5-nucleotidase | 9.17 | 2.06 | 7.11 | 0.000135 | 0.319 | 0.00207 | TRUE | FALSE | TRUE |
| **NT5E.1** | 5-nucleotidase | 2.43 | 2.88 | -0.457 | 0.0548 | 0.129 | 0.775 | FALSE | FALSE | FALSE |
| **NUCB1** | Nucleobindin-1 | 0.454 | -0.704 | 1.16 | 0.667 | 0.549 | 0.277 | FALSE | FALSE | FALSE |
| **NUDC** | Nuclear migration protein nudC | -0.523 | 0.142 | -0.665 | 0.655 | 0.934 | 0.592 | FALSE | FALSE | FALSE |
| **NUDT3** | Diphosphoinositol polyphosphate phosphohydrolase 1 | 1.47 | 0.452 | 1.01 | 0.246 | 0.784 | 0.46 | FALSE | FALSE | FALSE |
| **NUMA1** | Nuclear mitotic apparatus protein 1 | 2.67 | 1.63 | 1.04 | 0.247 | 0.553 | 0.71 | FALSE | FALSE | FALSE |
| **NUTF2** | Nuclear transport factor 2 | -0.629 | 0.798 | -1.43 | 0.662 | 0.629 | 0.327 | FALSE | FALSE | FALSE |
| **OLFM4** | Olfactomedin 4 | -12.1 | -7.38 | -4.67 | 4.91E-05 | 0.00373 | 0.0227 | TRUE | TRUE | TRUE |
| **OLFML2B** | Olfactomedin-like protein 2B | -1.06 | -0.112 | -0.945 | 0.628 | 0.967 | 0.703 | FALSE | FALSE | FALSE |
| **OLFML3** | Olfactomedin-like protein 3 | -1.35 | -0.539 | -0.812 | 0.402 | 0.801 | 0.663 | FALSE | FALSE | FALSE |

| | | | | | | | | | | |
|---|---|---|---|---|---|---|---|---|---|---|
| **OSBP** | Oxysterol-binding protein | -1.87 | -1.42 | -0.448 | 0.228 | 0.417 | 0.824 | FALSE | FALSE | FALSE |
| **OSBPL10** | Oxysterol-binding protein | -1.38 | 0.617 | -1.99 | 0.373 | 0.749 | 0.229 | FALSE | FALSE | FALSE |
| **OSTF1** | Osteoclast-stimulating factor 1 | -0.876 | -1.45 | 0.574 | 0.583 | 0.403 | 0.759 | FALSE | FALSE | FALSE |
| **OTUB1** | Ubiquitin thioesterase | 1.07 | 0.278 | 0.796 | 0.345 | 0.859 | 0.521 | FALSE | FALSE | FALSE |
| **OXSR1** | Non-specific serine/threonine protein kinase | -0.666 | -0.723 | 0.0571 | 0.487 | 0.512 | 0.965 | FALSE | FALSE | FALSE |
| **P04815** | Spleen trypsin inhibitor I | -0.606 | 1.26 | -1.87 | 0.684 | 0.431 | 0.229 | FALSE | FALSE | FALSE |
| **P13752** | BOLA class I histocompatibility antigen alpha chain BL3-6 | 0.396 | -0.573 | 0.968 | 0.74 | 0.676 | 0.416 | FALSE | FALSE | FALSE |
| **P13753** | BOLA class I histocompatibility antigen alpha chain BL3-7 | 1.88 | -0.57 | 2.45 | 0.044 | 0.62 | 0.0254 | TRUE | FALSE | TRUE |
| **P4HB** | Protein disulfide-isomerase | 1.8 | 1.21 | 0.599 | 0.084 | 0.335 | 0.62 | FALSE | FALSE | FALSE |
| **P54281** | Calcium-activated chloride channel regulator 1 | -2.89 | -2.01 | -0.883 | 0.136 | 0.373 | 0.706 | FALSE | FALSE | FALSE |
| **p97bcnt2** | Bucentaur-2 | 4.33 | 1.66 | 2.67 | 0.00203 | 0.268 | 0.0601 | TRUE | FALSE | FALSE |
| **PA2G4** | Proliferation-associated 2G4 | -1.48 | 0.443 | -1.93 | 0.243 | 0.789 | 0.174 | FALSE | FALSE | FALSE |
| **PABPC1** | Polyadenylate-binding protein 1 | 4.62 | 2.1 | 2.52 | 0.00597 | 0.271 | 0.148 | TRUE | FALSE | FALSE |

| | | | | | | | | | | |
|---|---|---|---|---|---|---|---|---|---|---|
| **PABPC4** | Polyadenylate-binding protein | 2.04 | 2.09 | -0.0529 | 0.0704 | 0.201 | 0.975 | FALSE | FALSE | FALSE |
| **PABPN1** | PABPN1 protein | 1.64 | 1.8 | -0.155 | 0.376 | 0.393 | 0.943 | FALSE | FALSE | FALSE |
| **PACSIN2** | Protein kinase C and casein kinase substrate in neurons 2 | 0.897 | 0.449 | 0.448 | 0.356 | 0.7 | 0.692 | FALSE | FALSE | FALSE |
| **PADI4** | Protein-arginine deiminase | -8.09 | -3.7 | -4.38 | 0.00546 | 0.265 | 0.146 | TRUE | FALSE | FALSE |
| **PAFAH1B1** | Platelet-activating factor acetylhydrolase IB subunit alpha | 2.69 | 1.41 | 1.28 | 0.0119 | 0.271 | 0.261 | TRUE | FALSE | FALSE |
| **PAFAH1B2P68402** | Platelet-activating factor acetylhydrolase IB subunit alpha2 | -0.442 | -0.933 | 0.492 | 0.739 | 0.518 | 0.744 | FALSE | FALSE | FALSE |
| **PAFAH1B3** | Platelet-activating factor acetylhydrolase IB subunit alpha1 | -0.474 | -0.142 | -0.332 | 0.756 | 0.951 | 0.856 | FALSE | FALSE | FALSE |
| **PAICS** | Phosphoribosylaminoimidazole carboxylase phosphoribosylaminoimidazole succinocarboxamide synthetase | -0.934 | -0.51 | -0.424 | 0.377 | 0.69 | 0.74 | FALSE | FALSE | FALSE |
| **PAK1** | Serine/threonine-protein kinase PAK 1 | 0.158 | 1.96 | -1.8 | 0.905 | 0.24 | 0.202 | FALSE | FALSE | FALSE |

| | | | | | | | | | | |
|---|---|---|---|---|---|---|---|---|---|---|
| **PAK2** | Non-specific serine/threonine protein kinase | -0.427 | -0.268 | -0.159 | 0.654 | 0.828 | 0.882 | FALSE | FALSE | FALSE |
| **PAK4** | Non-specific serine/threonine protein kinase | -1.43 | -0.814 | -0.62 | 0.309 | 0.639 | 0.716 | FALSE | FALSE | FALSE |
| **PALS1** | Protein PALS1 | -1.2 | -0.501 | -0.702 | 0.204 | 0.666 | 0.497 | FALSE | FALSE | FALSE |
| **PARD6B** | Par-6 family cell polarity regulator beta | -0.789 | -0.0512 | -0.738 | 0.413 | 0.967 | 0.475 | FALSE | FALSE | FALSE |
| **PARK7** | Parkinson disease protein 7 homolog | 0.674 | -0.0201 | 0.694 | 0.602 | 0.992 | 0.62 | FALSE | FALSE | FALSE |
| **PARP1** | Poly [ADP-ribose] polymerase | 0.345 | 2.95 | -2.6 | 0.87 | 0.224 | 0.177 | FALSE | FALSE | FALSE |
| **PARP4** | Poly [ADP-ribose] polymerase | -1.16 | 0.795 | -1.96 | 0.428 | 0.66 | 0.228 | FALSE | FALSE | FALSE |
| **PATJ** | PATJ crumbs cell polarity complex component | -0.674 | -0.741 | 0.0668 | 0.743 | 0.763 | 0.979 | FALSE | FALSE | FALSE |
| **PBDC1** | Polysaccharide biosynthesis domain containing 1 | 0.158 | 1.43 | -1.27 | 0.898 | 0.285 | 0.29 | FALSE | FALSE | FALSE |
| **PCBP1** | Poly(rC)-binding protein 1 | 1.98 | 0.124 | 1.86 | 0.246 | 0.959 | 0.315 | FALSE | FALSE | FALSE |
| **PCBP2** | Poly(RC) binding protein 2 | 1.63 | 0.414 | 1.21 | 0.139 | 0.768 | 0.312 | FALSE | FALSE | FALSE |
| **PCMT1** | Protein-L-isoaspartate O-methyltransferase | -0.451 | 0.232 | -0.683 | 0.689 | 0.876 | 0.571 | FALSE | FALSE | FALSE |

| | | | | | | | | | | |
|---|---|---|---|---|---|---|---|---|---|---|
| **PCYOX1** | Prenylcysteine oxidase 1 | -0.323 | 0.325 | -0.648 | 0.755 | 0.801 | 0.554 | FALSE | FALSE | FALSE |
| **PDCD10** | Programmed cell death 10 | -1.25 | -1.17 | -0.0808 | 0.354 | 0.435 | 0.965 | FALSE | FALSE | FALSE |
| **PDCD6** | PDCD6 protein | -1.52 | 0.125 | -1.64 | 0.11 | 0.934 | 0.129 | FALSE | FALSE | FALSE |
| **PDCD6IP** | Programmed cell death 6 interacting protein | 1.05 | 0.215 | 0.831 | 0.348 | 0.89 | 0.482 | FALSE | FALSE | FALSE |
| **PDE4DIP** | Phosphodiesterase 4D interacting protein | -1.19 | 0.064 | -1.26 | 0.294 | 0.967 | 0.304 | FALSE | FALSE | FALSE |
| **PDGFC** | Platelet-derived growth factor C | -1.3 | 0.273 | -1.57 | 0.264 | 0.867 | 0.227 | FALSE | FALSE | FALSE |
| **PDIA3** | Protein disulfide-isomerase | 3.1 | 1.23 | 1.88 | 0.0248 | 0.434 | 0.223 | TRUE | FALSE | FALSE |
| **PDIA4** | Protein disulfide-isomerase A4 | -0.915 | -0.872 | -0.0429 | 0.434 | 0.519 | 0.977 | FALSE | FALSE | FALSE |
| **PDIA6** | Protein disulfide-isomerase A6 | -0.174 | 0.401 | -0.575 | 0.895 | 0.801 | 0.68 | FALSE | FALSE | FALSE |
| **PDLIM1** | PDZ and LIM domain 1 | -0.197 | -0.344 | 0.146 | 0.869 | 0.788 | 0.901 | FALSE | FALSE | FALSE |
| **PDLIM5** | PDZ and LIM domain 5 | 0.527 | -0.426 | 0.954 | 0.697 | 0.804 | 0.497 | FALSE | FALSE | FALSE |
| **PDS5A** | PDS5 cohesin associated factor A | 0.519 | 1.51 | -0.993 | 0.679 | 0.285 | 0.433 | FALSE | FALSE | FALSE |
| **PDXK** | Pyridoxal kinase | 4.15 | 1.2 | 2.95 | 0.00278 | 0.393 | 0.037 | TRUE | FALSE | TRUE |
| **PDZK1** | Na(+)/H(+) exchange regulatory cofactor NHE-RF3 | 1.51 | 0.83 | 0.685 | 0.243 | 0.598 | 0.653 | FALSE | FALSE | FALSE |

| | | | | | | | | | | |
|---|---|---|---|---|---|---|---|---|---|---|
| **PEBP1** | Phosphatidylethanolamine-binding protein 1 | 2.68 | 0.0491 | 2.63 | 0.0114 | 0.971 | 0.0294 | TRUE | FALSE | TRUE |
| **PEF1** | Penta-EF-hand domain containing 1 | -0.334 | 0.254 | -0.588 | 0.743 | 0.852 | 0.586 | FALSE | FALSE | FALSE |
| **PFKFB2** | 6-phosphofructo-2-kinase/fructose-26-bisphosphatase 2 | -0.236 | -0.483 | 0.247 | 0.852 | 0.716 | 0.856 | FALSE | FALSE | FALSE |
| **PFKL** | ATP-dependent 6-phosphofructokinase liver type | 0.427 | -0.241 | 0.668 | 0.702 | 0.87 | 0.575 | FALSE | FALSE | FALSE |
| **PFKP** | ATP-dependent 6-phosphofructokinase | -0.587 | -0.533 | -0.0546 | 0.625 | 0.7 | 0.975 | FALSE | FALSE | FALSE |
| **PFN2** | Profilin-2 | -0.739 | -1.37 | 0.629 | 0.439 | 0.257 | 0.559 | FALSE | FALSE | FALSE |
| **PGAM1** | Phosphoglycerate mutase 1 | 0.116 | -0.267 | 0.382 | 0.935 | 0.896 | 0.839 | FALSE | FALSE | FALSE |
| **PGAP6** | Post-glycosylphosphatidylinositol attachment to proteins 6 | -0.981 | 0.559 | -1.54 | 0.681 | 0.859 | 0.544 | FALSE | FALSE | FALSE |
| **PGD** | 6-phosphogluconate dehydrogenase decarboxylating | -4.42 | -4.48 | 0.0562 | 0.0275 | 0.129 | 0.985 | TRUE | FALSE | FALSE |
| **PGK1** | Phosphoglycerate kinase 1 | -1.21 | -2.39 | 1.19 | 0.357 | 0.197 | 0.387 | FALSE | FALSE | FALSE |

| | | | | | | | | | | |
|---|---|---|---|---|---|---|---|---|---|---|
| **PGLS** | 6-phosphogluconolactonase | 0.116 | -2.17 | 2.29 | 0.924 | 0.214 | 0.0919 | FALSE | FALSE | FALSE |
| **PGLYRP1** | Peptidoglycan recognition protein 1 | -9.84 | -7.57 | -2.27 | 9.68E-05 | 0.00373 | 0.237 | TRUE | TRUE | FALSE |
| **PGM1** | Phosphoglucomutase-1 | -0.915 | -0.245 | -0.67 | 0.388 | 0.867 | 0.571 | FALSE | FALSE | FALSE |
| **PGM2** | Phosphoglucomutase 2 | 1.07 | -0.883 | 1.96 | 0.249 | 0.407 | 0.0678 | FALSE | FALSE | FALSE |
| **PGR** | Progesterone receptor | -0.542 | 2.04 | -2.58 | 0.759 | 0.31 | 0.168 | FALSE | FALSE | FALSE |
| **PGRMC1** | Membrane-associated progesterone receptor component 1 | 0.672 | 0.993 | -0.321 | 0.607 | 0.484 | 0.841 | FALSE | FALSE | FALSE |
| **PHGDH** | D-3-phosphoglycerate dehydrogenase | 2.82 | 0.42 | 2.4 | 0.074 | 0.852 | 0.179 | FALSE | FALSE | FALSE |
| **PHPT1** | 14 kDa phosphohistidine phosphatase | -0.197 | -0.16 | -0.0372 | 0.886 | 0.927 | 0.98 | FALSE | FALSE | FALSE |
| **PI3** | Peptidase inhibitor 3 skin-derived (SKALP) | -2.83 | -2.14 | -0.693 | 0.132 | 0.339 | 0.769 | FALSE | FALSE | FALSE |
| **PI4KA** | 1-phosphatidylinositol 4-kinase | -0.256 | 0.433 | -0.689 | 0.823 | 0.733 | 0.544 | FALSE | FALSE | FALSE |

| | | | | | | | | | | |
|---|---|---|---|---|---|---|---|---|---|---|
| **PICALM** | Phosphatidylinositol binding clathrin assembly protein | 0.158 | 2.04 | -1.88 | 0.905 | 0.236 | 0.185 | FALSE | FALSE | FALSE |
| **PIGR** | Polymeric immunoglobulin receptor | 1.39 | -0.208 | 1.6 | 0.174 | 0.884 | 0.166 | FALSE | FALSE | FALSE |
| **PIP** | Prolactin-inducible protein homolog | -0.442 | 1.09 | -1.54 | 0.688 | 0.363 | 0.185 | FALSE | FALSE | FALSE |
| **PITPNB** | Phosphatidylinositol transfer protein beta isoform | -0.889 | -0.0149 | -0.874 | 0.413 | 0.993 | 0.455 | FALSE | FALSE | FALSE |
| **PKM** | Pyruvate kinase | -2.68 | -2.06 | -0.62 | 0.0499 | 0.253 | 0.708 | TRUE | FALSE | FALSE |
| **PKN1** | Serine/threonine-protein kinase N1 | -2.11 | -1.62 | -0.498 | 0.172 | 0.365 | 0.803 | FALSE | FALSE | FALSE |
| **PKP1** | Plakophilin-1 | -1.58 | -1.82 | 0.247 | 0.338 | 0.339 | 0.901 | FALSE | FALSE | FALSE |
| **PLA2G4A** | Phospholipase A2 | 0.158 | 1.76 | -1.61 | 0.903 | 0.256 | 0.229 | FALSE | FALSE | FALSE |
| **PLA2G7** | Platelet-activating factor acetylhydrolase | -1.3 | -1.82 | 0.522 | 0.474 | 0.381 | 0.824 | FALSE | FALSE | FALSE |
| **PLAC8A** | Placenta specific 8 A | -1.2 | 0.734 | -1.93 | 0.666 | 0.842 | 0.497 | FALSE | FALSE | FALSE |
| **PLAT** | Tissue-type plasminogen activator | -0.0652 | -0.175 | 0.11 | 0.978 | 0.959 | 0.975 | FALSE | FALSE | FALSE |
| **PLAUR** | Urokinase plasminogen activator surface receptor | -1.18 | -1.65 | 0.472 | 0.29 | 0.253 | 0.725 | FALSE | FALSE | FALSE |
| **PLCB4** | 1-phosphatidylinositol 45-bisphosphate phosphodiesterase | -1.72 | -0.125 | -1.59 | 0.246 | 0.955 | 0.322 | FALSE | FALSE | FALSE |

| | | | | | | | | | | |
|---|---|---|---|---|---|---|---|---|---|---|
| **PLCD1** | 1-phosphatidylinositol 45-bisphosphate phosphodiesterase delta-1 | -0.948 | 1.77 | -2.72 | 0.514 | 0.304 | 0.0881 | FALSE | FALSE | FALSE |
| **PLCG2** | 1-phosphatidylinositol 45-bisphosphate phosphodiesterase gamma | 1.53 | 2.52 | -0.994 | 0.531 | 0.363 | 0.73 | FALSE | FALSE | FALSE |
| **PLEC** | Plectin | 2.3 | 0.407 | 1.9 | 0.29 | 0.896 | 0.415 | FALSE | FALSE | FALSE |
| **PLEK** | Pleckstrin | -0.531 | -0.172 | -0.359 | 0.685 | 0.931 | 0.824 | FALSE | FALSE | FALSE |
| **PLEKHB2** | Pleckstrin homology domain containing B2 | -0.154 | 0.555 | -0.709 | 0.9 | 0.69 | 0.583 | FALSE | FALSE | FALSE |
| **PLET1** | Placenta-expressed transcript 1 protein | -3.56 | -0.631 | -2.92 | 0.104 | 0.839 | 0.229 | FALSE | FALSE | FALSE |
| **PLG** | Plasminogen | -2.23 | -1.88 | -0.343 | 0.436 | 0.585 | 0.919 | FALSE | FALSE | FALSE |
| **PLOD1** | Procollagen-lysine 5-dioxygenase | -0.998 | -1.96 | 0.967 | 0.61 | 0.363 | 0.655 | FALSE | FALSE | FALSE |
| **PLS1** | Plastin-1 | -1.03 | -1.07 | 0.0395 | 0.613 | 0.651 | 0.986 | FALSE | FALSE | FALSE |
| **PLS3** | Plastin-3 | -1.71 | -1.7 | -0.00313 | 0.0904 | 0.232 | 0.997 | FALSE | FALSE | FALSE |
| **PLSCR2** | Phospholipid scramblase 2 | 0.927 | -1.2 | 2.13 | 0.486 | 0.417 | 0.145 | FALSE | FALSE | FALSE |
| **PLXNB2** | Plexin B2 | -0.153 | 0.48 | -0.633 | 0.903 | 0.749 | 0.647 | FALSE | FALSE | FALSE |
| **PNP** | Purine nucleoside phosphorylase | 0.0425 | -1.61 | 1.65 | 0.97 | 0.257 | 0.185 | FALSE | FALSE | FALSE |
| **PODXL** | Podocalyxin | 0.333 | 0.213 | 0.12 | 0.75 | 0.876 | 0.918 | FALSE | FALSE | FALSE |
| **POLR2A** | DNA-directed RNA polymerase subunit | -1.35 | -1.6 | 0.247 | 0.231 | 0.259 | 0.857 | FALSE | FALSE | FALSE |

| | | | | | | | | | | |
|---|---|---|---|---|---|---|---|---|---|---|
| **POLR3B** | DNA-directed RNA polymerase subunit beta | -1.33 | 0.0289 | -1.36 | 0.434 | 0.992 | 0.463 | FALSE | FALSE | FALSE |
| **PON1** | Paraoxonase | -1.88 | -1.72 | -0.165 | 0.249 | 0.364 | 0.93 | FALSE | FALSE | FALSE |
| **PPA1** | Inorganic pyrophosphatase | 0.158 | 0.673 | -0.515 | 0.892 | 0.566 | 0.654 | FALSE | FALSE | FALSE |
| **PPBP** | C-X-C motif chemokine | 0.661 | -0.0885 | 0.75 | 0.549 | 0.955 | 0.524 | FALSE | FALSE | FALSE |
| **PPIA** | Peptidyl-prolyl cis-trans isomerase A | -0.136 | -1.16 | 1.02 | 0.907 | 0.388 | 0.409 | FALSE | FALSE | FALSE |
| **PPIB** | Peptidyl-prolyl cis-trans isomerase B | 0.353 | -0.25 | 0.603 | 0.75 | 0.863 | 0.613 | FALSE | FALSE | FALSE |
| **PPIH** | Peptidyl-prolyl cis-trans isomerase H | -1.01 | 0.669 | -1.68 | 0.551 | 0.749 | 0.336 | FALSE | FALSE | FALSE |
| **PPL** | Periplakin | 0.428 | 0.875 | -0.447 | 0.832 | 0.685 | 0.845 | FALSE | FALSE | FALSE |
| **PPM1F** | Protein phosphatase Mg2+/Mn2+ dependent 1F | -0.833 | 0.312 | -1.15 | 0.581 | 0.876 | 0.469 | FALSE | FALSE | FALSE |
| **PPP1CA** | Serine/threonine-protein phosphatase PP1-alpha catalytic subunit | -0.203 | -1.18 | 0.979 | 0.902 | 0.512 | 0.571 | FALSE | FALSE | FALSE |
| **PPP1CB** | Serine/threonine-protein phosphatase PP1-beta catalytic subunit | 0.979 | 0.978 | 0.000886 | 0.394 | 0.453 | 1 | FALSE | FALSE | FALSE |

| | | | | | | | | | | |
|---|---|---|---|---|---|---|---|---|---|---|
| **PPP1R21** | Protein phosphatase 1 regulatory subunit 21 | -1.54 | 0.631 | -2.17 | 0.354 | 0.763 | 0.229 | FALSE | FALSE | FALSE |
| **PPP1R7** | Protein phosphatase 1 regulatory subunit 7 | 0.886 | 1.37 | -0.487 | 0.529 | 0.388 | 0.777 | FALSE | FALSE | FALSE |
| **PPP2CA** | Serine/threonine-protein phosphatase 2A catalytic subunit alpha isoform | -0.244 | 0.0589 | -0.303 | 0.84 | 0.967 | 0.824 | FALSE | FALSE | FALSE |
| **PPP2R1A** | Serine/threonine-protein phosphatase 2A 65 kDa regulatory subunit A alpha isoform | 0.387 | -1.01 | 1.39 | 0.809 | 0.561 | 0.363 | FALSE | FALSE | FALSE |
| **PPP2R2A** | Serine/threonine-protein phosphatase 2A 55 kDa regulatory subunit B | -1.02 | -0.594 | -0.43 | 0.304 | 0.629 | 0.72 | FALSE | FALSE | FALSE |
| **PPP3CA** | Serine/threonine-protein phosphatase | -0.161 | -0.0966 | -0.0641 | 0.892 | 0.952 | 0.964 | FALSE | FALSE | FALSE |
| **PRDX1** | Peroxiredoxin-1 | 4.63 | 0.1 | 4.53 | 0.000892 | 0.955 | 0.00303 | TRUE | FALSE | TRUE |
| **PRDX2** | Peroxiredoxin-2 | 2.79 | -0.281 | 3.07 | 0.0787 | 0.903 | 0.0873 | FALSE | FALSE | FALSE |
| **PRDX5** | Peroxiredoxin-5 mitochondrial | -0.849 | -1.56 | 0.706 | 0.619 | 0.4 | 0.72 | FALSE | FALSE | FALSE |

| | | | | | | | | | | |
|---|---|---|---|---|---|---|---|---|---|---|
| **PRDX6** | Peroxiredoxin-6 | 0.059 | -0.761 | 0.82 | 0.966 | 0.631 | 0.571 | FALSE | FALSE | FALSE |
| **PRKACB** | cAMP-dependent protein kinase | -0.402 | -0.769 | 0.368 | 0.74 | 0.572 | 0.802 | FALSE | FALSE | FALSE |
| **PRKAR2A** | cAMP-dependent protein kinase type II-alpha regulatory subunit | 4.75 | 0.902 | 3.85 | 0.00375 | 0.618 | 0.0254 | TRUE | FALSE | TRUE |
| **PRKAR2B** | cAMP-dependent protein kinase type II-beta regulatory subunit | 0.942 | 0.365 | 0.577 | 0.452 | 0.835 | 0.701 | FALSE | FALSE | FALSE |
| **PRKCA** | Protein kinase C alpha type | -1.26 | 0.109 | -1.37 | 0.362 | 0.956 | 0.345 | FALSE | FALSE | FALSE |
| **PRKCD** | Protein kinase C delta type | 0.0107 | -0.624 | 0.635 | 0.992 | 0.597 | 0.561 | FALSE | FALSE | FALSE |
| **PRKCI** | Protein kinase C | -1.02 | -0.027 | -0.995 | 0.387 | 0.988 | 0.428 | FALSE | FALSE | FALSE |
| **PRKD2** | Serine/threonine-protein kinase | -0.759 | 1.06 | -1.82 | 0.642 | 0.565 | 0.277 | FALSE | FALSE | FALSE |
| **PRMT1** | Protein arginine methyltransferase 1 | -1.08 | 0.209 | -1.29 | 0.4 | 0.915 | 0.344 | FALSE | FALSE | FALSE |
| **PRMT5** | Protein arginine N-methyltransferase 5 | -1.2 | 0.339 | -1.53 | 0.382 | 0.859 | 0.294 | FALSE | FALSE | FALSE |
| **PRNP** | Major prion protein | 1.18 | 0.304 | 0.88 | 0.243 | 0.829 | 0.416 | FALSE | FALSE | FALSE |
| **PROM1** | Prominin 1 | 4.69 | 0.405 | 4.28 | 0.0116 | 0.873 | 0.0397 | TRUE | FALSE | TRUE |
| **PROM2** | Prominin 2 | 6.56 | -0.971 | 7.53 | 0.0152 | 0.781 | 0.019 | TRUE | FALSE | TRUE |
| **PRPF19** | Pre-mRNA-processing factor 19 | 1.8 | 1.55 | 0.252 | 0.195 | 0.342 | 0.88 | FALSE | FALSE | FALSE |
| **PRPF8** | PRPF8 protein | 0.968 | 1.65 | -0.684 | 0.413 | 0.261 | 0.614 | FALSE | FALSE | FALSE |
| **PRPS1L1** | Ribose-phosphate diphosphokinase | 0.384 | 0.779 | -0.395 | 0.727 | 0.517 | 0.755 | FALSE | FALSE | FALSE |

| | | | | | | | | | | |
|---|---|---|---|---|---|---|---|---|---|---|
| **PRPSAP1** | Phosphoribosyl pyrophosphate synthase-associated protein 1 | -0.741 | 1.07 | -1.81 | 0.474 | 0.363 | 0.113 | FALSE | FALSE | FALSE |
| **PRRC2A** | Proline rich coiled-coil 2A | -1.19 | -1.44 | 0.247 | 0.284 | 0.284 | 0.857 | FALSE | FALSE | FALSE |
| **PRSS8** | Serine protease 8 | 0.303 | -1.74 | 2.05 | 0.888 | 0.388 | 0.277 | FALSE | FALSE | FALSE |
| **PRTN3** | Proteinase 3 | -6.98 | -3.35 | -3.63 | 0.00097 | 0.197 | 0.0739 | TRUE | FALSE | FALSE |
| **PRXL2A** | Peroxiredoxin-like 2A | -0.29 | 0.331 | -0.621 | 0.869 | 0.867 | 0.72 | FALSE | FALSE | FALSE |
| **PRXL2B** | Prostamide/prostaglandin F synthase | -1.29 | -1.02 | -0.273 | 0.472 | 0.642 | 0.9 | FALSE | FALSE | FALSE |
| **PSAP** | Prosaposin | 0.407 | -0.539 | 0.946 | 0.729 | 0.69 | 0.421 | FALSE | FALSE | FALSE |
| **PSAT1** | Phosphoserine aminotransferase | 1.97 | -0.685 | 2.66 | 0.439 | 0.851 | 0.331 | FALSE | FALSE | FALSE |
| **PSMA1** | Proteasome subunit alpha type-1 | 1.99 | 0.704 | 1.28 | 0.107 | 0.647 | 0.339 | FALSE | FALSE | FALSE |
| **PSMA2** | Proteasome subunit alpha type-2 | 0.277 | -0.0551 | 0.332 | 0.801 | 0.967 | 0.796 | FALSE | FALSE | FALSE |
| **PSMA3** | Proteasome subunit alpha type-3 | 0.412 | 0.795 | -0.382 | 0.772 | 0.629 | 0.825 | FALSE | FALSE | FALSE |
| **PSMA4** | Proteasome subunit alpha type-4 | 1.22 | 0.225 | 0.997 | 0.262 | 0.883 | 0.395 | FALSE | FALSE | FALSE |
| **PSMA5** | Proteasome subunit alpha type-5 | -0.0805 | -0.00704 | -0.0735 | 0.94 | 0.996 | 0.957 | FALSE | FALSE | FALSE |

| | | | | | | | | | | |
|---|---|---|---|---|---|---|---|---|---|---|
| **PSMA6** | Proteasome subunit alpha type-6 | 1.77 | 0.735 | 1.03 | 0.198 | 0.662 | 0.489 | FALSE | FALSE | FALSE |
| **PSMA7** | Proteasome subunit alpha type-7 | 0.0824 | 0.527 | -0.445 | 0.944 | 0.716 | 0.756 | FALSE | FALSE | FALSE |
| **PSMB1** | Proteasome subunit beta type-1 | -0.847 | -0.503 | -0.344 | 0.421 | 0.69 | 0.798 | FALSE | FALSE | FALSE |
| **PSMB10** | Proteasome subunit beta typle-10 | -0.514 | 0.0918 | -0.606 | 0.674 | 0.956 | 0.654 | FALSE | FALSE | FALSE |
| **PSMB3** | Proteasome subunit beta type-3 | -0.163 | -0.0224 | -0.14 | 0.9 | 0.991 | 0.92 | FALSE | FALSE | FALSE |
| **PSMB4** | Proteasome subunit beta type-4 | 0.0168 | 0.32 | -0.303 | 0.99 | 0.815 | 0.819 | FALSE | FALSE | FALSE |
| **PSMB6** | Proteasome subunit beta type-6 | -0.706 | 0.74 | -1.45 | 0.625 | 0.66 | 0.327 | FALSE | FALSE | FALSE |
| **PSMB8** | Proteasome subunit beta type-8 | -0.462 | 0.18 | -0.642 | 0.731 | 0.927 | 0.663 | FALSE | FALSE | FALSE |
| **PSMB9** | Proteasome subunit beta type-9 | -0.589 | 0.52 | -1.11 | 0.689 | 0.773 | 0.469 | FALSE | FALSE | FALSE |
| **PSMC1** | Proteasome (Prosome macropain) 26S subunit | -0.779 | 0.13 | -0.909 | 0.474 | 0.936 | 0.43 | FALSE | FALSE | FALSE |
| **PSMC2** | 26S proteasome regulatory subunit 7 | 0.547 | 0.406 | 0.141 | 0.662 | 0.794 | 0.919 | FALSE | FALSE | FALSE |
| **PSMC3** | Proteasome 26S subunit ATPase 3 | 0.935 | 0.302 | 0.633 | 0.425 | 0.855 | 0.642 | FALSE | FALSE | FALSE |

| | | | | | | | | | | |
|---|---|---|---|---|---|---|---|---|---|---|
| **PSMC4** | 26S proteasome regulatory subunit 6B | -0.78 | -0.162 | -0.618 | 0.501 | 0.928 | 0.642 | FALSE | FALSE | FALSE |
| **PSMC5** | 26S proteasome regulatory subunit 8 | -0.347 | -0.113 | -0.234 | 0.822 | 0.955 | 0.887 | FALSE | FALSE | FALSE |
| **PSMC6** | 26S proteasome regulatory subunit 10B | 0.773 | 0.317 | 0.456 | 0.51 | 0.846 | 0.744 | FALSE | FALSE | FALSE |
| **PSMD1** | 26S proteasome non-ATPase regulatory subunit 1 | 1.42 | -0.682 | 2.1 | 0.239 | 0.644 | 0.116 | FALSE | FALSE | FALSE |
| **PSMD11** | 26S proteasome non-ATPase regulatory subunit 11 | -0.343 | 0.585 | -0.928 | 0.797 | 0.69 | 0.482 | FALSE | FALSE | FALSE |
| **PSMD12** | 26S proteasome non-ATPase regulatory subunit 12 | -1.31 | -1.11 | -0.208 | 0.422 | 0.572 | 0.915 | FALSE | FALSE | FALSE |
| **PSMD13** | 26S proteasome non-ATPase regulatory subunit 13 | -1.53 | -0.772 | -0.755 | 0.221 | 0.608 | 0.592 | FALSE | FALSE | FALSE |
| **PSMD2** | 26S proteasome non-ATPase regulatory subunit 2 | -0.672 | -1.45 | 0.775 | 0.66 | 0.384 | 0.643 | FALSE | FALSE | FALSE |

| | | | | | | | | | | |
|---|---|---|---|---|---|---|---|---|---|---|
| **PSMD5** | 26S proteasome non-ATPase regulatory subunit 5 | -0.844 | -1.03 | 0.184 | 0.383 | 0.361 | 0.875 | FALSE | FALSE | FALSE |
| **PSME1** | Proteasome (Prosome macropain) activator subunit 1 (PA28 alpha) | 0.402 | 0.0749 | 0.327 | 0.74 | 0.964 | 0.825 | FALSE | FALSE | FALSE |
| **PSME2** | Proteasome activator complex subunit 2 | 1.65 | 0.225 | 1.42 | 0.2 | 0.903 | 0.309 | FALSE | FALSE | FALSE |
| **PSMG2** | Proteasome assembly chaperone 2 | -0.309 | 0.0129 | -0.322 | 0.777 | 0.993 | 0.808 | FALSE | FALSE | FALSE |
| **PTBP1** | Polypyrimidine tract-binding protein 1 | 4.07 | 2.41 | 1.66 | 0.00673 | 0.232 | 0.277 | TRUE | FALSE | FALSE |
| **PTGES3** | Prostaglandin E synthase 3 | 0.421 | -0.132 | 0.553 | 0.789 | 0.953 | 0.757 | FALSE | FALSE | FALSE |
| **PTGFRN** | Prostaglandin F2 receptor inhibitor | 1.58 | 2.72 | -1.14 | 0.421 | 0.261 | 0.613 | FALSE | FALSE | FALSE |
| **PTGR1** | Prostaglandin reductase 1 | -3.75 | -3.16 | -0.593 | 0.0512 | 0.236 | 0.815 | FALSE | FALSE | FALSE |
| **PTGR2** | Prostaglandin reductase 2 | -0.176 | -0.817 | 0.642 | 0.89 | 0.496 | 0.583 | FALSE | FALSE | FALSE |
| **PTI** | Pancreatic trypsin inhibitor | -3.07 | -1.26 | -1.81 | 0.0245 | 0.417 | 0.229 | TRUE | FALSE | FALSE |
| **PTK7** | Protein tyrosine kinase 7 (inactive) | -0.522 | 0.116 | -0.638 | 0.609 | 0.936 | 0.559 | FALSE | FALSE | FALSE |

| | | | | | | | | | | |
|---|---|---|---|---|---|---|---|---|---|---|
| **PTPA** | Serine/threonine-protein phosphatase 2A activator | -0.849 | -0.85 | 0.000525 | 0.58 | 0.639 | 1 | FALSE | FALSE | FALSE |
| **PTPN6** | Tyrosine-protein phosphatase non-receptor type | -0.99 | -1.38 | 0.387 | 0.488 | 0.393 | 0.836 | FALSE | FALSE | FALSE |
| **PTPRC** | Protein-tyrosine-phosphatase | -2.15 | -2.93 | 0.78 | 0.289 | 0.256 | 0.752 | FALSE | FALSE | FALSE |
| **PTPRJ** | Peptidylprolyl isomerase | -1.22 | -1.75 | 0.533 | 0.231 | 0.224 | 0.654 | FALSE | FALSE | FALSE |
| **PTPRS** | Protein-tyrosine-phosphatase | -0.142 | 0.816 | -0.958 | 0.909 | 0.582 | 0.471 | FALSE | FALSE | FALSE |
| **PTTG1IP** | PTTG1 interacting protein | 1.76 | -0.189 | 1.95 | 0.0846 | 0.901 | 0.0907 | FALSE | FALSE | FALSE |
| **PTX3** | Pentraxin-related protein PTX3 | -5.36 | -2.15 | -3.21 | 0.000678 | 0.232 | 0.0314 | TRUE | FALSE | TRUE |
| **PURA** | Purine rich element binding protein A | 1.24 | 0.698 | 0.539 | 0.195 | 0.538 | 0.623 | FALSE | FALSE | FALSE |
| **PURB** | Purine rich element binding protein B | 2.63 | 2.21 | 0.421 | 0.0583 | 0.236 | 0.819 | FALSE | FALSE | FALSE |
| **PXDN** | Peroxidasin | 0.59 | 2.49 | -1.9 | 0.759 | 0.265 | 0.331 | FALSE | FALSE | FALSE |
| **PYCARD** | Apoptosis-associated speck-like protein containing a CARD | -0.453 | 0.932 | -1.39 | 0.762 | 0.587 | 0.359 | FALSE | FALSE | FALSE |
| **PYCR3** | Pyrroline-5-carboxylate reductase 3 | 1.45 | 0.018 | 1.44 | 0.249 | 0.993 | 0.29 | FALSE | FALSE | FALSE |

| | | | | | | | | | | |
|---|---|---|---|---|---|---|---|---|---|---|
| **PYGB** | Glycogen phosphorylase brain form | 0.158 | 2.66 | -2.5 | 0.905 | 0.156 | 0.0797 | FALSE | FALSE | FALSE |
| **PYGL** | Glycogen phosphorylase liver form | -5.32 | -0.903 | -4.42 | 0.0334 | 0.784 | 0.116 | TRUE | FALSE | FALSE |
| **QARS1** | Glutamine-tRNA ligase | 2.34 | 1.01 | 1.34 | 0.172 | 0.633 | 0.471 | FALSE | FALSE | FALSE |
| **QDPR** | Dihydropteridine reductase | -0.951 | -0.82 | -0.132 | 0.369 | 0.499 | 0.915 | FALSE | FALSE | FALSE |
| **QSOX1** | Sulfhydryl oxidase | -0.737 | -0.943 | 0.206 | 0.51 | 0.455 | 0.876 | FALSE | FALSE | FALSE |
| **RAB10** | RAB10 protein | 0.404 | -1.15 | 1.55 | 0.758 | 0.416 | 0.248 | FALSE | FALSE | FALSE |
| **RAB11B** | Ras-related protein Rab-11B | 0.616 | -1.23 | 1.85 | 0.542 | 0.289 | 0.0881 | FALSE | FALSE | FALSE |
| **RAB13** | Ras-related protein Rab-13 | 0.158 | 1.06 | -0.898 | 0.9 | 0.416 | 0.469 | FALSE | FALSE | FALSE |
| **RAB14** | Ras-related protein Rab-14 | -0.278 | -1.52 | 1.25 | 0.862 | 0.326 | 0.372 | FALSE | FALSE | FALSE |
| **RAB18** | Ras-related protein Rab-18 | 0.443 | 0.348 | 0.0946 | 0.688 | 0.802 | 0.939 | FALSE | FALSE | FALSE |
| **RAB1A** | RAB1A member RAS onco family | 1.99 | -0.634 | 2.63 | 0.0653 | 0.639 | 0.037 | FALSE | FALSE | TRUE |
| **RAB1B** | Ras-related protein Rab-1B | -0.0466 | -0.866 | 0.82 | 0.967 | 0.469 | 0.469 | FALSE | FALSE | FALSE |
| **RAB21** | Ras-related protein Rab-21 | -2.54 | -1.51 | -1.04 | 0.0343 | 0.298 | 0.423 | TRUE | FALSE | FALSE |
| **RAB23** | RAB23, member RAS onco family | -1.4 | 0.156 | -1.56 | 0.319 | 0.94 | 0.302 | FALSE | FALSE | FALSE |
| **RAB25** | Ras-related protein Rab-25 | -1.03 | -1.55 | 0.526 | 0.488 | 0.363 | 0.773 | FALSE | FALSE | FALSE |

| | | | | | | | | | | |
|---|---|---|---|---|---|---|---|---|---|---|
| **RAB27B** | Ras-related protein Rab-27B | 1.79 | -0.105 | 1.9 | 0.134 | 0.955 | 0.159 | FALSE | FALSE | FALSE |
| **RAB2A** | RAB2A, member RAS onco family | 0.334 | -0.827 | 1.16 | 0.801 | 0.565 | 0.362 | FALSE | FALSE | FALSE |
| **RAB2A.1** | RAB2A, member RAS onco family | -0.951 | -0.712 | -0.239 | 0.332 | 0.529 | 0.848 | FALSE | FALSE | FALSE |
| **RAB31** | RAB31, member RAS onco family | -0.324 | -0.476 | 0.152 | 0.751 | 0.689 | 0.898 | FALSE | FALSE | FALSE |
| **RAB35** | RAB35 protein | 0.657 | -1.37 | 2.02 | 0.684 | 0.431 | 0.229 | FALSE | FALSE | FALSE |
| **RAB3D** | Ras-related protein Rab-3 | 1.82 | 0.165 | 1.66 | 0.0844 | 0.919 | 0.169 | FALSE | FALSE | FALSE |
| **RAB5B** | RAB5B, member RAS onco family | -1.25 | -1.86 | 0.604 | 0.28 | 0.236 | 0.654 | FALSE | FALSE | FALSE |
| **RAB5C** | Ras-related protein Rab-5C | -0.668 | -1.29 | 0.624 | 0.56 | 0.326 | 0.62 | FALSE | FALSE | FALSE |
| **RAB6A** | RAB6A, member RAS onco family | 0.311 | -1.39 | 1.7 | 0.761 | 0.257 | 0.116 | FALSE | FALSE | FALSE |
| **RAB7A** | Ras-related protein Rab-7a | -0.207 | -0.991 | 0.784 | 0.851 | 0.363 | 0.431 | FALSE | FALSE | FALSE |
| **RAB8A** | Ras-related protein Rab-8A | 0.879 | -1.67 | 2.55 | 0.501 | 0.279 | 0.0797 | FALSE | FALSE | FALSE |
| **RAB8B** | Ras-related protein Rab-8B | -0.178 | -0.654 | 0.475 | 0.895 | 0.66 | 0.746 | FALSE | FALSE | FALSE |
| **RABEP1** | Rabaptin, RAB GTPase binding effector protein 1 | -1.17 | -0.563 | -0.606 | 0.382 | 0.737 | 0.704 | FALSE | FALSE | FALSE |
| **RABEP2** | Rab GTPase-binding effector protein 2 | -1.28 | -0.821 | -0.46 | 0.322 | 0.599 | 0.774 | FALSE | FALSE | FALSE |
| **RAC1** | Ras-related C3 botulinum toxin substrate 1 | -0.379 | -1.39 | 1.01 | 0.723 | 0.265 | 0.347 | FALSE | FALSE | FALSE |

| | | | | | | | | | | |
|---|---|---|---|---|---|---|---|---|---|---|
| **RAC2** | Ras-related C3 botulinum toxin substrate 2 | -1.42 | -2.11 | 0.686 | 0.157 | 0.16 | 0.549 | FALSE | FALSE | FALSE |
| **RACK1** | Receptor of activated protein C kinase 1 | 2.72 | 1.29 | 1.42 | 0.0392 | 0.393 | 0.323 | TRUE | FALSE | FALSE |
| **RAD23B** | UV excision repair protein RAD23 | -1.01 | -0.247 | -0.763 | 0.354 | 0.867 | 0.521 | FALSE | FALSE | FALSE |
| **RAD50** | RAD50 double strand break repair protein | -0.465 | -0.274 | -0.191 | 0.677 | 0.854 | 0.879 | FALSE | FALSE | FALSE |
| **RAD54L2** | RAD54 like 2 | 0.175 | -0.233 | 0.408 | 0.89 | 0.87 | 0.744 | FALSE | FALSE | FALSE |
| **RALA** | Small monomeric GTPase | -0.508 | -0.247 | -0.261 | 0.689 | 0.883 | 0.858 | FALSE | FALSE | FALSE |
| **RALB** | Small monomeric GTPase | 1.43 | -0.579 | 2.01 | 0.228 | 0.69 | 0.129 | FALSE | FALSE | FALSE |
| **RALY** | RALY heterous nuclear ribonucleoprotein | 0.247 | 0.745 | -0.498 | 0.865 | 0.606 | 0.73 | FALSE | FALSE | FALSE |
| **RAN** | GTP-binding nuclear protein Ran | 1.54 | -0.107 | 1.65 | 0.244 | 0.956 | 0.249 | FALSE | FALSE | FALSE |
| **RAP1A** | Ras-related protein Rap-1A | 0.399 | -0.881 | 1.28 | 0.728 | 0.477 | 0.274 | FALSE | FALSE | FALSE |
| **RAP1B** | Ras-related protein Rap-1b | 0.0766 | -0.67 | 0.746 | 0.951 | 0.651 | 0.584 | FALSE | FALSE | FALSE |
| **RAP2B** | Ras-related protein Rap-2 | 0.158 | 1.65 | -1.5 | 0.905 | 0.285 | 0.282 | FALSE | FALSE | FALSE |
| **RAP2C** | Ras-related protein Rap-2c | 1.28 | -0.1 | 1.38 | 0.243 | 0.952 | 0.243 | FALSE | FALSE | FALSE |

| | | | | | | | | | | |
|---|---|---|---|---|---|---|---|---|---|---|
| **RARRES1** | Retinoic acid receptor responder 1 cytoplasmic | 1.13 | -0.563 | 1.69 | 0.444 | 0.763 | 0.282 | FALSE | FALSE | FALSE |
| **RARS1** | Arginine--tRNA ligase | 2 | 0.105 | 1.9 | 0.3 | 0.967 | 0.354 | FALSE | FALSE | FALSE |
| **RASAL1** | RAS protein activator like 1 | -0.942 | -0.734 | -0.208 | 0.389 | 0.574 | 0.875 | FALSE | FALSE | FALSE |
| **RBBP7** | Histone-binding protein RBBP7 | 0.353 | 0.39 | -0.0364 | 0.838 | 0.852 | 0.985 | FALSE | FALSE | FALSE |
| **RBM39** | RNA binding motif protein 39 | -0.79 | -0.508 | -0.282 | 0.434 | 0.684 | 0.83 | FALSE | FALSE | FALSE |
| **RBM4** | RNA-binding protein 4 | 0.246 | 0.784 | -0.539 | 0.886 | 0.644 | 0.748 | FALSE | FALSE | FALSE |
| **RBM47** | RNA-binding protein 47 | 1.65 | 1.34 | 0.313 | 0.51 | 0.66 | 0.915 | FALSE | FALSE | FALSE |
| **RBMX** | RNA-binding motif protein X chromosome | 0.0705 | 0.479 | -0.408 | 0.954 | 0.749 | 0.782 | FALSE | FALSE | FALSE |
| **RBP4** | Retinol-binding protein 4 | 0.158 | 1.39 | -1.23 | 0.895 | 0.279 | 0.282 | FALSE | FALSE | FALSE |
| **RCC2** | RCC2 protein | 1.36 | -0.0343 | 1.39 | 0.3 | 0.985 | 0.325 | FALSE | FALSE | FALSE |
| **RDH11** | Retinol dehydrogenase 11 | -3.21 | -2.34 | -0.877 | 0.146 | 0.363 | 0.744 | FALSE | FALSE | FALSE |
| **RDX** | Radixin | 3.04 | -0.626 | 3.67 | 0.0743 | 0.781 | 0.0647 | FALSE | FALSE | FALSE |
| **REPS1** | RALBP1 associated Eps domain containing 1 | -0.284 | 2.63 | -2.92 | 0.864 | 0.186 | 0.0629 | FALSE | FALSE | FALSE |
| **RETN** | Resistin | -1.49 | -2.63 | 1.14 | 0.164 | 0.0943 | 0.327 | FALSE | FALSE | FALSE |
| **RHEB** | Uncharacterized protein | -0.596 | -0.332 | -0.264 | 0.569 | 0.804 | 0.841 | FALSE | FALSE | FALSE |

| | | | | | | | | | | |
|---|---|---|---|---|---|---|---|---|---|---|
| **RHOA** | Transforming protein RhoA | -0.776 | -1.5 | 0.719 | 0.486 | 0.265 | 0.56 | FALSE | FALSE | FALSE |
| **RHOB** | Rho-related GTP-binding protein RhoB | -0.478 | 0.148 | -0.626 | 0.708 | 0.936 | 0.658 | FALSE | FALSE | FALSE |
| **RHOC** | Rho-related GTP-binding protein RhoC | -0.538 | -1.61 | 1.07 | 0.714 | 0.329 | 0.475 | FALSE | FALSE | FALSE |
| **RHOG** | Ras homolog family member G | -1.88 | -1.89 | 0.00844 | 0.0743 | 0.214 | 0.994 | FALSE | FALSE | FALSE |
| **RHPN2** | Rhophilin-2 | 0.185 | -2.49 | 2.67 | 0.917 | 0.265 | 0.185 | FALSE | FALSE | FALSE |
| **RIPOR1** | RHO family interacting cell polarization regulator 1 | -0.71 | -2.68 | 1.97 | 0.708 | 0.253 | 0.31 | FALSE | FALSE | FALSE |
| **RNH1** | Ribonuclease inhibitor | 0.334 | -0.949 | 1.28 | 0.885 | 0.676 | 0.533 | FALSE | FALSE | FALSE |
| **RNPEP** | Arginyl aminopeptidase | -0.435 | -0.972 | 0.537 | 0.743 | 0.502 | 0.721 | FALSE | FALSE | FALSE |
| **RO60** | Ro60 Y RNA binding protein | 0.532 | 0.41 | 0.123 | 0.63 | 0.76 | 0.92 | FALSE | FALSE | FALSE |
| **ROCK1** | Non-specific serine/threonine protein kinase | -1.28 | -0.181 | -1.1 | 0.307 | 0.927 | 0.412 | FALSE | FALSE | FALSE |
| **ROCK2** | Rho-associated protein kinase 2 | -1.57 | 0.272 | -1.84 | 0.304 | 0.903 | 0.267 | FALSE | FALSE | FALSE |
| **RP2** | Protein XRP2 | 0.481 | -0.412 | 0.894 | 0.656 | 0.749 | 0.41 | FALSE | FALSE | FALSE |
| **RPL10** | 60S ribosomal protein L10 | 5.13 | 1.75 | 3.38 | 0.00373 | 0.344 | 0.0646 | TRUE | FALSE | FALSE |
| **RPL10A** | 60S ribosomal protein L10a | 3.98 | 2.89 | 1.09 | 0.00876 | 0.186 | 0.497 | TRUE | FALSE | FALSE |

| | | | | | | | | | | |
|---|---|---|---|---|---|---|---|---|---|---|
| **RPL11** | 60S ribosomal protein L11 | 6.54 | 1.57 | 4.97 | 0.000411 | 0.361 | 0.00704 | TRUE | FALSE | TRUE |
| **RPL12** | 60S ribosomal protein L12 | 6.73 | 1.69 | 5.04 | 0.000855 | 0.382 | 0.0139 | TRUE | FALSE | TRUE |
| **RPL13** | 60S ribosomal protein L13 | 3.24 | 1.91 | 1.33 | 0.0125 | 0.253 | 0.332 | TRUE | FALSE | FALSE |
| **RPL13A** | 60S ribosomal protein L13a | 3.49 | 1.43 | 2.06 | 0.00839 | 0.342 | 0.146 | TRUE | FALSE | FALSE |
| **RPL14** | 60S ribosomal protein L14 | 7.02 | 1.83 | 5.18 | 0.000892 | 0.365 | 0.0154 | TRUE | FALSE | TRUE |
| **RPL14.1** | 60S ribosomal protein L14 | 2.11 | 1.52 | 0.585 | 0.144 | 0.363 | 0.742 | FALSE | FALSE | FALSE |
| **RPL15** | 60S ribosomal protein L15 | 7.11 | 1.94 | 5.16 | 0.000309 | 0.285 | 0.00704 | TRUE | FALSE | TRUE |
| **RPL17** | 60S ribosomal protein L17 | 6.24 | 2.67 | 3.57 | 0.00128 | 0.236 | 0.0629 | TRUE | FALSE | FALSE |
| **RPL18** | 60S ribosomal protein L18 | 6.99 | 1.06 | 5.93 | 0.0002 | 0.512 | 0.00186 | TRUE | FALSE | TRUE |
| **RPL18A** | 60S ribosomal protein L18a | 6.75 | 0.971 | 5.78 | 9.68E-05 | 0.467 | 0.000811 | TRUE | FALSE | TRUE |
| **RPL19** | 60S ribosomal protein L19 | 2.58 | 2.23 | 0.358 | 0.0263 | 0.186 | 0.815 | TRUE | FALSE | FALSE |
| **RPL21** | 60S ribosomal protein L21 (Fragment) | 5.17 | 1.47 | 3.7 | 0.00197 | 0.387 | 0.0294 | TRUE | FALSE | TRUE |
| **RPL22** | Ribosomal protein L22 | 3.31 | 1.85 | 1.46 | 0.0551 | 0.363 | 0.434 | FALSE | FALSE | FALSE |
| **RPL23** | 60S ribosomal protein L23 | 5.93 | 2.61 | 3.32 | 0.000282 | 0.155 | 0.0227 | TRUE | FALSE | TRUE |
| **RPL23A** | 60S ribosomal protein L23a | 4.76 | 3.03 | 1.73 | 0.00398 | 0.176 | 0.282 | TRUE | FALSE | FALSE |

| | | | | | | | | | | |
|---|---|---|---|---|---|---|---|---|---|---|
| **RPL24** | 60S ribosomal protein L24 | 6.24 | 2 | 4.23 | 0.000888 | 0.285 | 0.0223 | TRUE | FALSE | TRUE |
| **RPL26** | 60S ribosomal protein L26 | 6.83 | 1.94 | 4.89 | 0.000282 | 0.268 | 0.00704 | TRUE | FALSE | TRUE |
| **RPL27** | 60S ribosomal protein L27 | 6.85 | 2.02 | 4.83 | 0.000309 | 0.265 | 0.00816 | TRUE | FALSE | TRUE |
| **RPL27A** | 60S ribosomal protein L27a | 7.48 | 1.94 | 5.54 | 4.91E-05 | 0.217 | 0.000811 | TRUE | FALSE | TRUE |
| **RPL28** | 60S ribosomal protein L28 | 4.08 | 1.99 | 2.08 | 0.00389 | 0.236 | 0.145 | TRUE | FALSE | FALSE |
| **RPL29** | 60S ribosomal protein L29 | 4.48 | 2.04 | 2.44 | 0.00498 | 0.261 | 0.139 | TRUE | FALSE | FALSE |
| **RPL3** | 60S ribosomal protein L3 | 8.36 | 2.46 | 5.9 | 0.0002 | 0.253 | 0.00504 | TRUE | FALSE | TRUE |
| **RPL30** | 60S ribosomal protein L30 | 4.58 | 2.06 | 2.53 | 0.00673 | 0.285 | 0.157 | TRUE | FALSE | FALSE |
| **RPL31** | 60S ribosomal protein L31 | 5.93 | 2.3 | 3.62 | 0.00499 | 0.323 | 0.0919 | TRUE | FALSE | FALSE |
| **RPL32** | 60S ribosomal protein L32 | 3.13 | 1.76 | 1.37 | 0.0608 | 0.365 | 0.455 | FALSE | FALSE | FALSE |
| **RPL34** | 60S ribosomal protein L34 | 4.46 | 1.95 | 2.51 | 0.0013 | 0.236 | 0.0669 | TRUE | FALSE | FALSE |
| **RPL35** | 60S ribosomal protein L35 | 4.42 | 0.918 | 3.5 | 0.00546 | 0.606 | 0.037 | TRUE | FALSE | TRUE |
| **RPL35A** | 60S ribosomal protein L35a | 3.56 | 1.86 | 1.7 | 0.0108 | 0.265 | 0.249 | TRUE | FALSE | FALSE |
| **RPL36** | 60S ribosomal protein L36 | 2.66 | 2.46 | 0.198 | 0.125 | 0.261 | 0.921 | FALSE | FALSE | FALSE |
| **RPL36A** | 60S ribosomal protein L36a | 3.97 | 1.46 | 2.51 | 0.00291 | 0.304 | 0.0659 | TRUE | FALSE | FALSE |

| | | | | | | | | | | |
|---|---|---|---|---|---|---|---|---|---|---|
| **RPL37A** | 60S ribosomal protein L37a | 2.39 | 2.17 | 0.224 | 0.0548 | 0.224 | 0.882 | FALSE | FALSE | FALSE |
| **RPL38** | 60S ribosomal protein L38 | 2.72 | 2.61 | 0.105 | 0.00833 | 0.0697 | 0.926 | TRUE | FALSE | FALSE |
| **RPL4** | 60S ribosomal protein L4 | 8.51 | 2.7 | 5.8 | 9.68E-05 | 0.214 | 0.00324 | TRUE | FALSE | TRUE |
| **RPL5** | 60S ribosomal protein L5 | 7.11 | 3.45 | 3.66 | 0.00413 | 0.246 | 0.146 | TRUE | FALSE | FALSE |
| **RPL6** | 60S ribosomal protein L6 | 7.65 | 2.6 | 5.04 | 0.000324 | 0.236 | 0.013 | TRUE | FALSE | TRUE |
| **RPL7** | 60S ribosomal protein L7 | 5.73 | 1.58 | 4.15 | 0.00103 | 0.361 | 0.0192 | TRUE | FALSE | TRUE |
| **RPL7A** | 60S ribosomal protein L7a | 7.68 | 1.8 | 5.88 | 9.33E-05 | 0.261 | 0.00124 | TRUE | FALSE | TRUE |
| **RPL8** | 60S ribosomal protein L8 | 7.42 | 1.96 | 5.46 | 4.91E-05 | 0.214 | 0.000811 | TRUE | FALSE | TRUE |
| **RPL9** | 60S ribosomal protein L9 | 3.78 | 2.13 | 1.65 | 0.00943 | 0.253 | 0.277 | TRUE | FALSE | FALSE |
| **RPLP0** | 60S acidic ribosomal protein P0 | 3.83 | 1.57 | 2.26 | 0.00673 | 0.323 | 0.129 | TRUE | FALSE | FALSE |
| **RPLP1** | 60S acidic ribosomal protein P1 | 2.34 | 0.806 | 1.53 | 0.108 | 0.659 | 0.332 | FALSE | FALSE | FALSE |
| **RPLP2** | 60S acidic ribosomal protein P2 | 3.04 | 1.42 | 1.62 | 0.0341 | 0.393 | 0.295 | TRUE | FALSE | FALSE |

| | | | | | | | | | | |
|---|---|---|---|---|---|---|---|---|---|---|
| **RPN1** | Dolichyl-diphosphooligosaccharide--protein glycosyltransferase subunit 1 | 0.941 | 1.28 | -0.335 | 0.44 | 0.363 | 0.836 | FALSE | FALSE | FALSE |
| **RPS10** | 40S ribosomal protein S10 | -0.781 | 0.181 | -0.962 | 0.614 | 0.936 | 0.561 | FALSE | FALSE | FALSE |
| **RPS11** | 40S ribosomal protein S11 | 7.39 | 1.67 | 5.72 | 9.68E-05 | 0.292 | 0.00186 | TRUE | FALSE | TRUE |
| **RPS12** | 40S ribosomal protein S12 | 0.491 | 0.0641 | 0.427 | 0.633 | 0.964 | 0.716 | FALSE | FALSE | FALSE |
| **RPS13** | 40S ribosomal protein S13 | 0.627 | 0.483 | 0.144 | 0.697 | 0.816 | 0.936 | FALSE | FALSE | FALSE |
| **RPS14** | Uncharacterized protein | 6.42 | 2.24 | 4.17 | 9.68E-05 | 0.186 | 0.00485 | TRUE | FALSE | TRUE |
| **RPS15** | 40S ribosomal protein S15 | 2.28 | 1.49 | 0.788 | 0.125 | 0.391 | 0.654 | FALSE | FALSE | FALSE |
| **RPS15A** | 40S ribosomal protein S15a | 1.97 | 0.715 | 1.26 | 0.0612 | 0.578 | 0.277 | FALSE | FALSE | FALSE |
| **RPS16** | 40S ribosomal protein S16 | 7.25 | 1.11 | 6.13 | 9.68E-05 | 0.431 | 0.000811 | TRUE | FALSE | TRUE |
| **RPS17** | 40S ribosomal protein S17 | 1.81 | 1.22 | 0.595 | 0.102 | 0.356 | 0.654 | FALSE | FALSE | FALSE |
| **RPS18** | 40S ribosomal protein S18 | 1.47 | 0.493 | 0.976 | 0.2 | 0.734 | 0.428 | FALSE | FALSE | FALSE |
| **RPS19** | 40S ribosomal protein S19 | 2.24 | 2.15 | 0.094 | 0.139 | 0.26 | 0.965 | FALSE | FALSE | FALSE |
| **RPS2** | 40S ribosomal protein S2 | 7.98 | 1.26 | 6.72 | 4.91E-05 | 0.344 | 0.000811 | TRUE | FALSE | TRUE |
| **RPS20** | 40S ribosomal protein S20 | 2.53 | 0.965 | 1.57 | 0.0444 | 0.513 | 0.254 | TRUE | FALSE | FALSE |

| | | | | | | | | | | |
|---|---|---|---|---|---|---|---|---|---|---|
| **RPS21** | 40S ribosomal protein S21 | -0.479 | 0.41 | -0.889 | 0.676 | 0.765 | 0.444 | FALSE | FALSE | FALSE |
| **RPS23** | 40S ribosomal protein S23 | 4.62 | 1.43 | 3.19 | 0.00576 | 0.417 | 0.0726 | TRUE | FALSE | FALSE |
| **RPS24** | 40S ribosomal protein S24 | 7.22 | 1.68 | 5.54 | 9.68E-05 | 0.285 | 0.00193 | TRUE | FALSE | TRUE |
| **RPS25** | 40S ribosomal protein S25 | 7.15 | 2.21 | 4.94 | 0.00185 | 0.344 | 0.0323 | TRUE | FALSE | TRUE |
| **RPS26** | 40S ribosomal protein S26 | 5.26 | 1.13 | 4.12 | 0.000892 | 0.45 | 0.0115 | TRUE | FALSE | TRUE |
| **RPS27A** | Ubiquitin-40S ribosomal protein S27a | 0.849 | -0.256 | 1.11 | 0.376 | 0.849 | 0.277 | FALSE | FALSE | FALSE |
| **RPS27L** | 40S ribosomal protein S27-like | 4.14 | 2.33 | 1.81 | 0.00123 | 0.155 | 0.139 | TRUE | FALSE | FALSE |
| **RPS3** | 40S ribosomal protein S3 | 6.17 | 1.12 | 5.05 | 0.000282 | 0.446 | 0.00303 | TRUE | FALSE | TRUE |
| **RPS3A** | 40S ribosomal protein S3a | 6.75 | 2.1 | 4.65 | 0.000849 | 0.291 | 0.02 | TRUE | FALSE | TRUE |
| **RPS4** | 40S ribosomal protein S4 | 4.8 | 0.0876 | 4.71 | 0.0152 | 0.973 | 0.037 | TRUE | FALSE | TRUE |
| **RPS4Y1** | 40S ribosomal protein S4 | 0.164 | -0.17 | 0.333 | 0.895 | 0.919 | 0.816 | FALSE | FALSE | FALSE |
| **RPS5** | 40S ribosomal protein S5 | 1.19 | -0.51 | 1.7 | 0.376 | 0.763 | 0.239 | FALSE | FALSE | FALSE |
| **RPS6** | 40S ribosomal protein S6 | 7.11 | 2.16 | 4.95 | 0.000282 | 0.256 | 0.00778 | TRUE | FALSE | TRUE |
| **RPS6KA1** | Ribosomal protein S6 kinase | 0.509 | 0.496 | 0.0129 | 0.68 | 0.736 | 0.991 | FALSE | FALSE | FALSE |
| **RPS6KA5** | Ribosomal protein S6 kinase | -1.17 | -1.42 | 0.247 | 0.28 | 0.279 | 0.856 | FALSE | FALSE | FALSE |

| | | | | | | | | | | |
|---|---|---|---|---|---|---|---|---|---|---|
| **RPS7** | 40S ribosomal protein S7 | 1.45 | 1.21 | 0.232 | 0.413 | 0.565 | 0.913 | FALSE | FALSE | FALSE |
| **RPS8** | 40S ribosomal protein S8 | 7.38 | 1.89 | 5.49 | 9.68E-05 | 0.257 | 0.00207 | TRUE | FALSE | TRUE |
| **RPS9** | 40S ribosomal protein S9 | 3.21 | 0.95 | 2.26 | 0.00877 | 0.487 | 0.0873 | TRUE | FALSE | FALSE |
| **RPSA** | 40S ribosomal protein SA | 1.09 | -0.635 | 1.72 | 0.426 | 0.7 | 0.243 | FALSE | FALSE | FALSE |
| **RPTN** | Repetin | -1.15 | -0.281 | -0.873 | 0.501 | 0.913 | 0.657 | FALSE | FALSE | FALSE |
| **RRAS** | RAS related | -0.908 | -0.0522 | -0.856 | 0.55 | 0.978 | 0.611 | FALSE | FALSE | FALSE |
| **RRAS2** | RAS related 2 | 0.00351 | -0.796 | 0.8 | 0.998 | 0.512 | 0.476 | FALSE | FALSE | FALSE |
| **RRBP1** | Ribosome binding protein 1 | 2.91 | 3.34 | -0.437 | 0.0499 | 0.129 | 0.824 | TRUE | FALSE | FALSE |
| **RTCA** | RNA 3-terminal phosphate cyclase | -0.443 | 0.0997 | -0.543 | 0.689 | 0.952 | 0.661 | FALSE | FALSE | FALSE |
| **RTCB** | RNA-splicing ligase RtcB homolog | 4.16 | 1.38 | 2.79 | 0.00601 | 0.393 | 0.0808 | TRUE | FALSE | FALSE |
| **RTN1** | Reticulon | -0.518 | -0.244 | -0.273 | 0.685 | 0.884 | 0.857 | FALSE | FALSE | FALSE |
| **RTN3** | Reticulon-3 | -0.879 | -0.248 | -0.631 | 0.474 | 0.883 | 0.656 | FALSE | FALSE | FALSE |
| **RUVBL1** | RuvB-like helicase | 2.95 | 1.31 | 1.63 | 0.0175 | 0.363 | 0.229 | TRUE | FALSE | FALSE |
| **RUVBL2** | RuvB-like 2 | 3.72 | 1.59 | 2.13 | 0.00839 | 0.326 | 0.161 | TRUE | FALSE | FALSE |
| **S100A10** | Protein S100-A10 | 1.74 | 1.1 | 0.635 | 0.333 | 0.606 | 0.774 | FALSE | FALSE | FALSE |
| **S100A11** | Protein S100 | -1.31 | -0.969 | -0.338 | 0.348 | 0.552 | 0.848 | FALSE | FALSE | FALSE |
| **S100A12** | Protein S100-A12 | -3.65 | -2.74 | -0.916 | 0.0446 | 0.253 | 0.668 | TRUE | FALSE | FALSE |
| **S100A2** | Protein S100-A2 | 1.37 | 0.509 | 0.857 | 0.176 | 0.687 | 0.43 | FALSE | FALSE | FALSE |
| **S100A4** | Protein S100-A4 | 0.669 | -0.0733 | 0.743 | 0.51 | 0.959 | 0.497 | FALSE | FALSE | FALSE |
| **S100A8** | Protein S100-A8 | -5.43 | -5.67 | 0.241 | 0.00375 | 0.0198 | 0.903 | TRUE | TRUE | FALSE |
| **S100A9** | Protein S100-A9 | -2.83 | -4.45 | 1.62 | 0.0863 | 0.0697 | 0.363 | FALSE | FALSE | FALSE |
| **S100G** | Protein S100-G | 1.42 | 0.327 | 1.09 | 0.362 | 0.876 | 0.52 | FALSE | FALSE | FALSE |
| **SAA1** | Serum amyloid A protein | -1.35 | -1.6 | 0.247 | 0.132 | 0.214 | 0.838 | FALSE | FALSE | FALSE |

| | | | | | | | | | | |
|---|---|---|---|---|---|---|---|---|---|---|
| **SAA3** | Serum amyloid A-3 protein | 0.754 | 1.19 | -0.44 | 0.549 | 0.393 | 0.772 | FALSE | FALSE | FALSE |
| **SAMHD1** | Deoxynucleoside triphosphate triphosphohydrolase SAMHD1 | 1.67 | 0.418 | 1.26 | 0.184 | 0.804 | 0.354 | FALSE | FALSE | FALSE |
| **SAO** | Amine oxidase | 1.6 | 1.76 | -0.164 | 0.217 | 0.265 | 0.915 | FALSE | FALSE | FALSE |
| **SARG** | Specifically androgen-regulated gene protein | -1.96 | -1.38 | -0.585 | 0.0882 | 0.326 | 0.669 | FALSE | FALSE | FALSE |
| **SARS1** | Serine-tRNA ligase cytoplasmic | -5.18 | -4.34 | -0.837 | 0.0224 | 0.186 | 0.765 | TRUE | FALSE | FALSE |
| **SART3** | Spliceosome associated factor 3 U4/U6 recycling protein | -0.0141 | 0.0167 | -0.0309 | 0.991 | 0.992 | 0.983 | FALSE | FALSE | FALSE |
| **SCAMP2** | Secretory carrier-associated membrane protein | -0.264 | 0.205 | -0.469 | 0.834 | 0.896 | 0.72 | FALSE | FALSE | FALSE |
| **SCAMP3** | Secretory carrier-associated membrane protein 3 | -0.923 | 0.386 | -1.31 | 0.446 | 0.813 | 0.318 | FALSE | FALSE | FALSE |
| **SCFD1** | Sec1 family domain containing 1 | -1.53 | -1.77 | 0.247 | 0.2 | 0.253 | 0.865 | FALSE | FALSE | FALSE |
| **SCIN** | Scinderin | -1.01 | -0.465 | -0.55 | 0.299 | 0.69 | 0.62 | FALSE | FALSE | FALSE |
| **SCPEP1** | Carboxypeptidase | 0.158 | 1.76 | -1.6 | 0.9 | 0.253 | 0.223 | FALSE | FALSE | FALSE |
| **SDCBP** | Syndecan binding protein | -0.0125 | -0.169 | 0.157 | 0.992 | 0.917 | 0.901 | FALSE | FALSE | FALSE |
| **SDF4** | 45 kDa calcium-binding protein | 0.874 | -1.1 | 1.97 | 0.613 | 0.576 | 0.268 | FALSE | FALSE | FALSE |

| | | | | | | | | | | |
|---|---|---|---|---|---|---|---|---|---|---|
| **SDS** | L-serine dehydratase/L-threonine deaminase | -6.14 | -3.66 | -2.47 | 0.00128 | 0.129 | 0.177 | TRUE | FALSE | FALSE |
| **SEC13** | Protein SEC13 homolog | 0.378 | 1.94 | -1.56 | 0.759 | 0.232 | 0.227 | FALSE | FALSE | FALSE |
| **SEC16A** | Protein transport protein sec16 | -0.524 | -0.567 | 0.0425 | 0.625 | 0.65 | 0.975 | FALSE | FALSE | FALSE |
| **SEC22B** | SEC22 homolog B vesicle trafficking protein | -1.23 | 0.487 | -1.72 | 0.45 | 0.829 | 0.327 | FALSE | FALSE | FALSE |
| **SEC23A** | Protein transport protein SEC23 | -0.596 | -0.55 | -0.0462 | 0.609 | 0.688 | 0.975 | FALSE | FALSE | FALSE |
| **SEC23B** | Protein transport protein Sec23B | 2.32 | 0.559 | 1.76 | 0.0499 | 0.7 | 0.185 | TRUE | FALSE | FALSE |
| **SEC23IP** | SEC23 interacting protein | -1.13 | -0.238 | -0.894 | 0.334 | 0.883 | 0.474 | FALSE | FALSE | FALSE |
| **SEC24C** | SEC24 homolog C COPII coat complex component | -0.641 | 0.509 | -1.15 | 0.595 | 0.72 | 0.345 | FALSE | FALSE | FALSE |
| **SEC31A** | SEC31 homolog A COPII coat complex component | 3 | 1.12 | 1.88 | 0.0392 | 0.512 | 0.239 | TRUE | FALSE | FALSE |
| **SEC61B** | Protein transport protein Sec61 subunit beta | -0.783 | -1.03 | 0.247 | 0.422 | 0.363 | 0.846 | FALSE | FALSE | FALSE |
| **SELENBP1** | Methanethiol oxidase | -0.17 | 0.569 | -0.739 | 0.892 | 0.665 | 0.541 | FALSE | FALSE | FALSE |
| **SEMA7A** | Semaphorin 7A | 1.29 | 1.1 | 0.195 | 0.428 | 0.574 | 0.919 | FALSE | FALSE | FALSE |
| **SENP3** | SUMO specific peptidase 3 | -1.23 | -0.566 | -0.667 | 0.377 | 0.749 | 0.68 | FALSE | FALSE | FALSE |

| | | | | | | | | | | |
|---|---|---|---|---|---|---|---|---|---|---|
| **SEPTIN2** | Septin-2 | 1.62 | 0.419 | 1.2 | 0.182 | 0.796 | 0.356 | FALSE | FALSE | FALSE |
| **SEPTIN5** | Septin-5 | 0.158 | 1.72 | -1.56 | 0.9 | 0.256 | 0.228 | FALSE | FALSE | FALSE |
| **SEPTIN6** | Septin | 0.909 | 0.734 | 0.175 | 0.447 | 0.612 | 0.901 | FALSE | FALSE | FALSE |
| **SEPTIN7** | Septin | 0.689 | -0.228 | 0.917 | 0.651 | 0.917 | 0.569 | FALSE | FALSE | FALSE |
| **SEPTIN9** | Septin 9 | 0.327 | 0.089 | 0.238 | 0.811 | 0.959 | 0.875 | FALSE | FALSE | FALSE |
| **SERBP1** | SERPINE1 mRNA binding protein 1 | 0.26 | 2.08 | -1.82 | 0.852 | 0.214 | 0.169 | FALSE | FALSE | FALSE |
| **SERPINA3-3** | Serpin A3-3 | -1.65 | -0.799 | -0.849 | 0.222 | 0.629 | 0.575 | FALSE | FALSE | FALSE |
| **SERPINB1** | Leukocyte elastase inhibitor | -7.37 | -4.46 | -2.92 | 0.000138 | 0.0244 | 0.0678 | TRUE | TRUE | FALSE |
| **SERPINB5** | SERPINB5 protein | -0.685 | 0.651 | -1.34 | 0.594 | 0.662 | 0.31 | FALSE | FALSE | FALSE |
| **SERPINB6** | Serpin B6 | 1.04 | -0.686 | 1.73 | 0.325 | 0.587 | 0.142 | FALSE | FALSE | FALSE |
| **SERPINE2** | Serine protease inhibitor clade E member 2 | 0.892 | 2.06 | -1.16 | 0.503 | 0.236 | 0.407 | FALSE | FALSE | FALSE |
| **SERPINH1** | Serpin H1 | 0.329 | 0.709 | -0.38 | 0.765 | 0.569 | 0.769 | FALSE | FALSE | FALSE |
| **SF3A1** | Splicing factor 3A subunit 1 | 0.179 | 0.86 | -0.681 | 0.892 | 0.512 | 0.592 | FALSE | FALSE | FALSE |
| **SF3B1** | Splicing factor 3b subunit 1 | 0.173 | 1.43 | -1.26 | 0.892 | 0.276 | 0.282 | FALSE | FALSE | FALSE |
| **SF3B2** | Splicing factor 3b subunit 2 | -2.77 | -1.77 | -1 | 0.0615 | 0.326 | 0.557 | FALSE | FALSE | FALSE |
| **SF3B3** | Splicing factor 3B subunit 3 | -1.59 | 0.0961 | -1.69 | 0.302 | 0.966 | 0.31 | FALSE | FALSE | FALSE |
| **SFN** | 14-3-3 protein sigma | 0.132 | -0.389 | 0.522 | 0.915 | 0.816 | 0.729 | FALSE | FALSE | FALSE |
| **SFPQ** | Splicing factor proline and glutamine rich | 0.0875 | 0.529 | -0.441 | 0.929 | 0.666 | 0.716 | FALSE | FALSE | FALSE |
| **SFRP1** | Secreted frizzled-related protein 1 | -1.7 | -1.95 | 0.247 | 0.0557 | 0.143 | 0.836 | FALSE | FALSE | FALSE |

| | | | | | | | | | | |
|---|---|---|---|---|---|---|---|---|---|---|
| **SH2D4A** | SH2 domain containing 4A | -0.228 | 0.0501 | -0.278 | 0.885 | 0.977 | 0.857 | FALSE | FALSE | FALSE |
| **SH3GL1** | Endophilin-A2 | -0.996 | 0.121 | -1.12 | 0.435 | 0.952 | 0.412 | FALSE | FALSE | FALSE |
| **SHTN1** | Shootin-1 | -2.48 | -1.27 | -1.21 | 0.246 | 0.629 | 0.62 | FALSE | FALSE | FALSE |
| **SKAP2** | Src kinase-associated phosphoprotein 2 | -0.65 | -1.39 | 0.735 | 0.704 | 0.453 | 0.706 | FALSE | FALSE | FALSE |
| **SKP1** | S-phase kinase-associated protein 1 | 0.974 | 0.664 | 0.31 | 0.394 | 0.637 | 0.838 | FALSE | FALSE | FALSE |
| **SLC11A2** | Natural resistance-associated macrophage protein 1 | 0.102 | -2.41 | 2.51 | 0.957 | 0.274 | 0.216 | FALSE | FALSE | FALSE |
| **SLC11A2.1** | Natural resistance-associated macrophage protein 1 | 0.883 | -1.23 | 2.11 | 0.621 | 0.538 | 0.249 | FALSE | FALSE | FALSE |
| **SLC12A2** | Solute carrier family 12 member 2 | -2.52 | -2.39 | -0.127 | 0.0122 | 0.0947 | 0.915 | TRUE | FALSE | FALSE |
| **SLC1A1** | Excitatory amino acid transporter 3 | 0.146 | -0.921 | 1.07 | 0.917 | 0.591 | 0.488 | FALSE | FALSE | FALSE |
| **SLC25A6** | ADP/ATP translocase 3 | -0.217 | 1.19 | -1.41 | 0.885 | 0.393 | 0.279 | FALSE | FALSE | FALSE |
| **SLC28A3** | Sodium/nucleoside cotransporter | -0.889 | -0.256 | -0.633 | 0.36 | 0.851 | 0.558 | FALSE | FALSE | FALSE |
| **SLC2A3** | Solute carrier family 2 facilitated glucose transporter member 3 | -1.26 | -1.41 | 0.149 | 0.434 | 0.438 | 0.937 | FALSE | FALSE | FALSE |

| | | | | | | | | | | |
|---|---|---|---|---|---|---|---|---|---|---|
| **SLC31A1** | Copper transport protein | -0.329 | -1.95 | 1.62 | 0.881 | 0.342 | 0.386 | FALSE | FALSE | FALSE |
| **SLC34A2** | Sodium-dependent phosphate transport protein 2B | -0.51 | -1.71 | 1.21 | 0.623 | 0.22 | 0.258 | FALSE | FALSE | FALSE |
| **SLC3A2** | Solute carrier family 3 member 2 | 0.158 | 1.71 | -1.55 | 0.9 | 0.256 | 0.229 | FALSE | FALSE | FALSE |
| **SLC44A1** | Choline transporter-like protein | -0.736 | -0.0932 | -0.643 | 0.571 | 0.959 | 0.658 | FALSE | FALSE | FALSE |
| **SLC44A2** | Choline transporter-like protein 2 | 0.161 | -0.341 | 0.502 | 0.914 | 0.867 | 0.784 | FALSE | FALSE | FALSE |
| **SLC44A4** | Choline transporter-like protein | 0.39 | -1.24 | 1.63 | 0.782 | 0.403 | 0.249 | FALSE | FALSE | FALSE |
| **SLC4A1** | Anion exchange protein | -0.844 | 0.801 | -1.65 | 0.657 | 0.717 | 0.386 | FALSE | FALSE | FALSE |
| **SLC5A1** | Solute carrier family 5 member 1 | 0.123 | -0.918 | 1.04 | 0.937 | 0.629 | 0.551 | FALSE | FALSE | FALSE |
| **SLC5A6** | Solute carrier family 5 member 6 | -0.323 | -0.134 | -0.189 | 0.756 | 0.931 | 0.875 | FALSE | FALSE | FALSE |
| **SLC9A3R1** | Na(+)/H(+) exchange regulatory cofactor NHE-RF1 | 5.8 | 0.846 | 4.95 | 0.00295 | 0.69 | 0.0177 | TRUE | FALSE | TRUE |
| **SLK** | Non-specific serine/threonine protein kinase | 1.07 | -1.19 | 2.26 | 0.375 | 0.388 | 0.0873 | FALSE | FALSE | FALSE |
| **SMAD2** | Mothers against decapentaplegic homolog | 0.118 | 0.343 | -0.225 | 0.944 | 0.883 | 0.912 | FALSE | FALSE | FALSE |

| | | | | | | | | | | |
|---|---|---|---|---|---|---|---|---|---|---|
| **SMARCA4** | Transcription activator BRG1 | -1.02 | -1.27 | 0.247 | 0.359 | 0.328 | 0.857 | FALSE | FALSE | FALSE |
| **SMARCC2** | SWI/SNF related matrix associated | -1.05 | 1.12 | -2.17 | 0.504 | 0.538 | 0.207 | FALSE | FALSE | FALSE |
| **SMG8** | Nonsense-mediated mRNA decay factor SMG8 | -2.15 | -1.62 | -0.533 | 0.172 | 0.373 | 0.791 | FALSE | FALSE | FALSE |
| **SMPDL3B** | Acid sphingomyelinase-like phosphodiesterase | 1.22 | -0.772 | 1.99 | 0.364 | 0.636 | 0.179 | FALSE | FALSE | FALSE |
| **SMS** | Spermine synthase | -0.543 | 0.576 | -1.12 | 0.682 | 0.708 | 0.403 | FALSE | FALSE | FALSE |
| **SNAP23** | Synaptosomal-associated protein | 0.0476 | -0.397 | 0.445 | 0.975 | 0.852 | 0.821 | FALSE | FALSE | FALSE |
| **SND1** | Staphylococcal nuclease domain-containing protein 1 | -0.285 | 0.0736 | -0.359 | 0.842 | 0.967 | 0.824 | FALSE | FALSE | FALSE |
| **SNRNP200** | Small nuclear ribonucleoprotein U5 subunit 200 | -0.973 | -0.596 | -0.378 | 0.404 | 0.678 | 0.802 | FALSE | FALSE | FALSE |
| **SNRNP70** | U1 small nuclear ribonucleoprotein 70 kDa | -0.49 | -0.12 | -0.37 | 0.662 | 0.939 | 0.782 | FALSE | FALSE | FALSE |
| **SNRPD1** | Small nuclear ribonucleoprotein Sm D1 | -0.44 | 0.468 | -0.908 | 0.723 | 0.754 | 0.473 | FALSE | FALSE | FALSE |
| **SNRPD2** | Small nuclear ribonucleoprotein Sm D2 | 1.94 | 1.11 | 0.831 | 0.0617 | 0.363 | 0.469 | FALSE | FALSE | FALSE |

| | | | | | | | | | | |
|---|---|---|---|---|---|---|---|---|---|---|
| **SNRPD3** | Small nuclear ribonucleoprotein Sm D3 | -0.651 | 0.0417 | -0.692 | 0.503 | 0.974 | 0.511 | FALSE | FALSE | FALSE |
| **SNX18** | Sorting nexin | 0.12 | 0.078 | 0.0418 | 0.916 | 0.962 | 0.977 | FALSE | FALSE | FALSE |
| **SNX9** | Sorting nexin | -0.185 | 0.103 | -0.288 | 0.873 | 0.942 | 0.816 | FALSE | FALSE | FALSE |
| **SOD1** | Superoxide dismutase [Cu-Zn] | 2.09 | 1.36 | 0.731 | 0.0745 | 0.335 | 0.585 | FALSE | FALSE | FALSE |
| **SOX17** | SRY-box transcription factor 17 | -2.7 | -2.17 | -0.526 | 0.0228 | 0.201 | 0.714 | TRUE | FALSE | FALSE |
| **SPEF1** | Sperm flagellar protein 1 | -1.99 | 0.324 | -2.31 | 0.262 | 0.901 | 0.235 | FALSE | FALSE | FALSE |
| **SPON1** | Spondin-1 | 0.328 | 0.58 | -0.252 | 0.774 | 0.66 | 0.855 | FALSE | FALSE | FALSE |
| **SPRED2** | Sprouty related EVH1 domain containing 2 | -0.189 | -0.89 | 0.7 | 0.9 | 0.597 | 0.663 | FALSE | FALSE | FALSE |
| **SPTAN1** | Spectrin alpha non-erythrocytic 1 | 5.52 | 2.22 | 3.3 | 0.00209 | 0.259 | 0.0676 | TRUE | FALSE | FALSE |
| **SPTBN1** | Spectrin beta chain | 5.87 | 2.46 | 3.41 | 0.00626 | 0.31 | 0.131 | TRUE | FALSE | FALSE |
| **SPTBN2** | Spectrin beta chain | 1.97 | 2.64 | -0.677 | 0.295 | 0.259 | 0.772 | FALSE | FALSE | FALSE |
| **SQSTM1** | Sequestosome 1 | 0.766 | 1.28 | -0.51 | 0.755 | 0.652 | 0.857 | FALSE | FALSE | FALSE |
| **SRC** | Tyrosine-protein kinase | 0.51 | -0.889 | 1.4 | 0.639 | 0.446 | 0.222 | FALSE | FALSE | FALSE |
| **SRI** | Sorcin | -3.66 | -1.55 | -2.11 | 0.00412 | 0.271 | 0.101 | TRUE | FALSE | FALSE |
| **SRP68** | Signal recognition particle subunit SRP68 | -0.944 | 0.287 | -1.23 | 0.41 | 0.858 | 0.318 | FALSE | FALSE | FALSE |
| **SRP72** | Signal recognition particle subunit SRP72 | 1.74 | 1.47 | 0.269 | 0.2 | 0.356 | 0.874 | FALSE | FALSE | FALSE |

| | | | | | | | | | | |
|---|---|---|---|---|---|---|---|---|---|---|
| **SRPK1** | SRSF protein kinase 1 | -1.07 | -0.418 | -0.652 | 0.426 | 0.818 | 0.678 | FALSE | FALSE | FALSE |
| **SRPK2** | SRSF protein kinase 2 | 0.908 | 2.21 | -1.3 | 0.527 | 0.236 | 0.385 | FALSE | FALSE | FALSE |
| **SRSF10** | Serine and arginine rich splicing factor 10 | 0.167 | 0.971 | -0.805 | 0.905 | 0.541 | 0.6 | FALSE | FALSE | FALSE |
| **SRSF2** | Serine/arginine-rich splicing factor 2 | -0.923 | -0.783 | -0.14 | 0.354 | 0.487 | 0.903 | FALSE | FALSE | FALSE |
| **SRSF3** | Serine/arginine-rich splicing factor 3 | 2.42 | 1.41 | 1.01 | 0.0263 | 0.284 | 0.386 | TRUE | FALSE | FALSE |
| **SRSF7** | Serine/arginine-rich-splicing factor 7 | -0.222 | -0.228 | 0.00586 | 0.864 | 0.876 | 0.997 | FALSE | FALSE | FALSE |
| **SRSF9** | SFRS9 protein | -1.15 | -0.549 | -0.598 | 0.363 | 0.723 | 0.683 | FALSE | FALSE | FALSE |
| **SSB** | Lupus La protein homolog | -1.31 | -0.834 | -0.48 | 0.249 | 0.538 | 0.73 | FALSE | FALSE | FALSE |
| **ST13** | ST13 Hsp70 interacting protein | -1.03 | -0.663 | -0.372 | 0.381 | 0.646 | 0.807 | FALSE | FALSE | FALSE |
| **ST14** | Suppressor of tumorigenicity 14 protein homolog | -1.54 | -1.08 | -0.455 | 0.262 | 0.5 | 0.796 | FALSE | FALSE | FALSE |
| **STAM2** | Signal transducing adaptor molecule 2 | -0.638 | 0.624 | -1.26 | 0.644 | 0.69 | 0.362 | FALSE | FALSE | FALSE |
| **STAMBP** | STAM binding protein | -1.09 | 0.139 | -1.23 | 0.503 | 0.955 | 0.476 | FALSE | FALSE | FALSE |
| **STAT1** | Signal transducer and activator of transcription | 1.03 | -2.46 | 3.48 | 0.422 | 0.186 | 0.0214 | FALSE | FALSE | TRUE |
| **STAT3** | Signal transducer and activator of transcription | -0.594 | 0.532 | -1.13 | 0.684 | 0.763 | 0.446 | FALSE | FALSE | FALSE |

| | | | | | | | | | | |
|---|---|---|---|---|---|---|---|---|---|---|
| **STAU1** | Staufen double-stranded RNA binding protein 1 | -0.991 | -0.824 | -0.167 | 0.4 | 0.557 | 0.903 | FALSE | FALSE | FALSE |
| **STAU2** | Staufen double-stranded RNA binding protein 2 | -1.13 | -0.842 | -0.289 | 0.257 | 0.465 | 0.825 | FALSE | FALSE | FALSE |
| **STC1** | Stanniocalcin-1 | 1.9 | 1.96 | -0.0553 | 0.166 | 0.257 | 0.975 | FALSE | FALSE | FALSE |
| **STIP1** | Stress-induced-phosphoprotein 1 | -0.775 | 1.04 | -1.82 | 0.671 | 0.618 | 0.327 | FALSE | FALSE | FALSE |
| **STK10** | Non-specific serine/threonine protein kinase | -2.48 | -2.64 | 0.152 | 0.171 | 0.256 | 0.943 | FALSE | FALSE | FALSE |
| **STK24** | STK24 protein | 1.71 | -0.303 | 2.02 | 0.153 | 0.859 | 0.137 | FALSE | FALSE | FALSE |
| **STK26** | Serine/threonine kinase 26 | -0.165 | -2.15 | 1.98 | 0.94 | 0.388 | 0.386 | FALSE | FALSE | FALSE |
| **STOM** | STOM protein | -0.355 | -1.58 | 1.22 | 0.822 | 0.335 | 0.411 | FALSE | FALSE | FALSE |
| **STRAP** | Serine-threonine kinase receptor-associated protein | -0.447 | 0.458 | -0.905 | 0.679 | 0.716 | 0.404 | FALSE | FALSE | FALSE |
| **STRBP** | Spermatid perinuclear RNA-binding protein | 0.177 | 0.671 | -0.494 | 0.892 | 0.625 | 0.715 | FALSE | FALSE | FALSE |
| **STRN** | Striatin | -1.31 | -0.34 | -0.972 | 0.4 | 0.874 | 0.575 | FALSE | FALSE | FALSE |
| **STUM** | MGC151592 protein | -0.515 | -0.54 | 0.0258 | 0.656 | 0.688 | 0.985 | FALSE | FALSE | FALSE |
| **STX11** | Syntaxin 11 | 0.286 | 0.895 | -0.609 | 0.892 | 0.678 | 0.783 | FALSE | FALSE | FALSE |
| **STX3** | Syntaxin 3 | -0.0484 | -0.668 | 0.62 | 0.964 | 0.583 | 0.585 | FALSE | FALSE | FALSE |
| **STX7** | Syntaxin-7 | 0.0799 | -0.0228 | 0.103 | 0.943 | 0.99 | 0.937 | FALSE | FALSE | FALSE |
| **STXBP1** | Syntaxin-binding protein 1 | -0.11 | -0.2 | 0.0893 | 0.943 | 0.933 | 0.966 | FALSE | FALSE | FALSE |
| **STXBP2** | Syntaxin binding protein 2 | -0.559 | -1.21 | 0.649 | 0.727 | 0.486 | 0.72 | FALSE | FALSE | FALSE |

| | | | | | | | | | | |
|---|---|---|---|---|---|---|---|---|---|---|
| **STXBP3** | Syntaxin binding protein 3 | -0.287 | -0.0409 | -0.246 | 0.809 | 0.978 | 0.856 | FALSE | FALSE | FALSE |
| **SULT1A1** | Sulfotransferase 1A1 | 2.91 | 0.243 | 2.67 | 0.0704 | 0.923 | 0.144 | FALSE | FALSE | FALSE |
| **SULT1C4** | Sulfotransferase | -0.7 | -0.353 | -0.347 | 0.51 | 0.8 | 0.796 | FALSE | FALSE | FALSE |
| **SUSD2** | Sushi domain containing 2 | 0.375 | -1.02 | 1.39 | 0.737 | 0.393 | 0.227 | FALSE | FALSE | FALSE |
| **SYNCRIP** | Synaptotagmin binding cytoplasmic RNA interacting protein | 6.48 | 3.82 | 2.66 | 0.000938 | 0.113 | 0.146 | TRUE | FALSE | FALSE |
| **SYPL1** | Synaptophysin like 1 | -0.498 | 0.123 | -0.621 | 0.709 | 0.952 | 0.677 | FALSE | FALSE | FALSE |
| **SYTL1** | Synaptotagmin like 1 | -0.225 | 1.29 | -1.52 | 0.884 | 0.363 | 0.254 | FALSE | FALSE | FALSE |
| **SYTL4** | Synaptotagmin like 4 | -0.408 | 0.274 | -0.683 | 0.717 | 0.857 | 0.57 | FALSE | FALSE | FALSE |
| **TACSTD2** | Tumor associated calcium signal transducer 2 | 0.387 | 0.775 | -0.388 | 0.761 | 0.599 | 0.803 | FALSE | FALSE | FALSE |
| **TAGLN2** | Transgelin-2 | 0.68 | -0.936 | 1.62 | 0.709 | 0.66 | 0.384 | FALSE | FALSE | FALSE |
| **TALDO1** | Transaldolase | -5.75 | -3.9 | -1.85 | 0.00617 | 0.186 | 0.381 | TRUE | FALSE | FALSE |
| **TAOK3** | TAO kinase 3 | 0.446 | 0.442 | 0.00472 | 0.71 | 0.763 | 0.997 | FALSE | FALSE | FALSE |
| **TAP1** | TAP1 protein | -1.54 | -1.28 | -0.254 | 0.243 | 0.393 | 0.875 | FALSE | FALSE | FALSE |
| **TAPBP** | TAP binding protein | 0.158 | 0.914 | -0.756 | 0.898 | 0.477 | 0.548 | FALSE | FALSE | FALSE |
| **TARDBP** | TAR DNA-binding protein 43 | -0.442 | 1.18 | -1.63 | 0.709 | 0.363 | 0.194 | FALSE | FALSE | FALSE |
| **TARS1** | Threonine-tRNA ligase 1 cytoplasmic | -1.5 | -1.16 | -0.339 | 0.206 | 0.393 | 0.825 | FALSE | FALSE | FALSE |
| **TBC1D10A** | TBC1 domain family member 10A | 0.158 | 0.379 | -0.22 | 0.89 | 0.749 | 0.853 | FALSE | FALSE | FALSE |

| | | | | | | | | | | |
|---|---|---|---|---|---|---|---|---|---|---|
| **TBL1XR1** | TBL1X receptor 1 | -0.612 | 0.0739 | -0.686 | 0.658 | 0.967 | 0.654 | FALSE | FALSE | FALSE |
| **TCAF2** | TRPM8 channel-associated factor 2 | 0.83 | 0.436 | 0.394 | 0.508 | 0.788 | 0.804 | FALSE | FALSE | FALSE |
| **TCEB2** | Transcription elongation factor B (SIII) polypeptide 2 | -0.657 | -0.0784 | -0.579 | 0.568 | 0.96 | 0.654 | FALSE | FALSE | FALSE |
| **TCN1** | Transcobalamin 1 | -7.82 | -4.4 | -3.42 | 0.000495 | 0.0943 | 0.0873 | TRUE | FALSE | FALSE |
| **TCP1** | T-complex protein 1 subunit alpha | 0.868 | 0.301 | 0.566 | 0.452 | 0.854 | 0.677 | FALSE | FALSE | FALSE |
| **TDGF1** | Growth factor | -2.63 | -3.01 | 0.382 | 0.145 | 0.232 | 0.862 | FALSE | FALSE | FALSE |
| **TEP1** | Telomerase associated protein 1 | -1.16 | -0.623 | -0.537 | 0.376 | 0.69 | 0.73 | FALSE | FALSE | FALSE |
| **TES** | Testin | 2.01 | 0.868 | 1.14 | 0.0656 | 0.501 | 0.336 | FALSE | FALSE | FALSE |
| **TFF3** | Trefoil factor 3 | 0.644 | 0.146 | 0.498 | 0.606 | 0.936 | 0.726 | FALSE | FALSE | FALSE |
| **TFG** | TFG protein | 0.457 | 0.0728 | 0.384 | 0.698 | 0.964 | 0.788 | FALSE | FALSE | FALSE |
| **TFPI2** | Tissue factor pathway inhibitor 2 | -1.31 | 0.0146 | -1.33 | 0.609 | 0.996 | 0.636 | FALSE | FALSE | FALSE |
| **TGFBI** | Transforming growth factor-beta-induced protein ig-h3 | -0.174 | 0.0747 | -0.249 | 0.89 | 0.959 | 0.853 | FALSE | FALSE | FALSE |
| **TGM2** | Protein-glutamine gamma-glutamyltransferase 2 | -0.153 | 0.171 | -0.323 | 0.907 | 0.933 | 0.846 | FALSE | FALSE | FALSE |
| **THY1** | Thy-1 cell surface antigen | -1.68 | -2.02 | 0.341 | 0.231 | 0.257 | 0.85 | FALSE | FALSE | FALSE |
| **TIMP2** | Metalloproteinase inhibitor 2 | 0.576 | 0.134 | 0.442 | 0.607 | 0.935 | 0.73 | FALSE | FALSE | FALSE |

| | | | | | | | | | | |
|---|---|---|---|---|---|---|---|---|---|---|
| **TKFC** | Triokinase/FMN cyclase | 0.719 | 0.629 | 0.0898 | 0.554 | 0.66 | 0.951 | FALSE | FALSE | FALSE |
| **TKT** | TKT protein | -1.53 | -0.624 | -0.908 | 0.222 | 0.688 | 0.511 | FALSE | FALSE | FALSE |
| **TLE3** | TLE family member 3 transcriptional corepressor | -0.932 | -0.24 | -0.693 | 0.498 | 0.903 | 0.661 | FALSE | FALSE | FALSE |
| **TLN1** | Talin 1 | -1.16 | -1.69 | 0.534 | 0.641 | 0.538 | 0.856 | FALSE | FALSE | FALSE |
| **TLR2** | Toll-like receptor 2 | 0.0227 | -0.641 | 0.664 | 0.99 | 0.723 | 0.703 | FALSE | FALSE | FALSE |
| **TMC5** | Transmembrane channel-like protein | -1.05 | 0.0904 | -1.14 | 0.306 | 0.955 | 0.303 | FALSE | FALSE | FALSE |
| **TMED10** | Transmembrane emp24 domain-containing protein 10 | 0.205 | -0.0824 | 0.288 | 0.845 | 0.952 | 0.803 | FALSE | FALSE | FALSE |
| **TMED2** | RNP24 | -0.618 | 0.429 | -1.05 | 0.609 | 0.768 | 0.39 | FALSE | FALSE | FALSE |
| **TMED4** | Transmembrane p24 trafficking protein 4 | -0.32 | 0.932 | -1.25 | 0.809 | 0.502 | 0.329 | FALSE | FALSE | FALSE |
| **TMED7** | TMED7 protein | -0.0727 | 1.27 | -1.34 | 0.95 | 0.339 | 0.274 | FALSE | FALSE | FALSE |
| **TMED9** | Transmembrane emp24 domain-containing protein 9 | 1.49 | 1.34 | 0.151 | 0.203 | 0.335 | 0.915 | FALSE | FALSE | FALSE |
| **TMEM30A** | Cell cycle control protein | -0.0321 | 0.467 | -0.499 | 0.979 | 0.756 | 0.726 | FALSE | FALSE | FALSE |
| **TMEM67** | Transmembrane protein 67 | -2.78 | -1.54 | -1.24 | 0.209 | 0.564 | 0.625 | FALSE | FALSE | FALSE |
| **TMSB4** | Thymosin beta-4 | -1.33 | 0.981 | -2.31 | 0.4 | 0.606 | 0.185 | FALSE | FALSE | FALSE |
| **TNC** | Tenascin C | -2.84 | -3.13 | 0.286 | 0.249 | 0.291 | 0.921 | FALSE | FALSE | FALSE |
| **TNFAIP6** | TNF alpha induced protein 6 | -1.63 | -0.451 | -1.18 | 0.172 | 0.767 | 0.355 | FALSE | FALSE | FALSE |

| | | | | | | | | | | |
|---|---|---|---|---|---|---|---|---|---|---|
| **TNFAIP8** | Tumor necrosis factor alpha-induced protein 8 | 0.0586 | 0.219 | -0.16 | 0.97 | 0.931 | 0.93 | FALSE | FALSE | FALSE |
| **TNKS1BP1** | Tankyrase 1 binding protein 1 | -1.12 | -1.36 | 0.247 | 0.302 | 0.289 | 0.856 | FALSE | FALSE | FALSE |
| **TNPO1** | Transportin-1 | -0.925 | -1.1 | 0.176 | 0.444 | 0.419 | 0.901 | FALSE | FALSE | FALSE |
| **TOE1** | Target of EGR1 protein 1 | -1.42 | -1.2 | -0.219 | 0.149 | 0.313 | 0.857 | FALSE | FALSE | FALSE |
| **TOLLIP** | Toll-interacting protein | 0.176 | -0.443 | 0.619 | 0.895 | 0.768 | 0.654 | FALSE | FALSE | FALSE |
| **TOM1** | Target of myb1 membrane trafficking protein | -0.0519 | -1.31 | 1.26 | 0.959 | 0.268 | 0.232 | FALSE | FALSE | FALSE |
| **TOM1L1** | Target of myb1 like 1 membrane trafficking protein | -1.09 | -0.365 | -0.723 | 0.343 | 0.807 | 0.569 | FALSE | FALSE | FALSE |
| **TOP1** | DNA topoisomerase I | 2.73 | 2.24 | 0.485 | 0.177 | 0.344 | 0.853 | FALSE | FALSE | FALSE |
| **TOP2B** | DNA topoisomerase 2 | -0.759 | -0.0109 | -0.748 | 0.504 | 0.995 | 0.555 | FALSE | FALSE | FALSE |
| **TP53BP1** | Tumor protein p53 binding protein 1 | -1.24 | -1.49 | 0.247 | 0.257 | 0.268 | 0.857 | FALSE | FALSE | FALSE |
| **TPD52** | Uncharacterized protein | -0.96 | 2.2 | -3.16 | 0.679 | 0.387 | 0.194 | FALSE | FALSE | FALSE |
| **TPI1** | Triosephosphate isomerase | 1.51 | -0.641 | 2.15 | 0.231 | 0.68 | 0.128 | FALSE | FALSE | FALSE |
| **TPMT** | Thiopurine S-methyltransferase | -0.453 | 0.13 | -0.582 | 0.679 | 0.935 | 0.62 | FALSE | FALSE | FALSE |
| **TPP1** | Tripeptidyl-peptidase 1 | -0.954 | 0.0546 | -1.01 | 0.646 | 0.985 | 0.658 | FALSE | FALSE | FALSE |

| | | | | | | | | | | |
|---|---|---|---|---|---|---|---|---|---|---|
| **TPP2** | Tripeptidyl-peptidase 2 | -1.52 | 1.05 | -2.57 | 0.474 | 0.688 | 0.258 | FALSE | FALSE | FALSE |
| **TPPP3** | Tubulin polymerization-promoting protein family member 3 | 2.2 | 2.17 | 0.0273 | 0.0626 | 0.206 | 0.985 | FALSE | FALSE | FALSE |
| **TRA2B** | Transformer-2 protein homolog beta | -1.02 | -0.756 | -0.266 | 0.313 | 0.519 | 0.841 | FALSE | FALSE | FALSE |
| **TRIM25** | Tripartite motif containing 25 | 3.43 | 2.16 | 1.27 | 0.0844 | 0.361 | 0.575 | FALSE | FALSE | FALSE |
| **TRIM28** | Tripartite motif containing 28 | 0.954 | 1.09 | -0.135 | 0.423 | 0.417 | 0.921 | FALSE | FALSE | FALSE |
| **TRIOBP** | TRIO and F-actin binding protein | 0.0641 | 1.05 | -0.985 | 0.961 | 0.469 | 0.471 | FALSE | FALSE | FALSE |
| **TRIP12** | E3 ubiquitin-protein ligase | 0.478 | -0.483 | 0.961 | 0.755 | 0.802 | 0.558 | FALSE | FALSE | FALSE |
| **TSG101** | TSG101 protein | 0.473 | -0.0357 | 0.509 | 0.662 | 0.978 | 0.668 | FALSE | FALSE | FALSE |
| **TSPAN1** | Tetraspanin-1 | 1.94 | 0.465 | 1.48 | 0.0505 | 0.703 | 0.185 | FALSE | FALSE | FALSE |
| **TTC37** | Tetratricopeptide repeat domain 37 | -0.316 | -0.975 | 0.659 | 0.84 | 0.538 | 0.677 | FALSE | FALSE | FALSE |
| **TTR** | Transthyretin | -1.81 | -2.15 | 0.339 | 0.258 | 0.271 | 0.862 | FALSE | FALSE | FALSE |
| **TTYH3** | Protein tweety homolog | -0.37 | -0.414 | 0.0442 | 0.722 | 0.737 | 0.975 | FALSE | FALSE | FALSE |
| **TUBA1B** | Tubulin alpha-1B chain | 1.11 | -1.03 | 2.14 | 0.615 | 0.69 | 0.342 | FALSE | FALSE | FALSE |
| **TUBA1C** | Tubulin alpha-1C chain | -1.06 | 0.195 | -1.25 | 0.41 | 0.919 | 0.357 | FALSE | FALSE | FALSE |
| **TUBA1D** | Tubulin alpha chain | 3.59 | 0.773 | 2.82 | 0.0039 | 0.565 | 0.0312 | TRUE | FALSE | TRUE |
| **TUBA4A** | Tubulin alpha-4A chain | -0.298 | -0.421 | 0.123 | 0.789 | 0.749 | 0.919 | FALSE | FALSE | FALSE |

| | | | | | | | | | | |
|---|---|---|---|---|---|---|---|---|---|---|
| **TUBB2B** | Tubulin beta-2B chain | 3.45 | 1.59 | 1.86 | 0.0181 | 0.349 | 0.239 | TRUE | FALSE | FALSE |
| **TUBB4A** | Tubulin beta-4A chain | 0.0941 | -0.244 | 0.338 | 0.94 | 0.891 | 0.838 | FALSE | FALSE | FALSE |
| **TUBB4B** | Tubulin beta-4B chain | 4.08 | 1.21 | 2.86 | 0.000605 | 0.291 | 0.0142 | TRUE | FALSE | TRUE |
| **TUBB5** | Tubulin beta-5 chain | 2.51 | 0.394 | 2.11 | 0.0443 | 0.816 | 0.132 | TRUE | FALSE | FALSE |
| **TUBB6** | Tubulin beta-6 chain | -0.723 | 0.666 | -1.39 | 0.489 | 0.597 | 0.227 | FALSE | FALSE | FALSE |
| **TWF1** | Twinfilin-1 | -1.46 | -0.786 | -0.674 | 0.139 | 0.496 | 0.549 | FALSE | FALSE | FALSE |
| **TWF2** | TWF2 protein | -0.0603 | -1.36 | 1.3 | 0.97 | 0.416 | 0.407 | FALSE | FALSE | FALSE |
| **TXN** | Thioredoxin | 0.541 | -0.307 | 0.848 | 0.608 | 0.82 | 0.423 | FALSE | FALSE | FALSE |
| **TXNDC17** | Thioredoxin domain-containing protein 17 | -0.7 | 0.0178 | -0.718 | 0.612 | 0.993 | 0.636 | FALSE | FALSE | FALSE |
| **TXNDC5** | Thioredoxin domain containing 5 | -1.97 | -1.96 | -0.0127 | 0.036 | 0.16 | 0.989 | TRUE | FALSE | FALSE |
| **TXNL1** | Thioredoxin like 1 | -1.12 | -1.14 | 0.0201 | 0.379 | 0.424 | 0.988 | FALSE | FALSE | FALSE |
| **U2AF1** | Splicing factor U2AF 35 kDa subunit | 0.792 | 1.75 | -0.963 | 0.457 | 0.232 | 0.395 | FALSE | FALSE | FALSE |
| **U2AF2** | Splicing factor U2AF subunit | -0.719 | 0.274 | -0.993 | 0.513 | 0.858 | 0.39 | FALSE | FALSE | FALSE |
| **UACA** | Uveal autoantigen with coiled-coil domains and ankyrin repeats protein | 1.47 | 1.75 | -0.277 | 0.381 | 0.363 | 0.893 | FALSE | FALSE | FALSE |

| | | | | | | | | | | |
|---|---|---|---|---|---|---|---|---|---|---|
| **UBA1** | Ubiquitin-like modifier-activating enzyme 1 | -0.859 | -1.8 | 0.943 | 0.508 | 0.259 | 0.497 | FALSE | FALSE | FALSE |
| **UBA6** | Ubiquitin like modifier activating enzyme 6 | -0.722 | 0.872 | -1.59 | 0.677 | 0.662 | 0.359 | FALSE | FALSE | FALSE |
| **UBE2D2** | Ubiquitin-conjugating enzyme E2 D2 | -1.4 | -1.58 | 0.18 | 0.218 | 0.261 | 0.898 | FALSE | FALSE | FALSE |
| **UBE2M** | NEDD8-conjugating enzyme Ubc12 | 0.852 | 1.32 | -0.466 | 0.463 | 0.335 | 0.742 | FALSE | FALSE | FALSE |
| **UBE2N** | Ubiquitin-conjugating enzyme E2 N | 0.395 | -0.291 | 0.686 | 0.751 | 0.859 | 0.608 | FALSE | FALSE | FALSE |
| **UBE2V1** | Ubiquitin-conjugating enzyme E2 variant 1 | -0.336 | 0.555 | -0.891 | 0.783 | 0.69 | 0.469 | FALSE | FALSE | FALSE |
| **UBE2Z** | Ubiquitin conjugating enzyme E2 Z | -0.298 | 0.978 | -1.28 | 0.823 | 0.467 | 0.315 | FALSE | FALSE | FALSE |
| **UBR4** | Ubiquitin protein ligase E3 component n-recognin 4 | 2.45 | 1.71 | 0.735 | 0.314 | 0.552 | 0.816 | FALSE | FALSE | FALSE |
| **UCHL3** | Ubiquitin carboxyl-terminal hydrolase isozyme L3 | 0.949 | 1.96 | -1.01 | 0.549 | 0.285 | 0.558 | FALSE | FALSE | FALSE |
| **UGP2** | UTP--glucose-1-phosphate uridylyltransferase | -2.64 | -0.787 | -1.85 | 0.249 | 0.799 | 0.461 | FALSE | FALSE | FALSE |

| | | | | | | | | | | |
|---|---|---|---|---|---|---|---|---|---|---|
| **UGT1A1** | UDP-glucuronosyltransferase | 0.00768 | -0.431 | 0.439 | 0.994 | 0.749 | 0.73 | FALSE | FALSE | FALSE |
| **UMPS** | Uridine 5-monophosphate synthase | 0.858 | 1.75 | -0.888 | 0.436 | 0.236 | 0.457 | FALSE | FALSE | FALSE |
| **UNC13B** | Unc-13 homolog B | -1.45 | 1.04 | -2.49 | 0.509 | 0.69 | 0.282 | FALSE | FALSE | FALSE |
| **UNC5CL** | Unc-5 family C-terminal like | -0.493 | 1.13 | -1.62 | 0.684 | 0.393 | 0.204 | FALSE | FALSE | FALSE |
| **UPF1** | UPF1 RNA helicase and ATPase | -0.0634 | 0.875 | -0.939 | 0.97 | 0.646 | 0.592 | FALSE | FALSE | FALSE |
| **UPK1B** | Uroplakin-1b | -3.5 | -2.52 | -0.977 | 0.114 | 0.339 | 0.72 | FALSE | FALSE | FALSE |
| **UPK3BL1** | Uroplakin-3b-like protein 1 | 4.3 | -0.755 | 5.06 | 0.00976 | 0.7 | 0.0115 | TRUE | FALSE | TRUE |
| **UPP1** | Uridine phosphorylase | -3 | -1.13 | -1.87 | 0.12 | 0.639 | 0.372 | FALSE | FALSE | FALSE |
| **USO1** | General vesicular transport factor p115 | 0.629 | 0.643 | -0.0137 | 0.528 | 0.584 | 0.989 | FALSE | FALSE | FALSE |
| **USP14** | Ubiquitin carboxyl-terminal hydrolase 14 | -0.789 | 0.87 | -1.66 | 0.657 | 0.67 | 0.351 | FALSE | FALSE | FALSE |
| **USP16** | Ubiquitin carboxyl-terminal hydrolase 16 | -2.33 | -1.36 | -0.973 | 0.108 | 0.417 | 0.559 | FALSE | FALSE | FALSE |
| **USP5** | Ubiquitin carboxyl-terminal hydrolase | -0.348 | -0.416 | 0.0684 | 0.738 | 0.734 | 0.957 | FALSE | FALSE | FALSE |
| **USP9X** | Ubiquitinyl hydrolase 1 | -0.684 | -0.122 | -0.562 | 0.498 | 0.936 | 0.62 | FALSE | FALSE | FALSE |

| | | | | | | | | | | |
|---|---|---|---|---|---|---|---|---|---|---|
| **VAMP7** | Vesicle-associated membrane protein 7 | -1.11 | -1.36 | 0.247 | 0.3 | 0.285 | 0.856 | FALSE | FALSE | FALSE |
| **VAMP8** | Vesicle-associated membrane protein 8 | -1.08 | -0.174 | -0.909 | 0.51 | 0.944 | 0.625 | FALSE | FALSE | FALSE |
| **VARS1** | Valine--tRNA ligase | -0.858 | 0.00985 | -0.868 | 0.439 | 0.995 | 0.471 | FALSE | FALSE | FALSE |
| **VASP** | Vasodilator-stimulated phosphoprotein | 0.809 | 0.114 | 0.695 | 0.457 | 0.946 | 0.57 | FALSE | FALSE | FALSE |
| **VAT1** | Vesicle amine transport 1 | -1.12 | -1.21 | 0.091 | 0.371 | 0.393 | 0.954 | FALSE | FALSE | FALSE |
| **VAT1L** | Vesicle amine transport 1 like | -2.38 | -1.86 | -0.522 | 0.221 | 0.4 | 0.839 | FALSE | FALSE | FALSE |
| **VCAN** | Versican core protein | 5.74 | 0.234 | 5.51 | 0.0084 | 0.94 | 0.0254 | TRUE | FALSE | TRUE |
| **VCL** | Vinculin | -2.57 | -1.26 | -1.31 | 0.12 | 0.519 | 0.471 | FALSE | FALSE | FALSE |
| **VCP** | Transitional endoplasmic reticulum ATPase | 1.73 | -0.418 | 2.15 | 0.0935 | 0.756 | 0.0732 | FALSE | FALSE | FALSE |
| **VDAC1** | Voltage-dependent anion-selective channel protein 1 | -1.05 | 1.44 | -2.49 | 0.528 | 0.431 | 0.17 | FALSE | FALSE | FALSE |
| **VDAC2** | Voltage-dependent anion-selective channel protein 2 | 0.833 | 1.29 | -0.453 | 0.647 | 0.519 | 0.841 | FALSE | FALSE | FALSE |
| **VIM** | Vimentin | -2.57 | -3.01 | 0.437 | 0.152 | 0.232 | 0.851 | FALSE | FALSE | FALSE |
| **VNN2** | Vanin 2 | -1.21 | -2.03 | 0.82 | 0.247 | 0.186 | 0.471 | FALSE | FALSE | FALSE |
| **VPS13C** | Vacuolar protein sorting 13 homolog C | 0.0307 | -0.0484 | 0.0791 | 0.984 | 0.978 | 0.967 | FALSE | FALSE | FALSE |

| | | | | | | | | | | |
|---|---|---|---|---|---|---|---|---|---|---|
| **VPS16** | Vacuolar protein sorting-associated protein 16 homolog | -0.499 | 1.33 | -1.83 | 0.738 | 0.4 | 0.229 | FALSE | FALSE | FALSE |
| **VPS26A** | Vacuolar protein sorting-associated protein 26A | 0.00883 | 0.271 | -0.262 | 0.993 | 0.838 | 0.835 | FALSE | FALSE | FALSE |
| **VPS28** | Vacuolar protein sorting-associated protein 28 homolog | -0.943 | 0.569 | -1.51 | 0.56 | 0.777 | 0.36 | FALSE | FALSE | FALSE |
| **VPS29** | Vacuolar protein sorting-associated protein 29 | -1.44 | -1.09 | -0.355 | 0.222 | 0.417 | 0.818 | FALSE | FALSE | FALSE |
| **VPS35** | Vacuolar protein sorting-associated protein 35 | -0.229 | -1.85 | 1.62 | 0.895 | 0.326 | 0.336 | FALSE | FALSE | FALSE |
| **VPS4B** | Vesicle-fusing ATPase | 1.41 | -0.559 | 1.96 | 0.229 | 0.69 | 0.132 | FALSE | FALSE | FALSE |
| **VSIR** | V-set immunoregulatory receptor | 0.253 | 2.41 | -2.16 | 0.886 | 0.232 | 0.179 | FALSE | FALSE | FALSE |
| **VTA1** | Vacuolar protein sorting-associated protein VTA1 homolog | 0.158 | 2.14 | -1.99 | 0.907 | 0.236 | 0.181 | FALSE | FALSE | FALSE |
| **VWA7** | von Willebrand factor A domain-containing protein 7 | 2.98 | 3.02 | -0.0462 | 0.275 | 0.343 | 0.988 | FALSE | FALSE | FALSE |
| **VWF** | von Willebrand factor | 0.569 | 0.453 | 0.115 | 0.641 | 0.756 | 0.93 | FALSE | FALSE | FALSE |
| **WARS1** | Tryptophan-tRNA ligase cytoplasmic | 1.4 | -3.45 | 4.86 | 0.449 | 0.197 | 0.0254 | FALSE | FALSE | TRUE |

| | | | | | | | | | | |
|---|---|---|---|---|---|---|---|---|---|---|
| **WASF2** | Actin-binding protein WASF2 | 0.686 | 0.587 | 0.0995 | 0.557 | 0.67 | 0.941 | FALSE | FALSE | FALSE |
| **WDR1** | WD repeat-containing protein 1 | -1.82 | -1.94 | 0.116 | 0.198 | 0.265 | 0.946 | FALSE | FALSE | FALSE |
| **WDR18** | WD repeat-containing protein 18 | -0.805 | 0.579 | -1.38 | 0.569 | 0.736 | 0.342 | FALSE | FALSE | FALSE |
| **WDR37** | WD repeat domain 37 | -0.563 | 0.342 | -0.905 | 0.609 | 0.804 | 0.416 | FALSE | FALSE | FALSE |
| **WDR61** | WD repeat-containing protein 61 | -1.67 | -0.541 | -1.13 | 0.377 | 0.835 | 0.592 | FALSE | FALSE | FALSE |
| **WDR77** | Methylosome protein 50 | -1.5 | -1.74 | 0.247 | 0.107 | 0.197 | 0.841 | FALSE | FALSE | FALSE |
| **WFDC2** | WAP four-disulfide core domain 2 | 0.23 | -1.4 | 1.63 | 0.886 | 0.363 | 0.253 | FALSE | FALSE | FALSE |
| **WNT5A** | Protein Wnt | -0.747 | 0.775 | -1.52 | 0.439 | 0.487 | 0.159 | FALSE | FALSE | FALSE |
| **XPNPEP1** | Xaa-Pro aminopeptidase 1 | 0.502 | 1.83 | -1.33 | 0.702 | 0.256 | 0.326 | FALSE | FALSE | FALSE |
| **XPO1** | Exportin 1 | -0.232 | -0.917 | 0.685 | 0.905 | 0.684 | 0.759 | FALSE | FALSE | FALSE |
| **YARS1** | Tyrosine--tRNA ligase | -1.02 | 0.459 | -1.48 | 0.561 | 0.848 | 0.412 | FALSE | FALSE | FALSE |
| **YBX1** | Y-box-binding protein 1 | 4.03 | 1.89 | 2.14 | 0.00399 | 0.253 | 0.132 | TRUE | FALSE | FALSE |
| **YBX3** | Y-box binding protein 3 | 0.592 | 0.634 | -0.0416 | 0.539 | 0.571 | 0.975 | FALSE | FALSE | FALSE |
| **YES1** | Tyrosine-protein kinase | 1.94 | -1.29 | 3.22 | 0.235 | 0.494 | 0.0797 | FALSE | FALSE | FALSE |
| **YKT6** | Synaptobrevin homolog YKT6 | -0.107 | -0.291 | 0.184 | 0.927 | 0.862 | 0.9 | FALSE | FALSE | FALSE |

| | | | | | | | | | | |
|---|---|---|---|---|---|---|---|---|---|---|
| **YWHAB** | 14-3-3 protein beta/alpha | -1.98 | -1.82 | -0.16 | 0.132 | 0.265 | 0.919 | FALSE | FALSE | FALSE |
| **YWHAE** | 14-3-3 protein epsilon | -0.145 | -1.21 | 1.06 | 0.917 | 0.446 | 0.482 | FALSE | FALSE | FALSE |
| **YWHAG** | 14-3-3 protein gamma | -0.516 | -1.25 | 0.738 | 0.708 | 0.4 | 0.62 | FALSE | FALSE | FALSE |
| **YWHAH** | 14-3-3 protein eta | -0.705 | -0.0775 | -0.627 | 0.676 | 0.969 | 0.744 | FALSE | FALSE | FALSE |
| **YWHAQ** | 14-3-3 protein theta | -0.774 | -0.825 | 0.0511 | 0.546 | 0.578 | 0.975 | FALSE | FALSE | FALSE |
| **YWHAZ** | 14-3-3 protein zeta/delta | -0.54 | -0.987 | 0.447 | 0.681 | 0.491 | 0.773 | FALSE | FALSE | FALSE |
| **ZC3HAV1** | Zinc finger CCCH-type containing antiviral 1 | -0.471 | 0.262 | -0.733 | 0.688 | 0.866 | 0.559 | FALSE | FALSE | FALSE |
| **ZFR** | Zinc finger RNA binding protein | -1.31 | -1.55 | 0.247 | 0.243 | 0.261 | 0.857 | FALSE | FALSE | FALSE |
| **ZNFX1** | Zinc finger NFX1-type containing 1 | -1.19 | -0.646 | -0.541 | 0.382 | 0.69 | 0.743 | FALSE | FALSE | FALSE |

H: healthy cows; SE: cows with subclinical endometritis; CLE: cows with clinical endometritis
