# Supplementary material for: Investigation of Uterine Fluid Extracellular Vesicles’ Proteomic Profiles Provides Novel Diagnostic Biomarkers of Bovine Endometritis
Source: Biomolecules. 2024 May 25;14(6):626. doi: 10.3390/biom14060626 (PMC11202259; doi:10.3390/biom14060626)
Supplement: Supplementary file 1 [file biomolecules-14-00626-s001.zip › biomolecules-3005193-supplementary/Additional file 5.pdf]

Originals

HTRA1

Exposure time 1 min. Samples are in order:
H1, H2, H3, SE1, SE2, SE3, CLE1, CLE2, CL3, RL95, Ishikawa

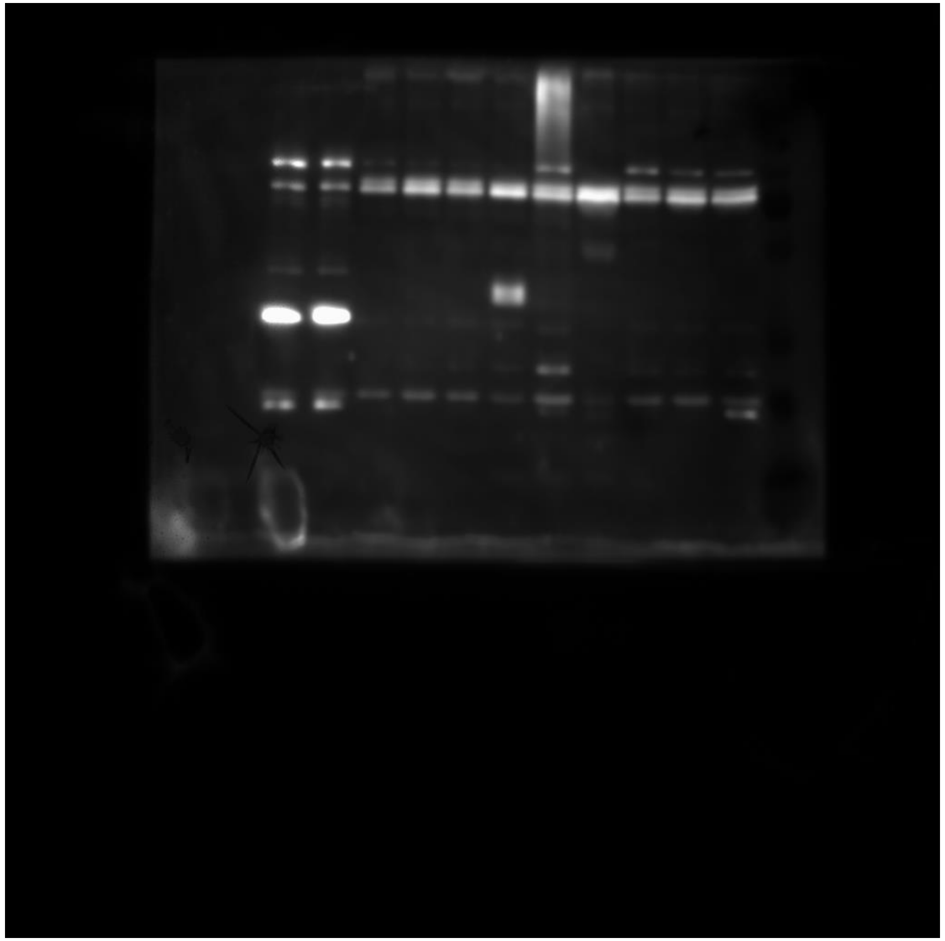

H = healthy
SE = subclinical endometritis
CLE = clinical endometritis

β-actin

Exposure time 30s. Samples are in order:
H1, H2, H3, SE1, SE2, SE3, CLE1, CLE2, CL3, RL95, Ishikawa

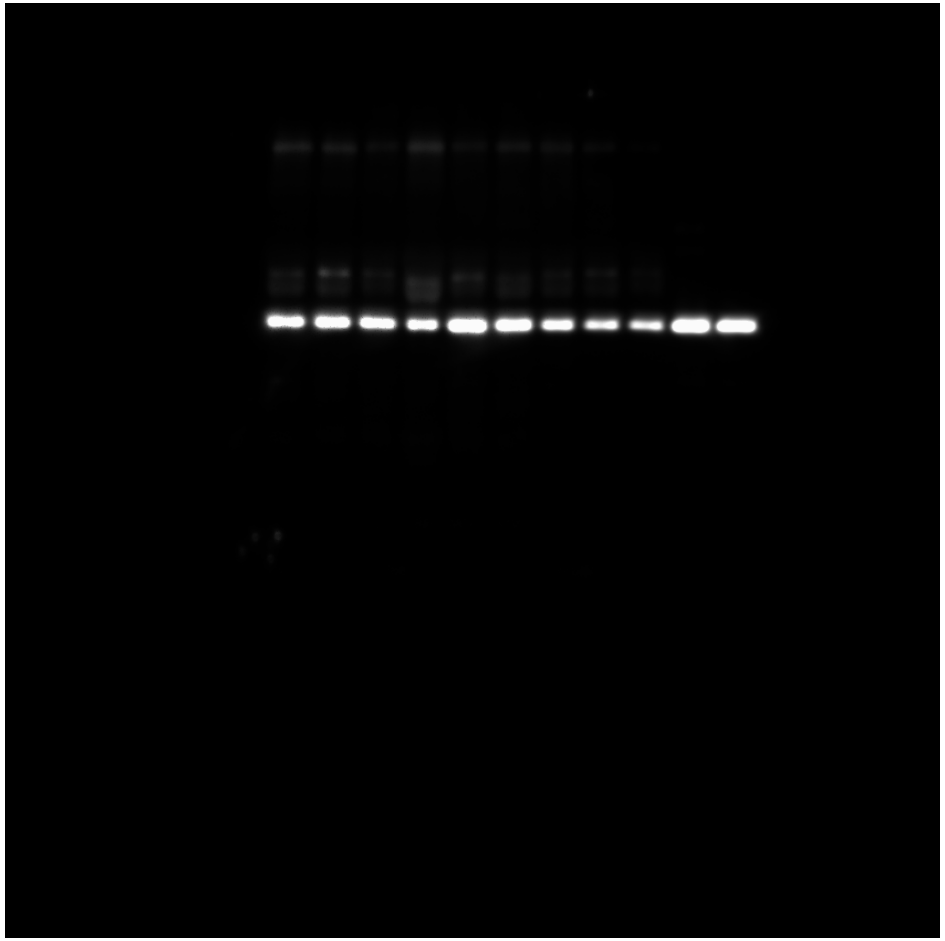

Inverted images

HTRA1

Exposure time 1 min. Samples are in order:
H1, H2, H3, SE1, SE2, SE3, CLE1, CLE2, CL3, RL95, Ishikawa

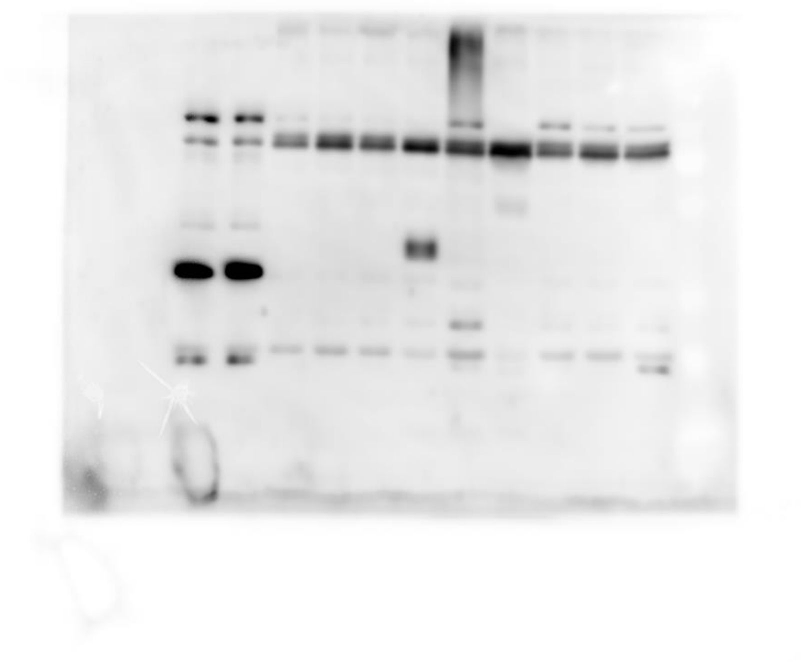

β-actin

Exposure time 30s. Samples are in order:
H1, H2, H3, SE1, SE2, SE3, CLE1, CLE2, CL3, RL95, Ishikawa

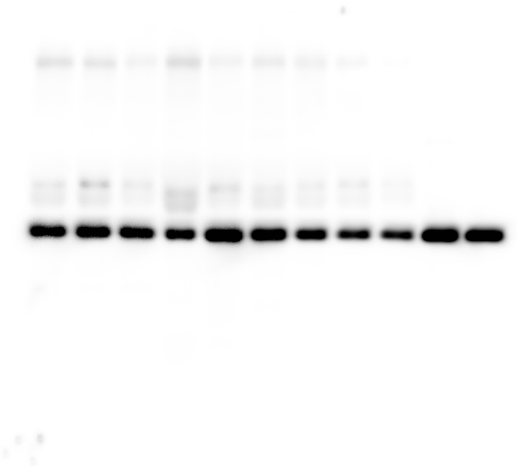

H = healthy
SE = subclinical endometritis
CLE = clinical endometritis
